# AP2σ Mutations Impair Calcium-Sensing Receptor Trafficking and Signaling, and Show an Endosomal Pathway to Spatially Direct G-Protein Selectivity

**DOI:** 10.1016/j.celrep.2017.12.089

**Published:** 2018-01-28

**Authors:** Caroline M. Gorvin, Angela Rogers, Benoit Hastoy, Andrei I. Tarasov, Morten Frost, Silvia Sposini, Asuka Inoue, Michael P. Whyte, Patrik Rorsman, Aylin C. Hanyaloglu, Gerda E. Breitwieser, Rajesh V. Thakker

**Affiliations:** 1 Academic Endocrine Unit, Radcliffe Department of Medicine, University of Oxford, Oxford, UK; 2 Diabetes Research Laboratory, Radcliffe Department of Medicine, University of Oxford, Oxford, UK; 3 Institute of Reproductive and Developmental Biology, Faculty of Medicine, Imperial College London, London, UK; 4 Laboratory of Molecular and Cellular Biochemistry, Tohoku University, Sendai, Japan; 5 Japan Science and Technology (JST) Agency, Precursory Research for Embryonic Science and Technology (PRESTO), Kawaguchi, Japan; 6 Center for Metabolic Bone Disease and Molecular Research, Shriners Hospitals for Children, St. Louis, MO, USA; 7 Geisinger Clinic, Weis Center for Research, Department of Functional and Molecular Genomics, Danville, PA, USA; 8 Institute of Metabolism and Systems Research, University of Birmingham, Birmingham, UK; 9 Centre for Endocrinology, Diabetes and Metabolism (CEDAM), Birmingham Health Partners, Birmingham, UK

**Keywords:** clathrin-mediated endocytosis, adaptor protein-2, GPCR, calcium signaling, hypercalcemia, endosomal signaling, G proteins

## Abstract

Spatial control of G-protein-coupled receptor (GPCR) signaling, which is used by cells to translate complex information into distinct downstream responses, is achieved by using plasma membrane (PM) and endocytic-derived signaling pathways. The roles of the endomembrane in regulating such pleiotropic signaling via multiple G-protein pathways remain unknown. Here, we investigated the effects of disease-causing mutations of the adaptor protein-2 σ subunit (AP2σ) on signaling by the class C GPCR calcium-sensing receptor (CaSR). These AP2σ mutations increase CaSR PM expression yet paradoxically reduce CaSR signaling. Hypercalcemia-associated AP2σ mutations reduced CaSR signaling via Gα_q/11_ and Gα_i/o_ pathways. The mutations also delayed CaSR internalization due to prolonged residency time of CaSR in clathrin structures that impaired or abolished endosomal signaling, which was predominantly mediated by Gα_q/11_. Thus, compartmental bias for CaSR-mediated Gα_q/11_ endomembrane signaling provides a mechanistic basis for multidimensional GPCR signaling.

## Highlights

• Disease-causing AP2σ mutants impair Gα_q/11_ and Gα_i/o_ signaling by CaSR, a class C GPCR
• AP2σ mutants impair trafficking of the CaSR
• The CaSR can signal by a sustained endosomal pathway
• CaSR differentially uses Gα_q/11_ and Gα_i/o_ for cell-surface and endosomal signaling

## Graphical Abstract

**Figure:**
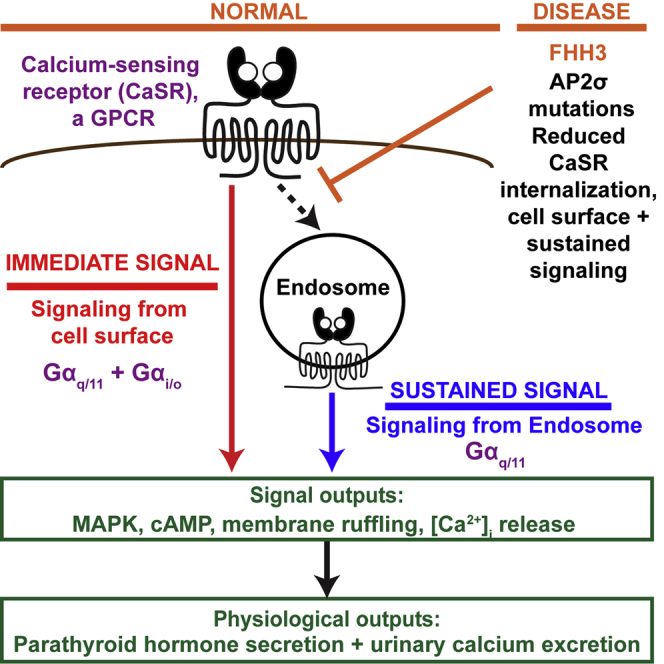

## Teaser

Gorvin et al. show that the class C GPCR calcium-sensing receptor (CaSR) mediates signaling from plasma membranes using Gα_q/11_ and Gα_i/o_ and from endosomes by using only Gα_q/11_. Adaptor protein-2 σ subunit (AP2σ) mutations impair CaSR internalization, leading to reduced sustained endosomal signaling and hypercalcemia in humans.

## Introduction

The G-protein-coupled receptor (GPCR) family is the largest family of signaling receptors, and GPCRs contribute significantly to fundamental cellular functions. The archetypal model of GPCR signaling has evolved from a single, cell-surface receptor activating a specific heterotrimeric G-protein pathway to a complex network in which receptors can activate multiple pathways, exhibit signal crosstalk, and display functional selectivity (Rosenbaum et al., 2009). This is illustrated by the calcium-sensing receptor (CaSR), a class C GPCR that is widely expressed and has calcitropic roles, i.e., regulation of extracellular calcium (Ca^2+^_e_) by the parathyroids, kidneys, and bone, and non-calcitropic roles such as inflammation, bronchoconstriction, wound healing, gastro-pancreatic hormone secretion, hypertension, and glucose metabolism (Hofer et al., 2000, Rossol et al., 2012, Yarova et al., 2015, Zietek and Daniel, 2015). Thus, the CaSR, which like other class C GPCRs has a large extracellular domain (ECD) containing the ligand binding sites, a seven-transmembrane domain, and a large cytoplasmic C-terminal domain (Katritch et al., 2013), forms dimers and couples to multiple G-protein subtypes (e.g., Gα_q/11_, Gα_i/o_, Gα_12/13_, and Gα_s_) to induce diverse signaling pathways. For example, the CaSR, when stimulated by elevations in Ca^2+^_e_, signals predominantly via Gα_q/11_ to activate phospholipase C (PLC), with consequent hydrolysis of phosphatidylinositol 4, 5-bisphosphate (PIP_2_), to the second messengers inositol 1, 4, 5-trisphosphate (IP_3_) and diacylglycerol (DAG) (Conigrave and Ward, 2013). IP_3_ acts upon IP_3_ receptors at the endoplasmic reticulum, allowing intracellular calcium (Ca^2+^_i_) mobilization into the cytosol, and DAG activates protein kinase C (PKC) signaling cascades, including mitogen-activated protein kinase (MAPK) pathways (Conigrave and Ward, 2013). CaSR has also been reported to signal via Gα_i/o_ to inhibit adenylate cyclase (AC) and reduce cyclic AMP (cAMP) (Conigrave and Ward, 2013), Gα_12/13_ to initiate cytoskeletal remodeling (Davies et al., 2006, Huang et al., 2004), and Gα_s_, leading to elevated cAMP levels in breast cancer cell lines (Mamillapalli et al., 2008).

These CaSR signaling pathways are dependent on CaSR cell-surface expression, which is regulated by a balance between its plasma membrane (PM) insertion and removal by endocytosis (Grant et al., 2011). The PM insertion of CaSRs involves an anterograde signaling pathway, referred to as agonist-driven insertional signaling (ADIS), in which CaSRs that are continuously produced at the endoplasmic reticulum are rapidly trafficked to and inserted at the PM in the presence of high Ca^2+^_e_ (Grant et al., 2011). Following activation, CaSRs have been reported to be endocytosed at a constant rate and targeted to the endo-lysosomal pathway for degradation (Grant et al., 2011). However, studies of patients with familial hypocalciuric hypercalcemia type-3 (FHH3), an autosomal dominant calcitropic disorder that is due to mutations of the σ subunit of the heterotetrameric adaptor protein-2 (AP2σ), which has a critical role in clathrin-mediated endocytosis (Nesbit et al., 2013b), have reported that FHH3-associated AP2σ mutations result in increased expression of the CaSR at the PM, which is paradoxically associated with reduced CaSR signaling via Gα_q/11_ (Nesbit et al., 2013a). FHH is a genetically heterogeneous disorder, which is characterized by mild to moderate elevations in serum calcium concentrations, low urinary calcium excretion, and normal to elevated circulating parathyroid hormone (PTH), and the three recognized types, FHH1, FHH2, and FHH3, are due to loss-of-function mutations of the CaSR, Gα_11_, and AP2σ, respectively (Hannan et al., 2016, Nesbit et al., 2013a, Nesbit et al., 2013b). FHH3-associated AP2σ mutations have been found to only occur at residue R15, and these comprise one of three missense mutations, R15C, R15H, or R15L, all of which would lead to a loss or weakening of a polar contact with the dileucine-based motif within cytoplasmic regions of membrane-associated cargo proteins and thereby impair their endocytosis (Kelly et al., 2008, Nesbit et al., 2013b). *In vitro* studies of these FHH3-associated mutations demonstrated that these AP2σ mutations decreased CaSR-mediated Gα_q/11_ signaling in response to elevations in Ca^2+^_e_ in cells expressing the mutants, despite increased CaSR cell-surface expression (Nesbit et al., 2013b).

To explain this paradox, we hypothesized that the FHH3-associated AP2σ mutations may be disrupting the contribution of endosomal sustained signaling to CaSR-dependent G-protein pathways, similar to those reported for some class A GPCRs—e.g., β2-adrenergic receptor (β2AR), dopamine receptor D1 (DRD1), thyroid-stimulating hormone receptor (TSHR), vasopressin receptor 2 (V2R), and luteinizing hormone receptor (LHR)—and class B GPCRs (e.g., parathyroid hormone 1 receptor, PTH1R) (Calebiro et al., 2009, Feinstein et al., 2013, Ferrandon et al., 2009, Irannejad et al., 2013, Jean-Alphonse et al., 2014, Kotowski et al., 2011). These components of the endocytic pathway, which have previously been considered endpoints for signaling, are now known to provide sites for sustained GPCR signals (Feinstein et al., 2013, Ferrandon et al., 2009), although the contribution of endomembrane sustained signaling to GPCR function has only been studied in the context of a single GPCR/G-protein pathway. However, GPCR signaling is complex, with many receptors (e.g., the CaSR) coupling to multiple G-protein-dependent and G-protein-independent pathways, and strategies to pharmacologically select for such specific pathways is increasingly recognized to be important (Rosenbaum et al., 2009). To further elucidate the role of the endocytic system in coordinating the pleiotropic activities of GPCRs, we investigated the effects of the FHH3-associated AP2σ mutations on the different G-protein pathways activated by CaSR and discovered that impaired internalization, by clathrin-mediated endocytosis of CaSR, differentially affects G-protein pathways of CaSR.

## Results

### Establishing AP2σ Mutant Stable Cell Lines

To investigate further the effects of FHH3-associated AP2σ mutations on CaSR signaling and trafficking, HEK293 cells stably expressing AP2σ wild-type (WT; R15) or mutant (C15, H15, and L15) proteins were established, using appropriate pcDNA3.1-*AP2S1* constructs that also had silent mutations, which rendered them resistant to AP2σ-targeted small interfering RNA (siRNA), thereby allowing study of the mutant protein in the absence of endogenous protein. The presence of AP2σ mutant proteins or siRNA-resistant mutations did not affect expression of endogenous AP2α, AP2β, or AP2μ that with the σ subunit form the heterotetrameric AP2; general clathrin-mediated endocytic functions such as transferrin uptake; or internalization and signaling of another GPCR, the β2AR (Figure S1). These stably expressing AP2σ cells were transiently transfected with pEGFP-CaSR-WT (AP2σ/CaSR-WT) cells (Figure S1). All AP2σ mutant/CaSR-WT cells, when compared to AP2σ-WT/CaSR-WT cells, had a decreased sensitivity to increases in Ca^2+^_e_-induced Ca^2+^_i_, which is mediated by Gα_q/11_, with significantly higher half-maximal effective concentration (EC_50_) values (Figure S2). These results, which are in agreement with our previous results from HEK293 cells transiently expressing AP2σ mutants (Nesbit et al., 2013b), demonstrate that these stably expressing AP2σ mutant cells have impaired Gα_q/11_-mediated, Ca^2+^_e_-induced Ca^2+^_i_ release and that they are therefore suitable for studying the effects of FHH3-associated AP2σ mutations on CaSR signaling pathways and trafficking.

### AP2σ Mutations Reduce Gα_q/11_ Signaling

**Figure 1:**
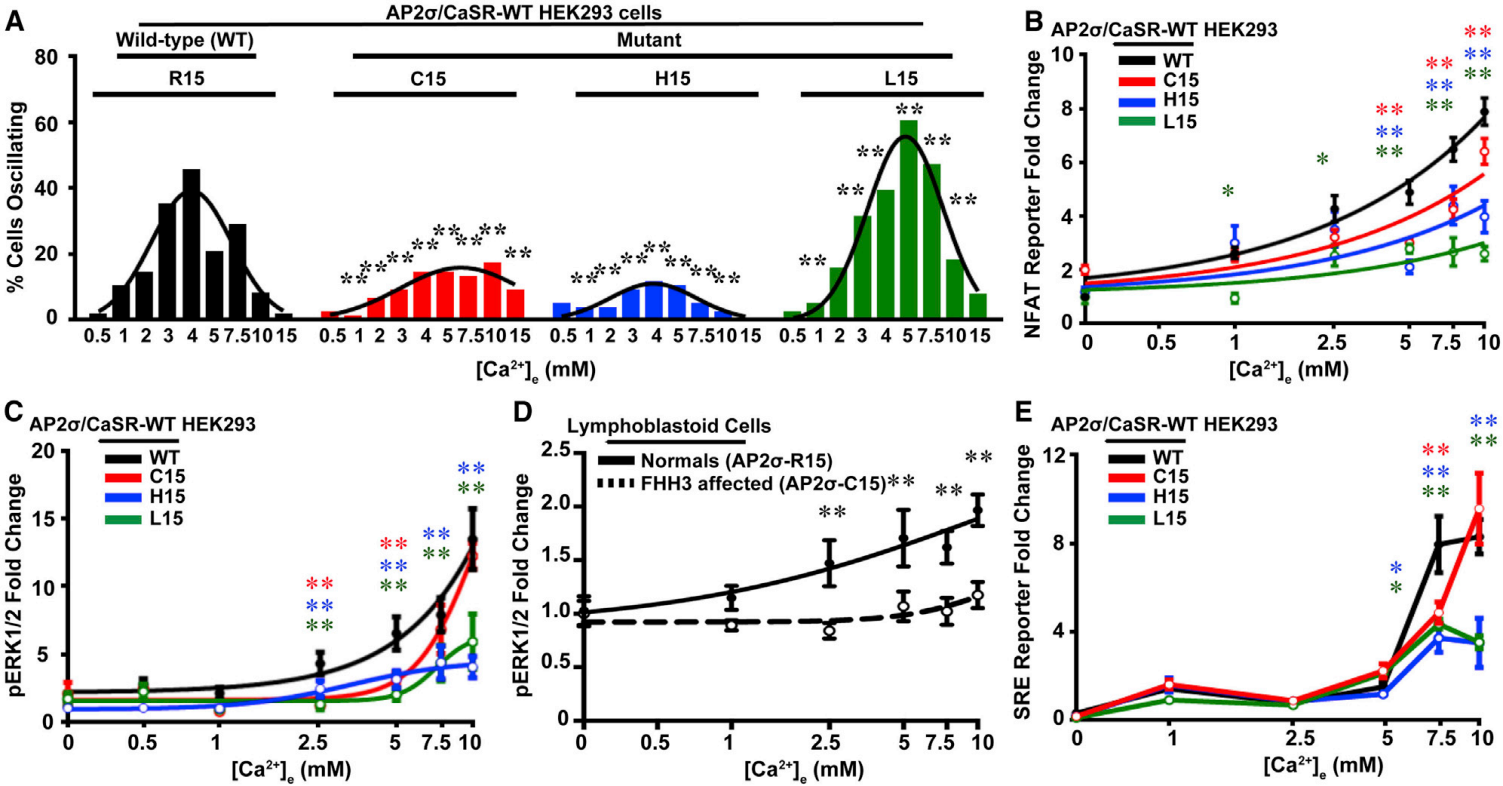
AP2σ-R15 Mutations Impair Gα_q/11_ Signaling (A) Number of oscillating cells measured by normalized Fura-2 ratios in response to increasing doses of Ca^2+^_e_ in single AP2σ/CaSR-WT HEK293 cells that stably expressed AP2σ-wild-type (WT; R15) or mutant (C15, H15, or L15) proteins and transiently expressed pEGFP-CaSR-WT (n = 36–50 cells from 9 to 10 transfections). ^∗∗^p < 0.02 versus WT (χ^2^ test) (Figures S1 and S2). (B) Ca^2+^_e_-induced NFAT luciferase reporter responses in AP2σ/CaSR-WT HEK293 cells (n = 8). (C) Ca^2+^_e_-induced phosphorylation of ERK1/2 (pERK1/2) measured by AlphaScreen (n = 4). AP2σ-WT/CaSR-WT cells had a dose-dependent increase in pERK1/2, which was reduced in AP2σ mutant/CaSR-WT cells within the range 2.5–5 mM Ca^2+^_e_ in C15 cells and 2.5–10 mM Ca^2+^_e_ in H15 and L15 cells. (D) Ca^2+^_e_-induced pERK1/2 responses measured by AlphaScreen in EBV-transformed lymphoblastoid cells from members of the FHH3 kindred in which affected members have AP2σ-C15 mutations (Figure S3). Unaffected (normal) relatives (AP2σ-R15) were used as controls (n = 4). (E) Ca^2+^_e_-induced SRE luciferase reporter responses in AP2σ/CaSR-WT HEK293 cells (n = 8). (B–E) Data are shown as mean ± SEM with ^∗^p < 0.05 and ^∗∗^p < 0.02 (two-way ANOVA of WT versus mutants).

We hypothesized that Ca^2+^_e_-induced Ca^2+^_i_ release of AP2σ mutant/CaSR-WT cells may be due to reduced calcium oscillations, and we assessed this by using single-cell microfluorimetry with the calcium-indicating dye Fura-2 in response to increasing concentrations (0–15 mM) of Ca^2+^_e_. CaSR-mediated Ca^2+^_i_ oscillations were observed to occur from 1 to 5 mM Ca^2+^_e_, consistent with previous reports, but mutant cells were found to have reduced frequencies, with the AP2σ-C15 and AP2σ-L15 cells requiring higher Ca^2+^_e_ concentrations to begin oscillating and AP2σ-H15 cells having oscillations with irregular amplitudes (Figures 1A and S2). Ca^2+^_i_ release activates transcription factors such as nuclear factor of activated T cells (NFAT) (Chakravarti et al., 2012). Investigation of the effects of the FHH3-associated AP2σ mutations on gene transcription, using an NFAT-response element (RE)-containing luciferase reporter construct, revealed that the AP2σ mutant/CaSR-WT cells had significantly reduced concentration-dependent increases in NFAT reporter activity when compared to AP2σ-WT/CaSR-WT cells (Figure 1B). Similarly, assessment of the accumulation of inositol monophosphate (IP_1_), an IP_3_ metabolite, revealed reduced IP_1_ in AP2σ mutant cells compared to AP2σ-WT cells (Figure S2), thereby indicating that the PLC-IP_3_-DAG pathway is impaired in AP2σ mutant cells.

CaSR Gα_q/11_-mediated signaling also activates MAPK pathways (Kifor et al., 2001). Investigation of the AP2σ mutant/CaSR-WT cells using AlphaScreen analyses of ERK1/2 phosphorylation (pERK1/2) in response to elevated Ca^2+^_e_ revealed them to have significant reductions in Ca^2+^_e_-induced pERK1/2 responses when compared to AP2σ-WT/CaSR-WT cells (Figure 1C). Moreover, pERK1/2 responses to increases in Ca^2+^_e_ were reduced in Epstein-Barr virus (EBV)-transformed lymphoblastoid cells from FHH3 patients with the AP2σ-R15C mutation (Figures 1D and S3), consistent with findings from AP2σ mutant/CaSR-WT cells. Expression of the AP2σ subunit genes and proteins was similar in lymphoblastoids from FHH3 patients with the AP2σ-R15C and unaffected relatives, indicating that the AP2σ-R15C mutation was not affecting the stability of the AP2 complex (Figure S3). ERK1/2 activates genes containing serum response elements (SREs) (Pi et al., 2002). Use of a SRE luciferase reporter revealed the AP2σ mutant/CaSR-WT cells have reduced SRE reporter activity (p < 0.02) (Figure 1E), with the more severe effects being observed in AP2σ-H15 and AP2σ-L15 mutant cells. Thus, these results demonstrate that the FHH3-associated AP2σ mutations cause a reduction in Gα_q/11_ signaling via both the IP_3_ and the DAG pathways.

### CaSR-Mediated cAMP Responses Are Altered by AP2σ Mutations

**Figure 2:**
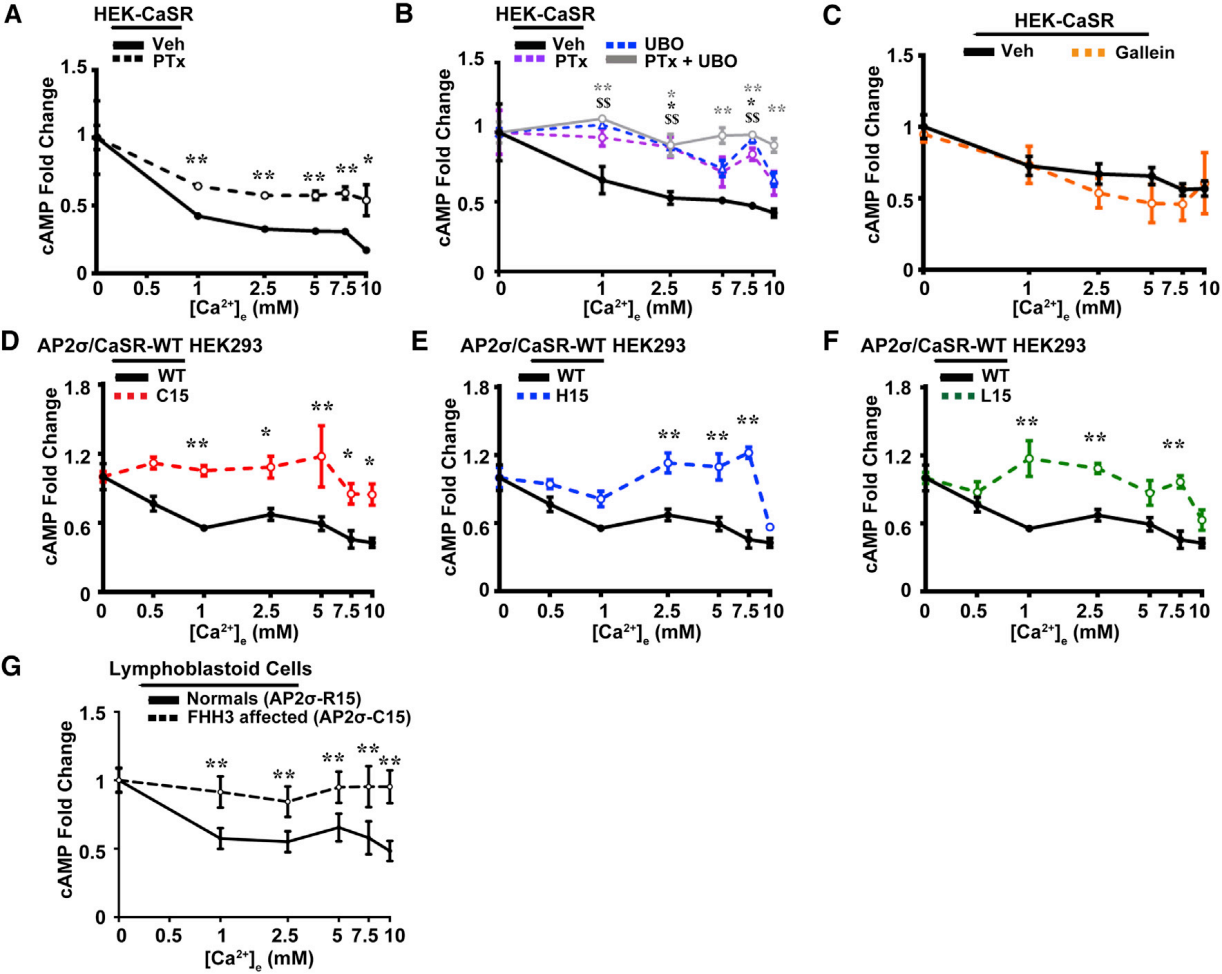
AP2σ-R15 Mutations Impair the Gα_i/o_ Signaling Pathway Ca^2+^_e_-induced cAMP inhibition was measured by AlphaScreen. (A) Effect of ethanol-diluent (vehicle, veh) or pertussis toxin (PTx) on Ca^2+^_e_-induced cAMP inhibition in HEK-CaSR-WT cells. PTx inhibits Gα_i/o_-mediated, Ca^2+^_e_-induced cAMP reductions (n = 4). (B) Effect of veh, PTx, the Gα_q/11_ inhibitor UBO-QIC (UBO), or combined PTx and UBO treatment on Ca^2+^_e_-induced cAMP inhibition in HEK-CaSR-WT cells (n = 4). (C) Effect of DMSO (vehicle, veh) or the Gβγ inhibitor gallein on Ca^2+^_e_-induced cAMP inhibition in HEK-CaSR-WT cells. Gallein did not significantly alter Ca^2+^_e_-induced cAMP responses when compared to vehicle (n = 4). (D–F) Ca^2+^_e_-induced cAMP inhibition in AP2σ-WT/CaSR-WT and AP2σ mutant/CaSR-WT HEK293 cells. AP2σ mutant cells—(D) C15, (E) H15, and (F) L15—had impaired responses when compared to WT (AP2σ-R15) cells (n = 8–12). (G) Ca^2+^_e_-induced cAMP inhibition in EBV-transformed lymphoblastoid cells from FHH3 patients, with AP2σ-C15 mutation, and unaffected (normal) relatives (Figure S3). Data are shown as mean ± SEM with ^∗^p < 0.05 and ^∗∗^p < 0.02 (two-way ANOVA comparing WT versus mutant in AP2σ HEK293 cells and normal versus FHH3 affected in lymphoblastoid cells). (B) shows vehicle versus PTx (black asterisk), UBO (dollar signs), and combined PTx and UBO (gray asterisks).

CaSR activation of the Gα_i/o_ pathway inhibits adenylate cyclase and reduces cAMP, and we assessed the effects of the FHH3-associated AP2σ mutations using AlphaScreen analysis to measure Ca^2+^_e_-induced cAMP responses. Ca^2+^_e_ was first confirmed to reduce cAMP responses, which were pertussis toxin (PTx) sensitive and therefore due to Gα_i/o_ signaling, in HEK293 cells stably expressing CaSR (HEK-CaSR) (Figure 2A). However, Gα_i/o_ inhibition only partially affected cAMP production, and treatment with UBO-QIC, an inhibitor of Gα_q/11_, revealed that the Ca^2+^_e_-induced reduction in cAMP was also sensitive to Gα_q/11_ inhibition, thereby indicating a hitherto unreported role for Gα_q/11_ (Figure 2B). Moreover, combined treatment of cells with both UBO-QIC and PTx halted all Ca^2+^_e_-induced reductions in cAMP (Figure 2B) indicating that G proteins other than Gα_q/11_ and Gα_i/o_ are unlikely to be involved in this CaSR pathway. However, UBO-QIC has been reported to inhibit Gβγ, in addition to Gα_q/11_ (Gao and Jacobson, 2016), but gallein, an inhibitor of Gβγ, had no effect on cAMP signaling (Figure 2C), thereby indicating that Gβγ is unlikely to have a role in CaSR-mediated cAMP reductions. Increases in [Ca^2+^]_e_ also led to a dose-dependent reduction in cAMP in AP2σ-WT/CaSR-WT cells, but not in AP2σ mutant/CaSR-WT cells, with cAMP in AP2σ-C15/CaSR-WT cells remaining at basal levels (Figure 2D) and with AP2σ-H15/CaSR-WT and AP2σ-L15/CaSR-WT cells responding with reductions in cAMP (Figures 2E and 2F). Moreover, lymphoblastoid cells from FHH3 patients with the AP2σ-R15C mutation, when compared to those from normal relatives, did not have Ca^2+^_e_-induced cAMP responses (Figure 2G), consistent with findings from the AP2σ-C15/CaSR-WT cells. Thus, the FHH3-associated AP2σ mutants reduce Gα_i/o_- and Gα_q/11_-mediated effects on cAMP responses.

### AP2σ Mutations Reduce Membrane Ruffling

**Figure 3:**
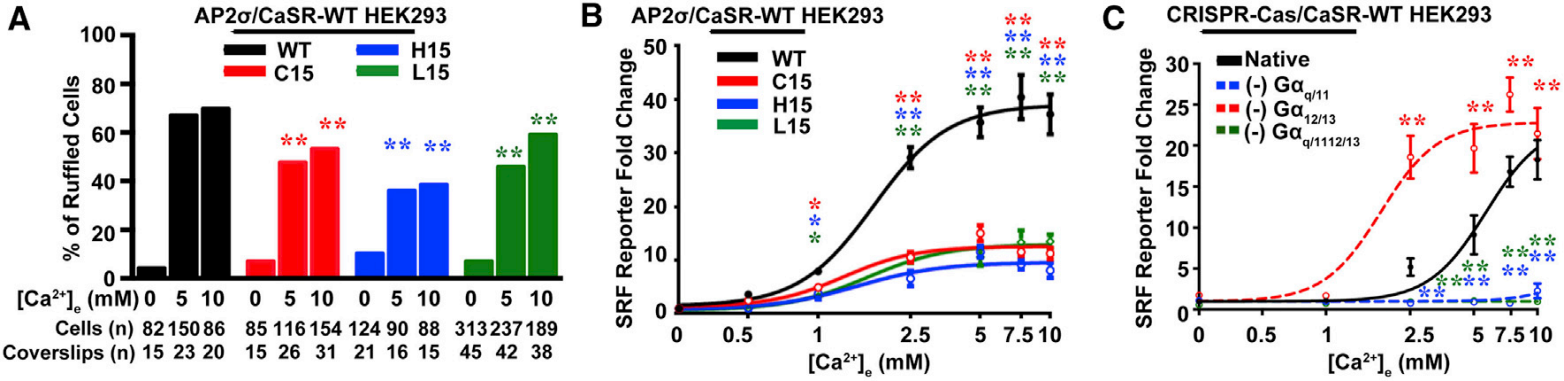
AP2σ-R15 Mutations Impair Membrane Ruffling via Reduction in Gα_q/11_ Signaling (A) Percentage of AP2σ/CaSR-WT cells with membrane ruffling (Figure S4) at each Ca^2+^_e_ concentration measured. Numbers (n) of cells—AP2σ-WT (R15) or mutant (C15, H15, or L15)—and coverslips are indicated. ^∗∗^p < 0.02 (χ^2^ test). (B) Ca^2+^_e_-induced SRF luciferase reporter activity (n = 8). Responses were reduced in AP2σ mutant cells. (C) Ca^2+^_e_-induced SRF luciferase reporter activity in native HEK293 cells or CRISPR-Cas gene-edited HEK293 knockout cells of Gα_q/11_, Gα_12/13_, or Gα_q/11/12/13_ transfected with pEGFP-CaSR-WT. (−) denotes genes deleted. SRF reporter activity was abolished in cells depleted of Gα_q/11_ and Gα_q/11/12/13_ but elevated in cells depleted of Gα_12/13_. Data are shown as mean ± SEM (n = 8) with ^∗^p < 0.05 and ^∗^p < 0.02 (two-way ANOVA of WT, or native, versus mutant).

CaSR has been reported to induce cytoskeletal changes such as membrane ruffling by both Gα_q/11_ and Gα_12/13_ signaling (Bouschet et al., 2007, Huang et al., 2004, Pi et al., 2002). We therefore investigated the effects of FHH3-associated AP2σ mutants on membrane ruffling, using AP2σ mutant/CaSR-WT cells and phalloidin-594 as an actin marker. Elevations of Ca^2+^_e_ increased membrane ruffling in AP2σ-WT and mutant cells, although AP2σ mutant cells had significantly reduced membrane ruffling compared to WT cells (p < 0.02) (Figures 3A and S4). Assessment of membrane ruffling-induced gene transcription (Tojkander et al., 2012) using a serum response factor (SRF)-RE reporter construct revealed AP2σ mutant cells to have significantly reduced SRF activity compared to AP2σ-WT cells (Figure 3B). Further investigation of SRF reporter assays in HEK293 cells transiently expressing CaSR but depleted of Gα_q/11_, Gα_12/13_, or Gα_q/11/12/13_ revealed SRF activity to be abolished in Gα_q/11_ and Gα_q/11/12/13_ knockout cells but to be significantly higher in Gα_12/13_ knockout cells than in native cells (Figure 3C). Moreover, quantification of membrane ruffling in Gα_12/13_ knockout cells and native HEK293 cells transiently expressing CaSR showed them to have similar levels of ruffling (Figure S4), thereby indicating the existence of Gα_12/13_-independent ruffling pathways. Overall, these results indicate that Ca^2+^_e_-induced membrane ruffling in HEK293 expressing CaSR is mediated by Gα_q/11_ signaling and that FHH3-associated AP2σ mutations, which impair Gα_q/11_ signaling, reduce membrane ruffling.

### AP2σ Mutations Impair CaSR Internalization and Differentially Affect CaSR Cell-Surface Expression, which Both Require Gα_q/11_

**Figure 4:**
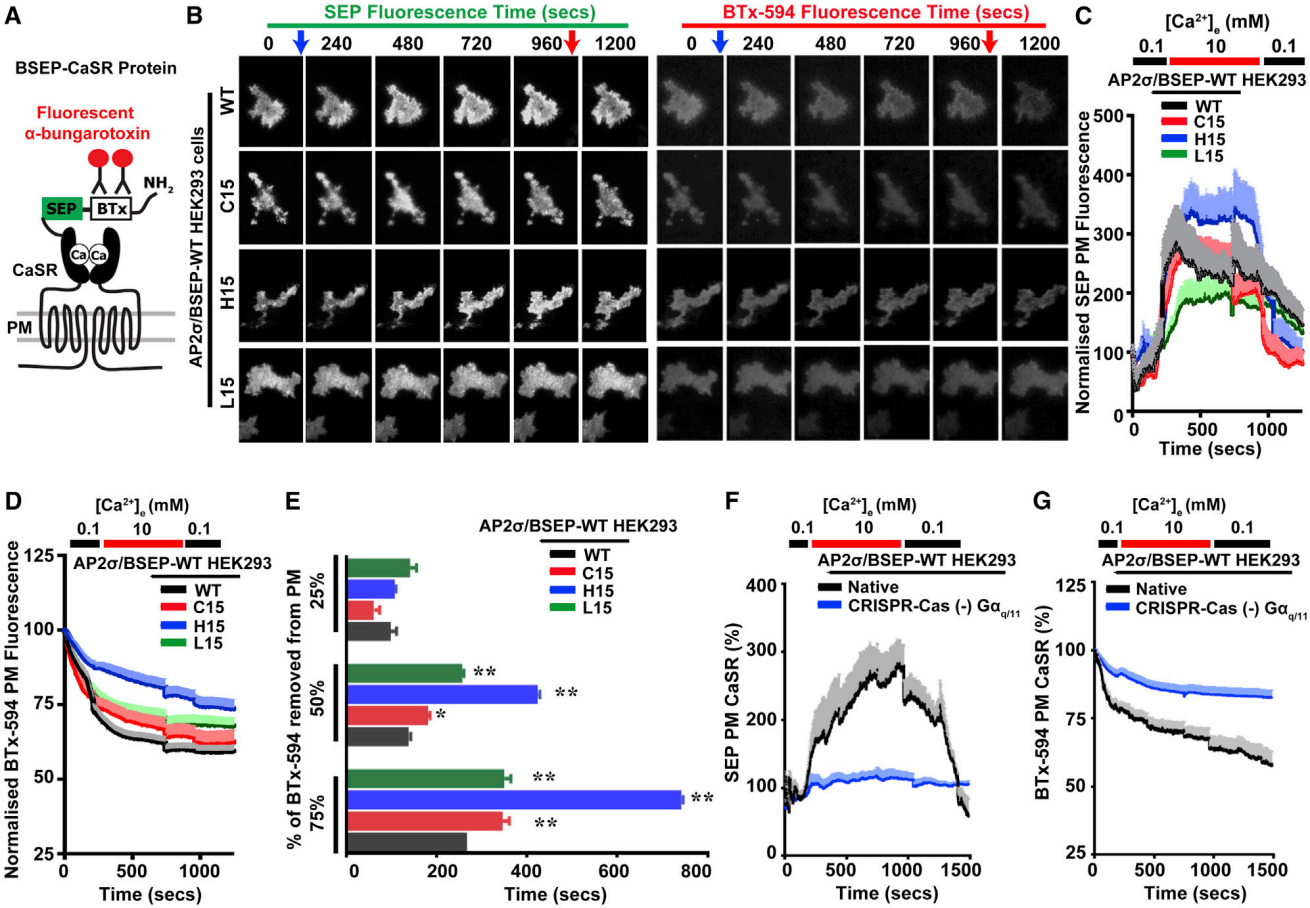
AP2σ-R15 Mutations Impair CaSR Internalization TIRF microscopy analyses in AP2σ-WT (R15) or mutant (C15, H15, or L15) HEK293 cells transfected with BSEP-CaSR. (A) Schematic diagram of BSEP-CaSR. BSEP-CaSR encodes CaSR with an N-terminal modification of a minimal bungarotoxin (BTx) binding site, to which BTx-594 binds to measure endocytosis, and superecliptic pHluorin (SEP), which maximally fluoresces at neutral pH and measures total cell surface CaSR. (B) TIRF microscopy images of SEP and BTx-594 fluorescence. Blue arrows indicate addition of 10 mM, and red arrows the return to 0.1 mM Ca^2+^_e_. (C and D) Quantification of fluorescence in each movie frame for (C) SEP and (D) BTx-594 images. [Ca^2+^]_e_ is shown above. Data are normalized to the fluorescence in the first frame of each movie (set at 100%). Data are shown as mean + SEM. (E) Time taken to reduce BTx-594 expression by 25%, 50%, and 75%. (F and G) TIRF microscopy analyses in native HEK293 cells or CRISPR-Cas gene-edited HEK293 cells of Gα_q/11_ transfected with BSEP-CaSR. Quantification of fluorescence in each movie frame for (F) SEP and (G) BTx-594 images. [Ca^2+^]_e_ is shown above. (−) denotes genes deleted. Cells depleted of Gα_q/11_ had impaired ADIS and endocytosis. Data are shown as mean + SEM with ^∗^p < 0.05 and ^∗∗^p < 0.02 for comparison to WT (two-way ANOVA).

FHH3-associated AP2σ mutations have been reported to result in increased CaSR cell-surface expression, which represents the net balance between its PM insertion by ADIS and removal by endocytosis (Grant et al., 2011). We therefore simultaneously measured the effects of the FHH3-associated AP2σ mutations on ADIS and endocytosis by transfecting AP2σ-WT and AP2σ mutant cells with a plasmid construct containing full-length CaSR, with an N-terminal modification that in tandem comprised a minimal α-bungarotoxin (BTx)-binding site to monitor endocytosis and superecliptic pHluorin (SEP) to monitor total cell-surface CaSR, referred to as BSEP-CaSR (Figure 4A) (Grant et al., 2011). Total internal reflection fluorescence (TIRF) microscopy was used to assess CaSR cell-surface expression under basal (0.1 mM Ca^2+^_e_) conditions or following exposure to 5 or 10 mM Ca^2+^_e_. Immediately before TIRF microscopy continuous recordings, cells were exposed to BTx with a fluorescent tag (BTx-594). AP2σ-WT and mutant cells expressed CaSR at the cell surface (Figures 4B and 4C), and both 5 and 10 mM Ca^2+^_e_ induced elevations in SEP fluorescence and reductions in BTx-594. These were greater at 10 mM Ca^2+^_e_, which was used for subsequent imaging experiments (Figures 4B, 4C, and S5). Thus, elevations in Ca^2+^_e_ increased CaSR PM insertion (Figures 4B and 4C), and returning Ca^2+^_e_ to basal conditions induced a reduction in cell surface CaSR, observed by a decline in SEP fluorescence (Figure 4C). Maximal SEP fluorescence in AP2σ-C15 cells was similar to WT, but AP2σ mutant L15 cells had reduced SEP fluorescence and H15 cells had significantly higher CaSR PM expression (p < 0.01, F test) (Figures 4B and 4C). All AP2σ mutant cells had slower declines in BTx-594 PM fluorescence when compared to AP2σ-WT cells, thereby indicating delayed internalization (Figure 4D). The time to internalize 75% of the BTx-594 at the PM was significantly increased from 268 s in AP2σ-WT to 346, 741, and 350 s in AP2σ-C15, AP2σ-H15, and AP2σ-L15 mutant cells, respectively (p < 0.05 to p < 0.02) (Figure 4E). This was greatest in the AP2σ-H15 cells, which may partly account for the very high CaSR PM expression in these cells (Figure 4C). Moreover, TIRF microscopy analysis of Gα_q/11_ knockout cells transfected with BSEP-CaSR showed that the Ca^2+^_e_-induced increase in SEP fluorescence (i.e., increased CaSR PM expression via ADIS) was lost and that CaSR internalization measured by BTx-594 fluorescence was severely impaired (Figures 4F and 4G). These findings indicate that Gα_q/11_ signaling is required for ADIS responses and that CaSR endocytosis requires a signal within the Gα_q/11_ pathway for its maintenance.

### CaSR Delayed Internalization Is due to Prolonged CaSR-Clathrin Colocalization in AP2σ Mutant Cells

**Figure 5:**
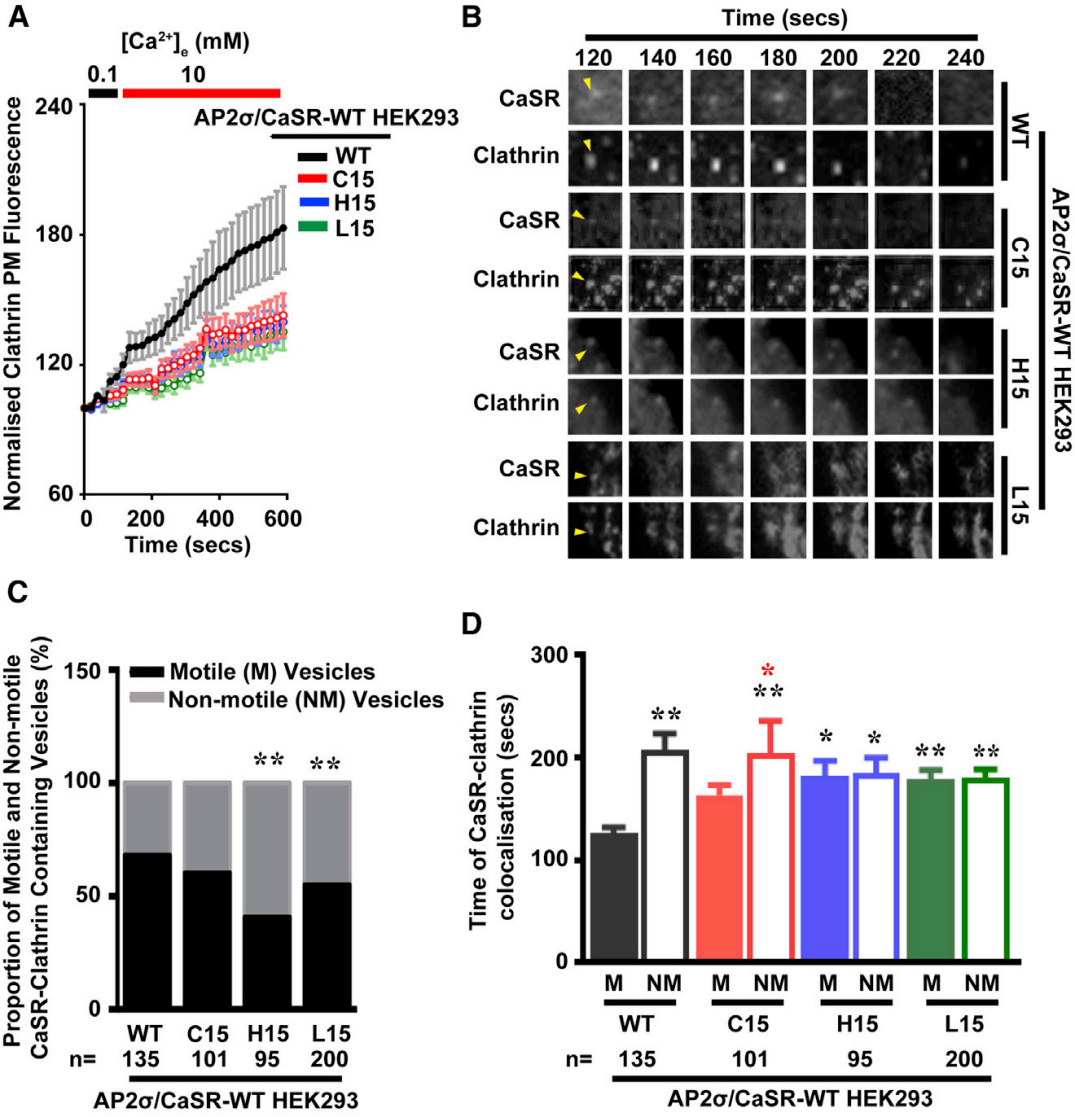
Impairments in CaSR Internalization Are due to Prolonged CaSR-Clathrin Colocalization TIRF microscopy analyses of colocalized CaSR (BSEP-CaSR) and Clathrin (dsRed-Clathrin) performed in AP2σ-WT (R15) or mutant (C15, H15, or L15) cells. (A) Quantification of clathrin fluorescence with changes in [Ca^2+^]_e_ (shown above). Data are normalized to the fluorescence in the first frame of each movie (set at 100%). Data shown as mean ± SEM. (B) Images of CaSR and clathrin expression in single vesicles (yellow arrow). (C and D) Proportion of motile (M) versus non-motile (NM) CaSR and clathrin-containing vesicles (C), and duration of colocalization between CaSR and Clathrin in individual (motile, M, filled box, and non-motile, NM, open box) vesicles (D). Data from 95 to 200 vesicles (n = 14–16 recordings) are expressed as mean ± SEM with ^∗^p < 0.05 and ^∗∗^p < 0.02 (two-way ANOVA) illustrated by black and red asterisks for WT motile versus mutant motile vesicles and C15 motile versus non-motile vesicles, respectively.

AP2σ mutants impair but do not abolish CaSR internalization (Figure 4), indicating that AP2 and clathrin are still recruited to the forming endocytic pit but that CaSR internalization occurs at a slower rate. We therefore predicted that the duration of colocalization between CaSR and clathrin may be prolonged, reflecting this slower internalization rate. We investigated this by transfecting AP2σ mutant and AP2σ-WT cells with BSEP-CaSR and dsRed-Clathrin and analyzed colocalization by TIRF microscopy. Clathrin fluorescence increased in the AP2σ-WT and AP2σ mutant cells during the TIRF microscopy recording, indicating that clathrin is recruited to the PM, although the increase in clathrin recruitment to the PM was significantly greater in AP2σ-WT than in AP2σ mutant cells (p < 0.02) (Figure 5A). Vesicles containing both clathrin and CaSR were analyzed for motility, because higher motility is associated with increased likelihood of viable endocytic events (Rappoport and Simon, 2003). Vesicles that had both CaSR and clathrin were highly motile in AP2σ-WT cells, which had a greater proportion of highly motile CaSR-clathrin-containing vesicles than AP2σ-H15 and AP2σ-L15 cells; instead, these AP2σ mutant cells had a significantly greater number of non-motile CaSR-clathrin-containing vesicles (p < 0.02) (Figures 5B and 5C). The reduced motility of the CaSR-clathrin-containing positive vesicles in AP2σ mutant cells would delay vesicle internalization and thereby likely prolong the colocalization of CaSR and clathrin in clathrin-coated pits. Assessment of the duration of CaSR-clathrin colocalization in individual vesicles revealed that all AP2σ mutant cells, when compared to AP2σ-WT cells, had prolonged CaSR-clathrin associations (Figure 5D). However, motile vesicles in AP2σ-WT and AP2σ-C15 cells had a significantly shorter duration of colocalization when compared to non-motile vesicles, indicating that these motile vesicles are likely resulting in endocytic events, although there was no significant difference between motile and non-motile vesicles in H15 and L15 cells (Figure 5D). These results indicate that CaSR internalization is impaired in AP2σ mutant cells at distinct stages of endocytosis by prolonged residency time at clathrin-coated pits and/or vesicles.

### CaSR Is Able to Induce Sustained Signaling from a Cytoplasmic Location

**Figure 6:**
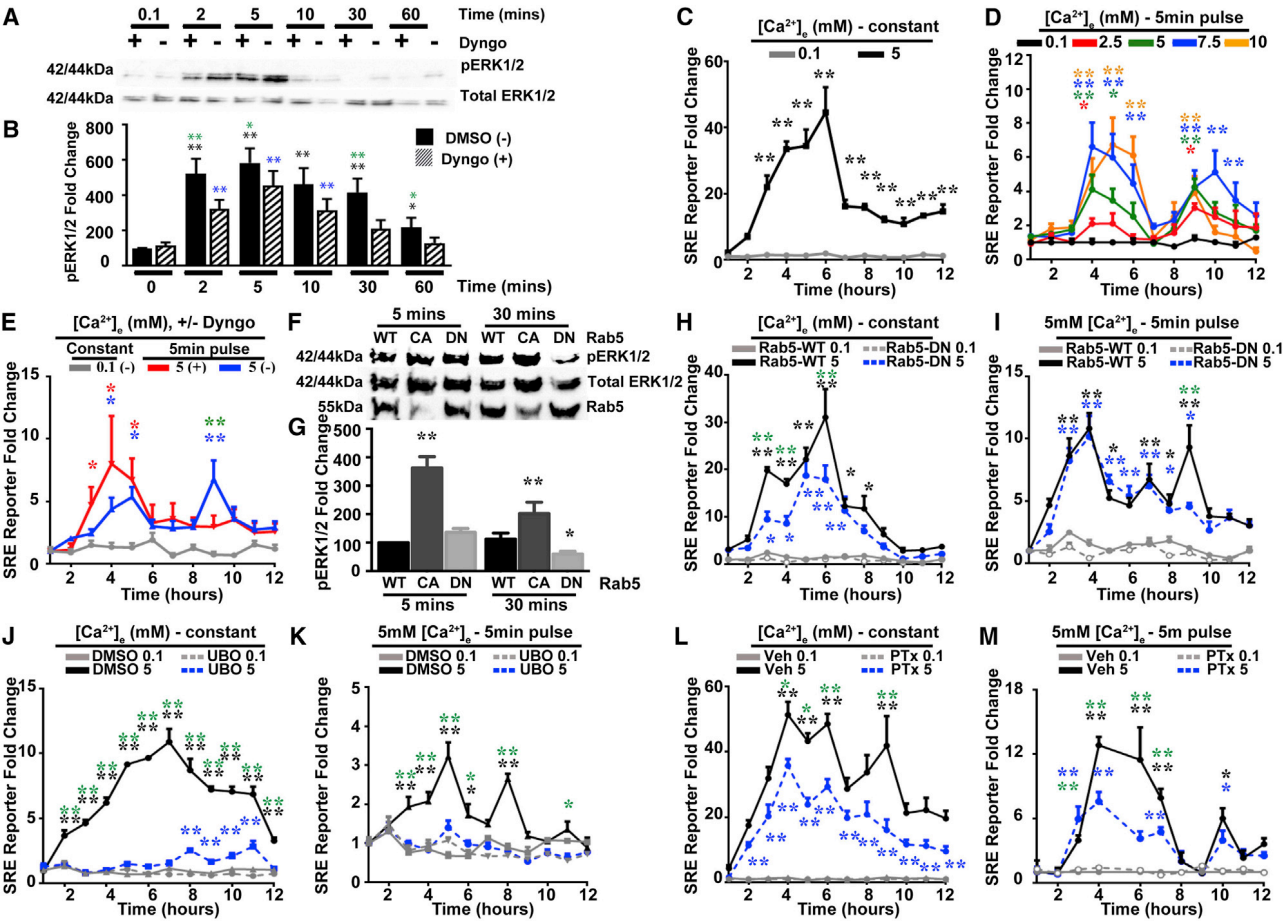
Second Signal of CaSR Is from the Rab5-Endosomal Internalization Pathway (A) Effects of dynamin inhibitor Dyngo on MAPK signaling by western blot analyses of pERK1/2 responses in HEK-CaSR cells treated with Dyngo (+) or DMSO (−), given a 5 min pulse of 5 mM Ca^2+^_e_, and then incubated in 0.1 mM Ca^2+^_e_. (B) Densitometry analysis showing data from blots (n = 8). Black and blue asterisks indicate p values of response versus response at 0 min for DMSO and Dyngo treated, respectively; green asterisks indicate DMSO versus Dyngo responses. (C) SRE luciferase reporter responses to treatment of either 0.1 or 5 mM Ca^2+^_e_ over 12 hr in HEK-CaSR cells. Asterisks indicate p values of response versus response to 0.1 mM (n = 4). (D) SRE luciferase reporter activity in response to 5 min pulses of 0–10 mM Ca^2+^_e_ in HEK-CaSR cells. Asterisks indicate p values of 0.1 mM responses versus 2.5 mM (red), 5 mM (green), 7.5 mM (blue), and 10 mM (yellow) (two-way ANOVA) (n = 4). Both initial and sustained peaks were enhanced by increasing concentrations of Ca^2+^_e_, which plateaued at 7.5 mM. Subsequent experiments were performed at Ca^2+^_e_ = 5 mM. (E) SRE luciferase reporter responses to a 5 min pulse of 0.1 or 5 mM Ca^2+^_e_ with DMSO (−) or Dyngo (+) in HEK-CaSR cells. DMSO (blue)-treated cells and Dyngo (red)-treated cells had a peak at 4 hr, while the second peak at 9 hr was abolished by treatment with Dyngo. Asterisks indicate p values of 0.1 mM Ca^2+^_e_ versus DMSO (blue) or Dyngo (red) and DMSO versus Dyngo (green) (two-way ANOVA). (F) Western blot analysis of pERK1/2 responses in HEK-CaSR cells exposed for 5 or 30 min to 5 mM Ca^2+^_e_. Cells were transiently transfected with the Rab5 WT (S34/Q79) or the constitutively active (CA; L79) or dominant-negative (DN; N34) Rab5 mutants. (G) Densitometric analyses of pERK1/2 in western blots (n = 4). Asterisks indicate p values of mutants compared to WT responses at each time point (two-way ANOVA). Rab5-CA had higher expression of pERK1/2 after 5 and 30 min of treatment, while Rab5-DN had lower pERK1/2 responses after 30 min. (H) SRE luciferase reporter responses to treatment of 0.1 or 5 mM Ca^2+^_e_ over 12 hr in HEK-CaSR cells transiently transfected with Rab5-WT or Rab5-DN mutant (n = 8). (I) SRE luciferase reporter response to 5 min pulses of 0.1 or 5 mM Ca^2+^_e_ in HEK-CaSR cells transiently transfected with Rab5-WT or Rab5-DN mutant (n = 8). (J) SRE luciferase reporter responses to treatment of 0.1 or 5 mM Ca^2+^_e_ over 12 hr in HEK-CaSR cells treated with DMSO or the Gα_q/11_ inhibitor UBO-QIC (UBO) (n = 4). (K) SRE luciferase reporter response to 5 min pulses of 0.1 or 5 mM Ca^2+^_e_ in HEK-CaSR cells treated with DMSO or UBO (n = 4). (L) SRE luciferase reporter responses to treatment of 0.1 or 5 mM Ca^2+^_e_ over 12 hr in HEK-CaSR cells treated with vehicle (Veh) or PTx, a Gα_i/o_ inhibitor (n = 8). (M) SRE luciferase reporter response to 5 min pulses of 0.1 or 5 mM Ca^2+^_e_ in HEK-CaSR cells treated with Veh or PTx (n = 8). Rab5-DN, UBO, and PTx all reduced constant Ca^2+^_e_ responses. In (H)–(M), asterisks show basal 0.1 mM Ca^2+^_e_ responses versus 5 mM Ca^2+^_e_ responses in Rab5-WT-, DMSO-, or Veh-treated cells (black); basal 0.1 mM Ca^2+^_e_ responses versus 5 mM Ca^2+^_e_ responses in Rab5-DN-, UBO-, or PTx-treated cells (blue); and Rab5-WT versus Rab5-DN, DMSO versus UBO, or Veh versus PTx (green) (two-way ANOVA). ^∗∗^p < 0.02, ^∗^p < 0.05. Rab5-DN and UBO reduced the sustained MAPK signal, while PTx had no effect on the sustained signal.

The FHH3-associated AP2σ mutations resulted in impaired CaSR-induced signaling (Figures 1, 2, and 3), despite increased CaSR cell-surface expression (Figure 4) due to delayed internalization. This led us to hypothesize that CaSR signaling may require, or be enhanced, by receptor internalization that would contribute to sustained (i.e., non-canonical) signaling. To test this hypothesis, we treated HEK293-CaSR cells with the dynamin-blocking agent Dyngo, which would abolish endocytosis and prevent endosomal signaling, and assessed their MAPK signaling responses by measurement of pERK1/2 to a 5 min pulse of 5 mM Ca^2+^_e_. pERK1/2 accumulated in Dyngo-treated and control DMSO-treated cells from 2 to 5 min and then rapidly decreased in Dyngo-treated cells, but not DMSO-treated cells; in the latter, pERK1/2 remained significantly increased at 30 min, indicating a potential sustained signaling response (Figures 6A, 6B, and S5). Loss of this sustained response in Dyngo-treated cells was not due to increased apoptosis, decreased proliferation, or inhibition of CaSR protein synthesis, because the sustained rise in pERK1/2 was not blocked by tunicamycin (Figure S5). The effects of this sustained pERK1/2 signaling on transcription were investigated by SRE reporter activity in HEK-CaSR cells treated with constant or 5 min pulsed elevations in Ca^2+^_e_. Constant treatment with 5 mM Ca^2+^_e_, when compared to 0.1 mM Ca^2+^_e_, resulted in rapid increases in SRE reporter activity that peaked between 4 and 6 hr, after which they rapidly reduced (Figure 6C). However, pulsed elevations with 5 and 7.5 mM, followed by incubation with basal 0.1 mM Ca^2+^_e_ for 0–12 hr, resulted in a peaked response between 4 and 6 hr that was followed by a second peaked response at 9 hr, consistent with a sustained signaling response (Figure 6D). Treatment with Dyngo abolished the second peaked response in HEK-CaSR cells given a 5 min pulse of 5 mM Ca^2+^_e_ (Figure 6E), thereby indicating that the sustained signaling response was likely originating from endosomes. An endosomal origin of this sustained response was further investigated by measuring pERK1/2 responses at 5 and 30 min in HEK-CaSR cells overexpressing the early endosome guanosine triphosphatase (GTPase) Rab5; a dominant-negative (DN) S34N guanosine diphosphate (GDP)-bound form, which delays endocytosis by retaining cargo in clathrin-coated pits (CCPs); and a constitutively active (CA) Q79L form, which enhances endocytic processes (Galperin and Sorkin, 2003, Stenmark et al., 1994). Rab5 was shown to be overexpressed by these constructs, and confocal microscopy showed that FLAG-CaSR-WT internalized over time in response to 5 mM Ca^2+^_e_ and partially colocalized with Rab5-WT-containing structures (Figure S6). Expression of Rab5-WT did not affect CaSR internalization, while the Rab5-DN protein delayed and reduced receptor internalization (Figure S6). In addition, HEK-CaSR cells expressing Rab5-CA when compared to Rab5-WT had enhanced pERK1/2 signals at 5 and 30 min, while Rab5-DN had reduced pERK1/2 signals at 30 min (Figures 6F and 6G). Furthermore, investigation of SRE reporter responses showed that the Rab5-DN reduced overall CaSR-driven SRE reporter activity (Figure 6H), which was due to loss of the sustained signal at 9 hr rather than reduction in immediate signaling (Figure 6I). MAPK signaling can be activated via Gα_q/11_ and Gα_i/o_ pathways (Figure S5) (Holstein et al., 2004). To assess the contribution of Gα_q/11_ and Gα_i/o_ signaling to sustained endosomal signaling, we measured SRE reporter activity in HEK-CaSR cells treated with UBO-QIC, an inhibitor of Gα_q/11_, or PTx, a specific inhibitor of Gα_i/o_ (Figures 6J–6M). In the presence of constant 5 mM Ca^2+^_e_, SRE reporter activity was reduced in UBO-QIC- and PTx-treated cells compared to vehicle-treated cells (Figures 6J and 6L). However, in cells treated with a 5 min pulse of 5 mM Ca^2+^_e_, UBO-QIC and PTx similarly impaired the early SRE response (Figures 6K and 6M), but only UBO-QIC reduced the sustained signal, which was not affected by PTx (Figures 6K and 6M). Thus, these findings indicate that Gα_i/o_ does not contribute to the sustained MAPK response from endosomes, which solely involves Gα_q/11_. The presence of Gα_q/11_ signaling pathway components in endosomes containing internalized CaSR was confirmed by using HEK293 cells transfected with FLAG-tagged CaSR and either Gα_q_-Venus or a known GFP-tagged biosensor of PIP_2_ (the lipid catalyzed by PLC), which contains the pleckstrin homology domain of PLC-delta (PH-PLC) (Stauffer et al., 1998). Before addition of 5 mM Ca^2+^_e_, colocalization of CaSR with either Gα_q_ or PH-PLC was observed only at the PM; however, following treatment with 5 mM Ca^2+^_e_ for 10 and 30 min, a subpopulation of CaSR-containing endosomes that colocalized with Gα_q_ or PH-PLC was detected, thereby indicating that internalized CaSR endosomes have Gα_q/11_ signaling components (Pearson’s correlation coefficients = 0.658 ± 0.027 for CaSR/Gα_q_ and 0.652 ± 0.024 for CaSR/PH-PLC at 10 min and 0.693 ± 0.049 for CaSR/Gα_q_ and 0.743 ± 0.059 for CaSR/PH-PLC at 30 min; n = 8–15) (Figure S6). To further assess the role of PLC in sustained signaling, we measured the effect of inhibitors of the PLC-DAG-IP_3_ pathway (Figure S7) on pERK1/2 responses. HEK-CaSR cells were pulsed with 5 mM Ca^2+^_e_ and then treated with DMSO or with U73122, GF-109203X (GFX), or 2-aminoethoxydiphenyl borate (2-APB), which inhibits PLC, PKC, or the IP_3_ receptor (IP_3_R), respectively (Figure S7). pERK1/2 accumulated in all cells from 2 to 5 min, and sustained responses were observed in DMSO-treated cells but were significantly reduced in U73122, GFX, and 2-APB-treated cells (Figure S7), thereby confirming the requirement of this Gα_q/11_ effector for sustained signaling. Finally, we assessed the effects of the scaffold proteins βarrestin-1 and βarrestin-2, which are important for endosomal signaling of GPCRs such as V2R and PTH1R (Feinstein et al., 2013, Wehbi et al., 2013), on the sustained signaling in HEK-CaSR cells and HEK293 cells that had deletions of βarrestin-1 and βarrestin-2, which were generated by CRISPR-Cas and stably overexpressed CaSR (Figure S7). The pERK1/2 and SRE reporter responses to a 5 min pulse of Ca^2+^_e_ in these cells lacking βarrestin-1 and βarrestin-2 showed no difference in responses when compared to WT cells, thereby indicating that βarrestin-1 and βarrestin-2 are not required for the CaSR sustained signal (Figure S7).

### AP2σ-R15 Mutations Impair Sustained Endosomal Signaling

**Figure 7:**
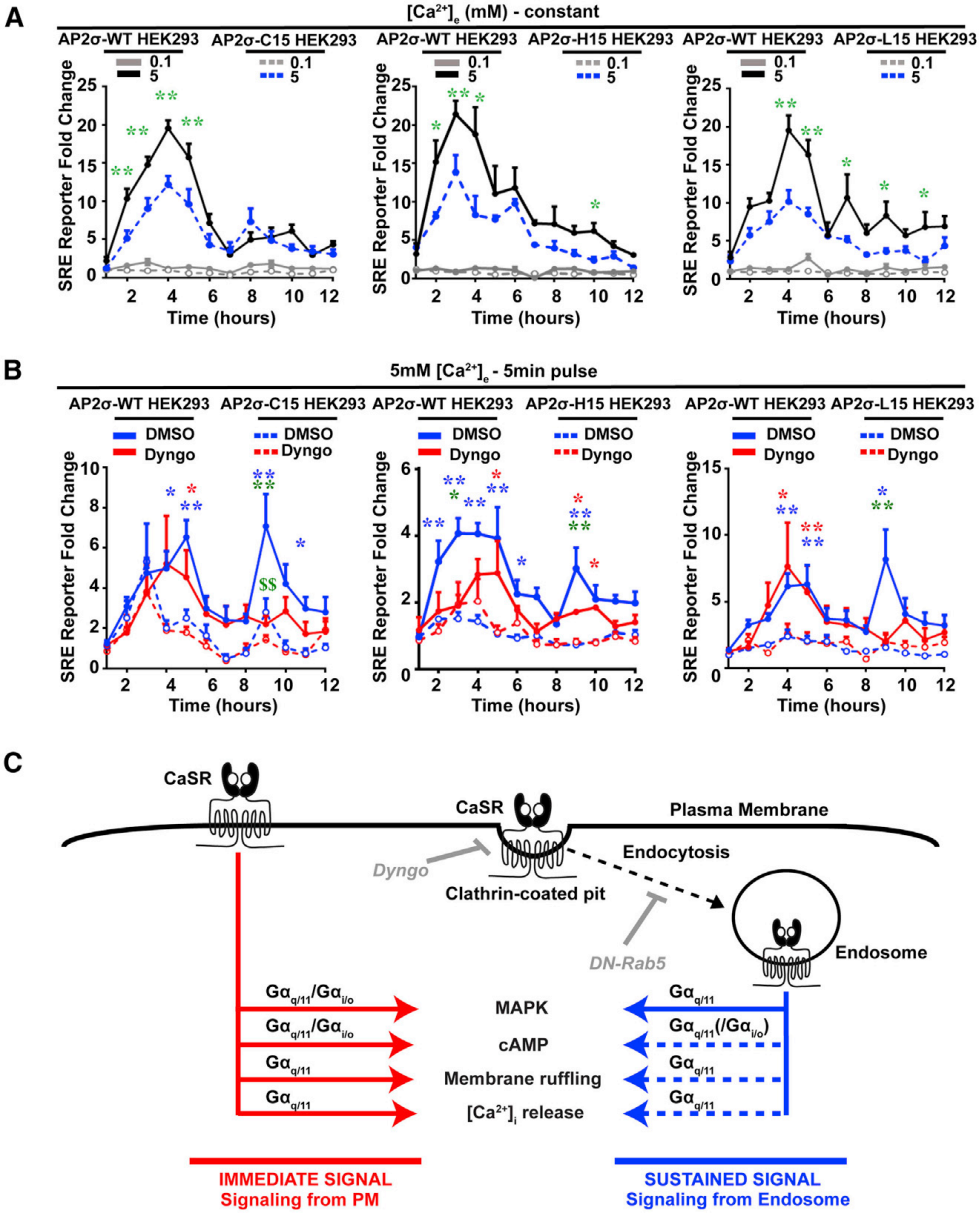
AP2σ-R15 Mutations Impair Sustained Signaling from Endosomes Studies of sustained signaling using SRE luciferase reporter assays in AP2σ-WT/CaSR-WT and AP2σ mutant/CaSR-WT HEK293 cells. (A) SRE luciferase reporter responses to constant treatment of 0.1 or 5 mM Ca^2+^_e_. Asterisks indicate p values for WT versus mutant responses (green) (n = 10–12). Statistical comparisons between 0.1 and 5 mM in the same cell type are not shown but were significantly greater for 5 mM in all cells between hours 2 and 11 (p < 0.05). Responses to 5 mM Ca^2+^_e_ were significantly greater in AP2σ-WT (R15) cells compared to AP2σ mutant (C15, H15, and L15) cells. Data are shown as mean + SEM with ^∗^p < 0.05, ^∗∗^p < 0.02 (two-way ANOVA). (B) SRE luciferase reporter response to 5 min pulses of 5 mM Ca^2+^_e_ treated with DMSO (blue) or Dyngo (red) in AP2σ-WT or AP2σ mutant cells (n = 10–12). Blue and red asterisks indicate WT versus mutant cells treated with DMSO and with Dyngo, respectively, and green asterisks and dollar signs indicate WT DMSO versus WT Dyngo and mutant DMSO versus mutant Dyngo, respectively. Data are shown as mean + SEM with ^∗^p < 0.05, ^∗∗^p < 0.02 or $$p < 0.02 (two-way ANOVA). (C) Summary of effects of AP2σ-R15 mutations on CaSR signaling pathways. CaSR is able to signal from the PM (red), using the Gα_q/11_ and Gα_i/o_ pathways to enhance MAPK signaling and to reduce cAMP, and increase membrane ruffling and Ca^2+^_i_ release, using Gα_q/11_. Following activation, CaSR is clustered into CCPs, before vesicle scission and internalization in clathrin-coated vesicles, and then into endosomes. Our results show that CaSR can induce sustained MAPK signaling (blue) from Rab5 endosomes and that FHH3-associated AP2σ mutations (C15, H15, and L15) impair all immediate signaling pathways (red) and impair or abolish sustained Gα_q/11_ signaling from the endosome, with responses of MAPK shown as a solid blue line (Figures 6 and 7) and other likely responses shown as a broken blue line and in parentheses. Pit invagination can be blocked by Dyngo, and maturation to Rab5-positive vesicles can be blocked by DN Rab5 mutant.

FHH3-associated AP2σ mutations impair CaSR signaling and internalization. We hypothesized that these AP2σ mutations were inhibiting sustained endosomal CaSR signaling and tested this by measuring the non-canonical SRE reporter responses in AP2σ-WT/CaSR-WT and AP2σ mutant/CaSR-WT cells treated with Dyngo, or overexpressing DN Rab5 (Figures 7A and S6). In the presence of constant 5 mM Ca^2+^_e_, SRE reporter responses were significantly higher in AP2σ-WT than in mutant cells, with peak expression occurring between 3 and 5 hr, in all cell lines (Figure 7A). Measurements of SRE reporter activity following a 5 min pulse of 5 mM Ca^2+^_e_ showed that the second Dyngo-sensitive peak was significantly reduced in C15 cells and abolished in H15 and L15 cells compared to WT cells (Figure 7B), thereby revealing that the FHH3-associated AP2σ mutations impaired early and sustained endosomal signaling. Moreover, the reduced sustained signaling in AP2σ-C15 cells was abolished by Rab5-DN, further demonstrating the endosomal origin of the sustained signaling (Figure S6). In summary, our results show that CaSR can induce sustained MAPK signaling from Rab5 endosomes and that FHH3-associated AP2σ mutations (C15, H15, and L15) impair Ca^2+^_i_ signaling, MAPK responses, cAMP reductions, and membrane ruffling and impair or abolish sustained signaling from the endosome.

## Discussion

Our study, which demonstrates that CaSR sustained signaling can occur by a non-canonical endosomal pathway, in addition to the established canonical PM pathway (Figure 7C), provides an explanation for the observed reduction in CaSR signaling that is paradoxically associated with increased CaSR PM expression because of FHH3-associated AP2σ mutations (Figures 1, 2, 3, and 4) (Nesbit et al., 2013b). Thus, in normal cells, total CaSR signaling comprises the output from the PM immediate and endosomal sustained pathways (Figure 7C); however, in cells with FHH3-associated AP2σ mutations, which impair CaSR internalization (Figure 4), the contribution from the endosomal pathway is lost or markedly reduced, with the remaining CaSR signaling occurring from the PM pathway (Figure 7C). Thus, CaSR endosomal signaling, which is sensitive to the dynamin-blocking agent Dyngo (Figure 6) and to DN mutants of the early endosomal protein Rab5 (Figure 6), occurs via Gα_q/11_ (Figures 5 and 6). Gα_q/11_ mediates alterations in Ca^2+^ (Figure 1), cAMP (Figure 2), membrane ruffling (Figure 2), and MAPK responses (Figure 1), all of which are impaired in cells expressing FHH3-associated mutations of AP2σ (Figures 1 and 2) that forms part of the heterotetrameric AP2 that plays a critical role in clathrin-mediated endocytosis. This CaSR sustained signaling is also not affected by tunicamycin (Figure S5), indicating a lack of requirement for newly synthesized CaSRs (Grant et al., 2011).

The three FHH3-associated AP2σ-R15 mutants, which all affected CaSR internalization—but not uptake of other clathrin-mediated endocytic cargos, such as transferrin or another GPCR, the β2AR (Figure S1)—had different effects on CaSR endocytosis and consequently different effects on signaling. Critically, these AP2 mutations unveiled that Gα_q/11_ signaling was more sensitive to alterations in CaSR endocytosis than the Gα_i/o_ pathway. Thus, the AP2σ-C15 mutant delayed CaSR internalization at the CCP (Dyngo sensitive) stage, whereas the AP2σ-H15 and AP2σ-L15 mutants inhibited CaSR internalization at the clathrin-coated vesicle (CCV) (Rab5-DN sensitive) stage. These milder effects of the AP2σ-C15 mutant on CaSR internalization still reduced Gα_q/11_ signaling, thereby indicating a possible threshold requirement for receptor occupancy within endosomes for activation of this G-protein pathway. In addition, the AP2σ-C15 mutant, but not AP2σ-L15 or AP2σ-H15, significantly affected Gα_i/o_ signaling at high [Ca^2+^]_e_, i.e., 10 mM (Figure 2), thereby suggesting that CaSR-mediated Gα_i/o_ signaling at high [Ca^2+^]_e_ is regulated at the CCPs, as opposed to Rab5 endosomes. Furthermore, Gα_i/o_, which can enhance MAPK signaling (Kifor et al., 2001), does not contribute to the sustained signal (Figures 6L and 6M), demonstrating the stronger requirement of receptor endocytosis for Gα_q/11_ signaling. In contrast, the AP2σ-L15 mutant, which had impaired CaSR internalization and abolished Gα_q/11_-mediated sustained MAPK signaling, resulting in the most severely reduced Gα_q/11_ signaling, had markedly reduced ADIS responses (Figure 4). These findings indicate not only that endosomal Gα_q/11_ signaling is critical for ADIS (Figures 4, 5, and 6) but also that there is a link between CaSR trafficking and signaling, thereby providing support for the proposed communication between endosomal compartments and the secretory machinery that links GPCR trafficking to maintain membrane receptor functionality (Clague and Urbé, 2001). Finally, the regulation of CaSR sustained signaling via its local environment within the endosome has yet to be established. Studies of the effect of different ligands, pH, receptor density, and tissue-specific differences that have previously been recognized for the CaSR (Conigrave and Ward, 2013, Quinn et al., 2004) require further investigation within the sustained signal context.

Our results reveal that the CaSR, a class C GPCR, induces sustained endosomal signaling (Figures 5, 6, and 7). This has similarities to reports for class A GPCRs, such as β2AR and LHR, which do not require βarrestin for endosomal and/or MAPK sustained signals (Irannejad et al., 2013, Jean-Alphonse et al., 2014). Moreover, GPCRs that use non-canonical signals often do so to facilitate biased agonism. This is illustrated by the class A GPCR V2R, which elicits sustained endosomal signals with vasopressin but rapid signals with oxytocin (Feinstein et al., 2013), and the class B PTH1R, which has sustained signals for PTH but rapid signals for PTH-related peptide (Ferrandon et al., 2009). Such spatial control of GPCR signaling has emerged as an important mechanism by which cells translate complex information into distinct cellular responses using a finite number of signal proteins. This is particularly the case for the CaSR, which has wide-ranging functions in diverse cell types, is able to couple to multiple G proteins, and responds to a variety of ligands. Thus, the ability to use immediate and sustained signaling pathways could account for some tissue- and cell-specific functions of the CaSR. For example, an immediate signaling pathway would likely facilitate the CaSR to rapidly respond to changes in [Ca^2+^]_e_ to restore calcium homeostasis by parathyroid and renal cells. In contrast, the role of CaSR in fetal development and bone mineralization (Goltzman and Hendy, 2015, Riccardi et al., 2013), which may require long-acting signals, may be facilitated by a sustained signaling pathway, providing a mechanism for the functional diversity of the CaSR.

In conclusion, our studies have demonstrated that the CaSR, a class C GPCR, mediates a sustained signal from an internal location that is likely to be the endosomes. In addition, our systematic characterization of CaSR signaling by such non-canonical, internalization-dependent (e.g., endosomal) pathways provides a paradigm for understanding how pleiotropic signaling pathways activated by a single GPCR can be resolved via spatially directed G-protein selectivity.

## Experimental Procedures

Detailed methods and information on constructs, oligonucleotides, and antibodies can be found in the Supplemental Experimental Procedures.

### Ethics Statement

Informed consent was obtained from individuals using protocols approved by local and national ethics committees, London, UK (MREC/02/2/93).

### Cell Culture

HEK-CaSR have been described (Nesbit et al., 2013b). HEK293 cells stably expressing AP2σ WT or mutant proteins were generated using a pcDNA3.1 construct (Invitrogen) containing full-length AP2σ cDNA with silent mutations to protect against AP2σ siRNA (Santa Cruz Biotechnology). Clonal cells were generated as described (Nesbit et al., 2013b), and cells with deletion of Gα_q_, Gα_11_, Gα_12_, Gα_13_, βarrestin-1, and βarrestin-2 by CRISPR-Cas have been described (Devost et al., 2017). Epstein-Barr virus-transformed lymphoblastoid cells were generated from members of the FHH3 kindred as described (Parkinson and Thakker, 1992). Transfections were performed with Lipofectamine 2000 (Invitrogen). Mutations within constructs were introduced by site-directed mutagenesis using Quikchange Lightning XL or Multi kits (Agilent Technologies) and confirmed by sequencing as described (Newey et al., 2013).

### Western Blot

For sustained signaling studies, cells were stimulated with 5 mM CaCl_2_ for 5 min, followed by incubation in 0 mM CaCl_2_ for 0–60 min. For studies with 30 μM Dyngo-4a (Abcam) (Jean-Alphonse et al., 2014), cells were pre-incubated for 30 min. For studies with 5 μM U73122 (Sigma), 1 μM GFX (Sigma), 100 μM 2-APB (Sigma), or 5 μg/mL tunicamycin (Sigma), compounds were added to the media and cells were incubated after calcium stimulation. For studies of Rab5 contribution to sustained signaling, 100 ng/mL mCh-Rab5-WT (Addgene plasmid 49201), mCh-Rab5 dominant negative (DN; S34N) or mCh-Rab5 CA (Q79L), were transfected 48 hr before western blot analysis. Western blots for pERK1/2 were then performed as described (Gorvin et al., 2017).

### Functional Assays

Transferrin assays were performed as described (Gorvin et al., 2013). IP_1_ assays were performed according to manufacturer’s instructions. For pERK1/2 AlphaScreen assays, cells were transfected with pEGFP-CaSR and treated with 0–10 mM CaCl_2_ for 5 min. For cAMP assays, cells were pre-treated with forskolin for 30 min. For inhibitor studies, cells were pre-treated with 300 ng/mL PTx or vehicle (ethanol) for 6 hr, 1 μM UBO-QIC or vehicle (DMSO) for 2 hr, or 15 μM gallein or vehicle (DMSO) for 15 min (Grant et al., 2011). AlphaScreen assays were performed as previously described (Gorvin et al., 2017). Apoptosis and proliferation were assessed using Caspase-Glo 3/7 and CellTiter Blue kits, respectively (Promega). For luciferase reporter assays, cells were transfected with pEGFP-CaSR, a reporter construct (pGL4-NFAT, pGL4-SRE, or pGL4-SRF), and a renilla construct (pRL) as described (Gorvin et al., 2017). Cells were treated with 0–10 mM CaCl_2_ for 4 hr. For sustained signaling studies, HEK-CaSR cells were transfected with luciferase construct and pRL and given one of four treatments: (1) 0.1 mM CaCl_2_, (2) 5 mM CaCl_2_ for the whole experiment (constant), (3) 5 min pulse of 5 mM CaCl_2_ followed by 0.1 mM CaCl_2_ with vehicle (DMSO) for the duration of the experiment, or (4) 5 min pulse of 5 mM CaCl_2_ followed by 0.1 mM CaCl_2_ with 30 μM Dyngo-4a for the duration of the experiment. Cells were pre-incubated with 1 μM UBO-QIC or DMSO for 2 hr or 10 μM forskolin (MP Biomedicals) and 300 ng/mL PTx (Sigma) or vehicle (ethanol diluent) for 6 hr (Avlani et al., 2013). Luciferase assays and Caspase-Glo 3/7 were measured on a Veritas luminometer (Promega), and CellTiter Blue was measured on a CytoFluor microplate reader (PerSeptive Biosystems).

### Fluorescent Imaging

For membrane ruffling, cells were transfected with pEGFP-CaSR, and actin was visualized with Phalloidin-594 (Molecular Probes) following treatment with 0, 5, and 10 mM Ca^2+^_e_. Cells were imaged on a Nikon Eclipse E400 wide-field microscope using adapted protocols (Bouschet et al., 2007, Davey et al., 2012). Single-cell microfluorimetry experiments were performed in AP2σ-WT or mutant cells transiently transfected with pEGFP-CaSR. Cells were loaded with Fura-2 (Molecular Probes) for 30 min and imaged on a Nikon TE2000 inverted microscope. Cells were perfused with extracellular bath solution with increasing CaCl_2_ concentrations. Fura-2 images were acquired using 340/380 nm excitation and 510 nm emission on μManager software (NIH). Methods for TIRF microscopy were adapted from previous studies (Grant et al., 2011, Hoppa et al., 2009). Images were obtained with an Olympus IX-81 TIRF microscope. To monitor CaSR internalization, cells were pre-incubated with BTx-594 and then perfused with 0.1 or 10 mM CaCl_2_ imaging solution. Images were captured at 10 frames/s in BSEP studies and 3 frames/s for clathrin studies. Images were acquired using CellˆR software (Olympus). Confocal imaging was performed in HEK293 cells using methods adapted from previous studies (Bouschet et al., 2007, Hanyaloglu et al., 2005). Images were captured using a confocal, laser-scanning microscope (Leica SP5). All images were analyzed using ImageJ (NIH).

### Statistical Analysis

Two-tailed unpaired t test, two-way ANOVA, χ^2^ test, Mann-Whitney U test, Pearson’s correlation coefficient, and F test were used to calculate statistical significance using GraphPad Prism 6 software. A p value < 0.05 was considered statistically significant. Statistical tests used are indicated in the methods in the Supplemental Experimental Procedures and figure legends.

## Supplemental Information

Document S1. Supplemental Experimental Procedures, Figures S1–S7, and Tables S1 and S2

Document S2. Article plus Supplemental Information
