# Supplementary material for: AP2σ Mutations Impair Calcium-Sensing Receptor Trafficking and Signaling, and Show an Endosomal Pathway to Spatially Direct G-Protein Selectivity
Source: Cell Rep. 2018 Jan 28;22(4):1054–66. doi: 10.1016/j.celrep.2017.12.089 (PMC5792449; doi:10.1016/j.celrep.2017.12.089)
Supplement: Document S1. Supplemental Experimental Procedures, Figures S1–S7, and Tables S1 and S2 [file mmc1.pdf]

Cell Reports, Volume *22*

Supplemental Information

AP2σ Mutations Impair Calcium-Sensing Receptor Trafficking and Signaling, and Show an Endosomal Pathway to Spatially Direct G-Protein Selectivity

Caroline M. Gorvin, Angela Rogers, Benoit Hastoy, Andrei I. Tarasov, Morten Frost, Silvia Sposini, Asuka Inoue, Michael P. Whyte, Patrik Rorsman, Aylin C. Hanyaloglu, Gerda E. Breitwieser, and Rajesh V. Thakker

SUPPLEMENTAL INFORMATION

AP2σ mutations impair calcium-sensing receptor trafficking and signaling, and reveal an endosomal pathway that spatially-directs G-protein selectivity

Caroline M Gorvin[1], Angela Rogers[1], Benoit Hastoy[2], Andrei I Tarasov[2], Morten Frost[1], Silvia Sposini[3], Asuka Inoue[4,5], Michael P Whyte[6], Patrik Rorsman[2], Aylin C Hanyaloglu[3], Gerda E Breitwieser[7], Rajesh V Thakker[1]

Supplemental Figures

FIGURE S1 Development of HEK293 AP2σ Stable Cell-lines, Related to Figure 1

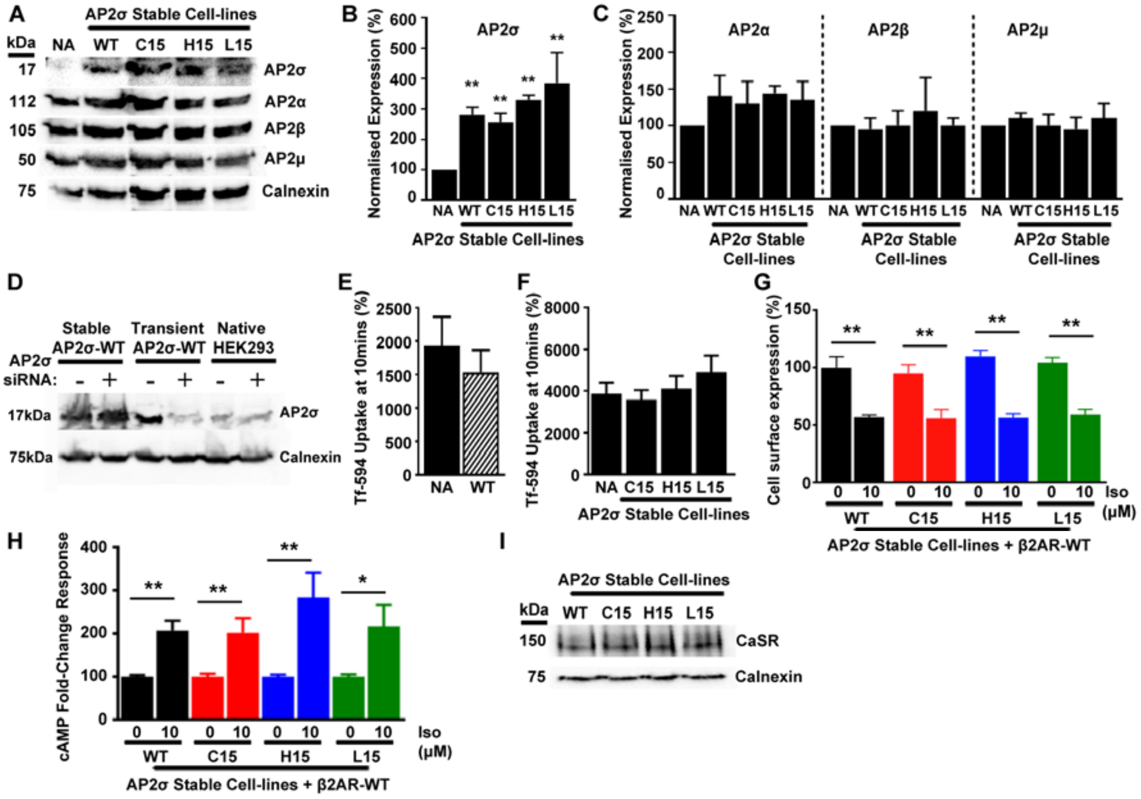

Stable overexpression of AP2σ protein (wild-type (WT, R15) or mutant (C15, H15, L15)) in HEK293 cells compared to parental native (NA) cells was confirmed by (A) Western blot analysis and (B) densitometric analysis from 4 independent blots. Data are shown as mean+SEM. **$p<0.02$ (2-way ANOVA); NA cells vs. test cells. Note that the Western blots detecting AP2σ were exposed for 2 seconds, whereas those for AP2α, AP2β and AP2μ were exposed for 1 minute. This is because the expression of AP2σ in the cells stably expressing AP2σ was ~200% greater than that of the other subunits and of AP2σ in the NA cells, which appear therefore to have a low expression. Longer exposure of these AP2σ Western blots would reveal the expression of AP2σ in NA cells, but would lead to dense (over-exposed) bands in the AP2σ stably expressing cells that would not have allowed any meaningful quantification by densitometry. (C) Expression of AP2 subunits α, β and μ, was unaffected by AP2σ overexpression. These findings are in contrast to other studies in which deletion of one AP2 subunit affected the expression and stability of other AP2 components (Boucrot et al., 2010; Mitsunari et al., 2005), but are consistent with our studies of mice with a heterozygous N-ethyl-N-nitrosourea-induced splice-site mutation, which results in loss of 17 amino acids from AP2σ, and a ~50% reduction in AP2σ protein, in which the expression of AP2 α, β and μ are similar to wild-type mice (Gorvin et al., 2017b). Data are from Western blot densitometry analysis of 4 independent blots and are shown as mean+SEM with 2-way ANOVA analysis. Calnexin was used as a housekeeping protein. (D) Western blot analysis confirmed that siRNA to AP2σ is able to selectively knockdown AP2σ expression in native HEK293 cells and in native HEK293 cells transiently expressing AP2σ-WT (Transient), but not in AP2σ stable cell-lines. Calnexin was used as a housekeeping protein. (E-F) Transferrin (Tf) uptake following 10 minutes of Tf-594 treatment in (E) Native (NA) HEK293 cells stably expressing AP2σ-WT protein (WT, R15), and (F) HEK293 cells stably expressing AP2σ-WT (R15) or AP2σ-mutant proteins (C15, H15 or L15). Data is presented as a percentage compared to uptake at 0 minutes. Uptake of fluorescent transferrin is not affected by overexpression of the AP2σ protein or by AP2σ mutation. Data are from n=8 biological replicates with two-way ANOVA analysis. (G) Cell surface expression of FLAG-β2-adrenergic receptor (β2AR) measured by ELISA, and (H) cAMP signaling by β2AR in response to 0μM or 10μM isoproterenol, assessed in AP2σ-WT or AP2σ-mutant cell-lines. Treatment with Iso led to reduced cell surface expression and increased cAMP responses in all AP2σ-WT and AP2σ-mutant cells, and no significant differences were seen between responses when comparing AP2σ-WT and AP2σ-mutant cells. Data shows mean+SEM and represents data from n=6-12

biological replicates; **p<0.02 for 0 vs 10 μM (2-way ANOVA). (I) Western blot analysis confirmed that the pEGFP-CaSR-WT construct expressed CaSR-WT at similar levels when transfected into AP2σ-WT or AP2σ-mutant cells.

FIGURE S2 Ca^{2+}_e-induced Ca^{2+}_i Signaling is Impaired in AP2σ-Mutant Cells Transiently Expressing CaSR due to Delayed Oscillation Events, Related to Figure 1

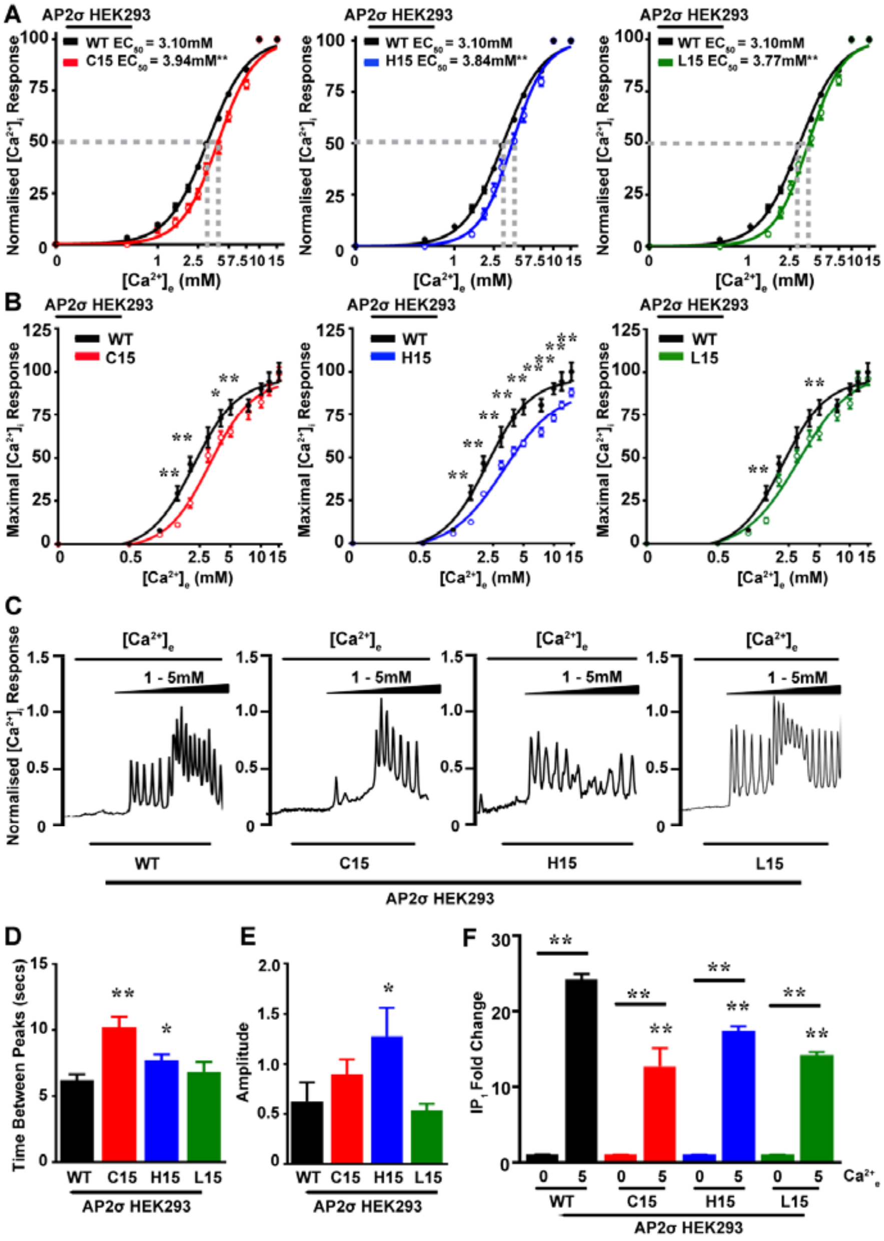

(A) Normalized Fura-2 ratios in response to increasing doses of Ca^{2+}_e in single cells expressing AP2σ-WT (R15) or mutant (C15, H15 or L15) proteins and transiently transfected with pEGFP-CaSR. Data are shown as mean+95% confidence intervals (CI), n=36-50 cells from 9-10 transfections. Ca^{2+}_e-induced Ca^{2+}_i responses are rightward-shifted in mutants resulting in higher half-maximal Ca^{2+}_i responses (EC50s) (dashed grey line) when compared to wild-type cells. EC_{50} values (WT, 3.10mM (95% confidence interval (CI) 3.05-3.16), C15, 3.94mM (95% CI 3.81-4.06), H15, 3.84mM (95% CI 3.70-3.98), L15, 3.77mM (3.61-3.91), $p<0.02$ for all). ** $p<0.02$ mutant vs WT (*F*-test). (B) Maximal Ca^{2+}_e-induced (Emax) Ca^{2+}_i responses. Emax values were significantly lower at $[Ca^{2+}_e]$ in the range 2-10mM in AP2σ-H15 cells, and in the range 2-5mM in AP2σ-C15 and AP2σ-L15 cells. The maximal signaling response of a GPCR is influenced by its ability to couple to its cognate G-protein, and thus it is possible that the AP2σ-H15 mutant impairs coupling and/or dissociation of $G\alpha_{q/11}$ from the CaSR more than the AP2σ-C15 and –L15 mutants. Data is expressed as mean±SEM. ** $p<0.02$ vs WT. (C) Representative images of calcium oscillations observed in AP2σ-WT (R15) and AP2σ-mutant (C15, H15, L15) cells. (D) Frequency of oscillations was reduced in AP2σ-C15 and –H15 cells, while (E) amplitude was only affected in

AP2σ-H15 cells. (F) Accumulation of the IP_3 breakdown product IP_1 in AP2σ-WT/CaSR-WT and AP2σ-mutant/CaSR-WT HEK293 cells in response to 0mM or 5mM Ca^{2+}_e. AP2σ-mutant cells had impaired responses compared to AP2σ-WT cells. Panels (B-D) data are shown as mean+SEM and are from n = 36-50 cells from 9-10 independent transfections; *$p<0.05$ and **$p<0.02$ for mutant vs. WT (Mann-Whitney U-test (panel B) and 2-way ANOVA (panels D and E)). Panel F shows mean+SEM and represents data from n=4 biological replicates; **$p<0.02$ for mutant vs. WT (2-way ANOVA).

FIGURE S3 Normal Expression of AP2 Subunits in FHH3 Patients and their Unaffected (Normal Control) Relatives, Related to Figure 1 and 2

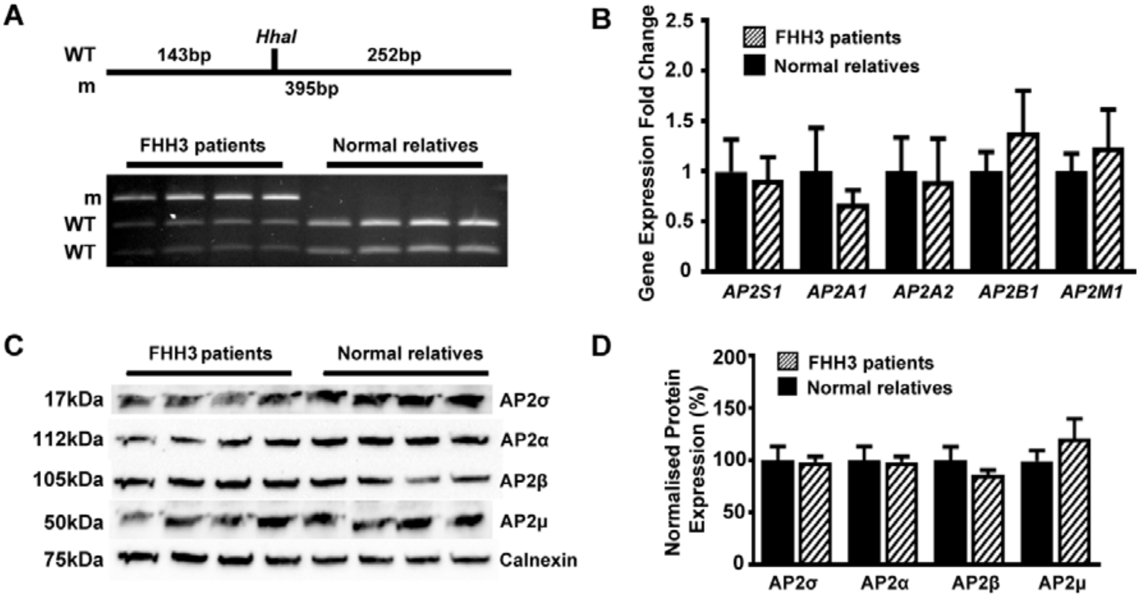

Confirmation of the AP2σ mutation (R15C) in EBV-transformed lymphoblastoid cells from patients of a previously described FHH3 kindred (McMurtry et al., 1992; Nesbit et al., 2013). (A) Restriction endonuclease map showing the cleavage site of *HhaI* that is disrupted by the mutation. Thus, the full-length WT is cleaved once with *HhaI* to yield two products at 143bp and 252bp. The *HhaI* site is lost in AP2σ-mutants (m). Lower panel shows the restriction endonuclease digests of DNA from PCR products of *AP2S1* exon 2, which shows that patients with FHH3, who are heterozygous for the AP2σ R15C mutation, have a mutant (m) uncleaved (395bp) and wild-type (WT) cleaved products (143bp and 252bp); whereas normal relatives are homozygous for the cleaved WT products only. (B) qRT-PCR analysis of genes (*AP2S1*, *AP2A1*, *AP2A2, AP2B1,* and *AP2M1*) which encode the AP2 σ, α, β, and μ subunits, respectively in EBV-transformed lymphoblastoid cells from FHH3 patients and normal relatives. Presence of the AP2σ mutation has no effect on expression levels of any of the subunits. Data is expressed as mean+SEM (n=4). (C) Western blot analysis of the AP2 subunits in protein lysates extracted from EBV-transformed lymphoblastoids of the FHH3 patients and normal (control) relatives, and (D) densitometric analyses of Western blots (n=4). Calnexin was used as a housekeeping protein. The expression of the AP2 subunits was not significantly different in the cells of patients and normal relatives. Expressed as mean+SEM (n=4). Statistical analyses were performed by 2-way ANOVA and student's t-test.

Figure S4 Membrane Ruffling is Impaired in AP2σ-Mutant Cells, Related to Figure 3

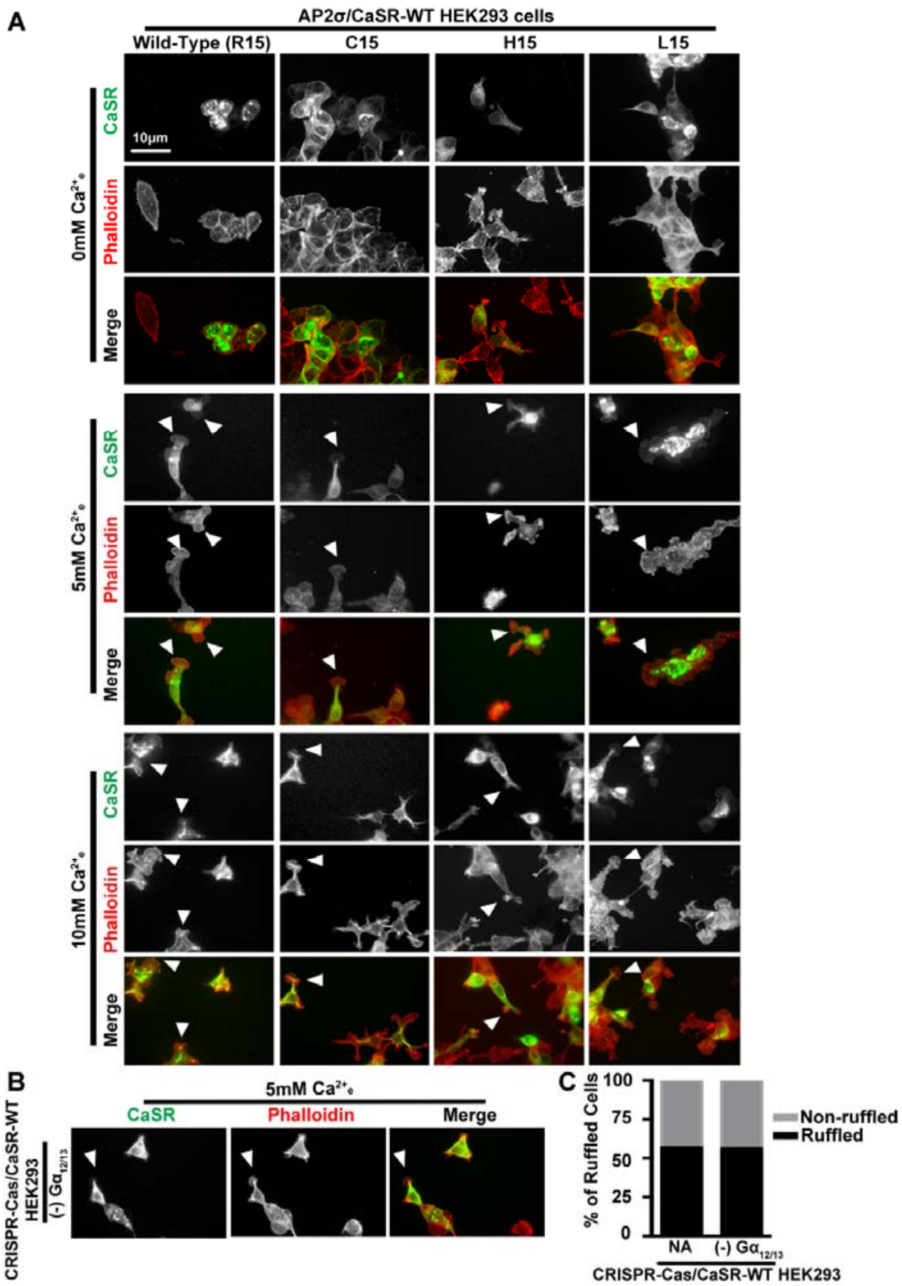

(A) Representative images of Ca^{2+}_{e}-induced membrane ruffling in AP2σ-wild-type (WT, R15) or mutant (C15, H15, L15) cells transfected with pEGFP-CaSR-WT to visualize the receptor and Phalloidin-594 as an actin marker (n = 36-50 cells from 9-10 transfections). (B) Representative images of Ca^{2+}_{e}-induced membrane ruffling in CRISPR-Cas generated HEK293 $G\alpha_{12/13}$ knockout cells ((-)$G\alpha_{12/13}$) transfected with pEGFP-CaSR-WT. (C) Percentage of cells with ruffled and non-ruffled membranes, at 5mM Ca^{2+}_{e}, in mutant ((-)$G\alpha_{12/13}$) cells did not differ significantly from that in native (NA) cells, thereby indicating that membrane ruffling, in cells expressing CaSR, can occur in the absence of $G\alpha_{12/13}$. These similar levels of membrane ruffling in $G\alpha_{12/13}$ knockout cells and native cells expressing the CaSR, was associated with increased SRF activity (Figure 3C) and this suggests that: other signaling inputs, which are downstream of $G\alpha_{q/11}$, and affect SRF transcription but not membrane ruffling, may be involved and these may include MAPK pathways via p38, JNK and ERK, that are activated by CaSR and enhance SRF (Kifor et al., 2001; Zhang and Liu, 2002), or Ca^{2+}_{i} that is utilized by muscarinic receptors of Jurkat T-lymphocytes (Lin et al., 2002); and that the SRF reporter assay may be more sensitive than the membrane ruffling assay in detecting subtle changes in signaling pathways.

FIGURE S5 Optimisation of TIRF-M Conditions and Signaling Assays for Observation of ADIS and Endocytosis, and Sustained Signaling, Related, to Figure 4 and 6

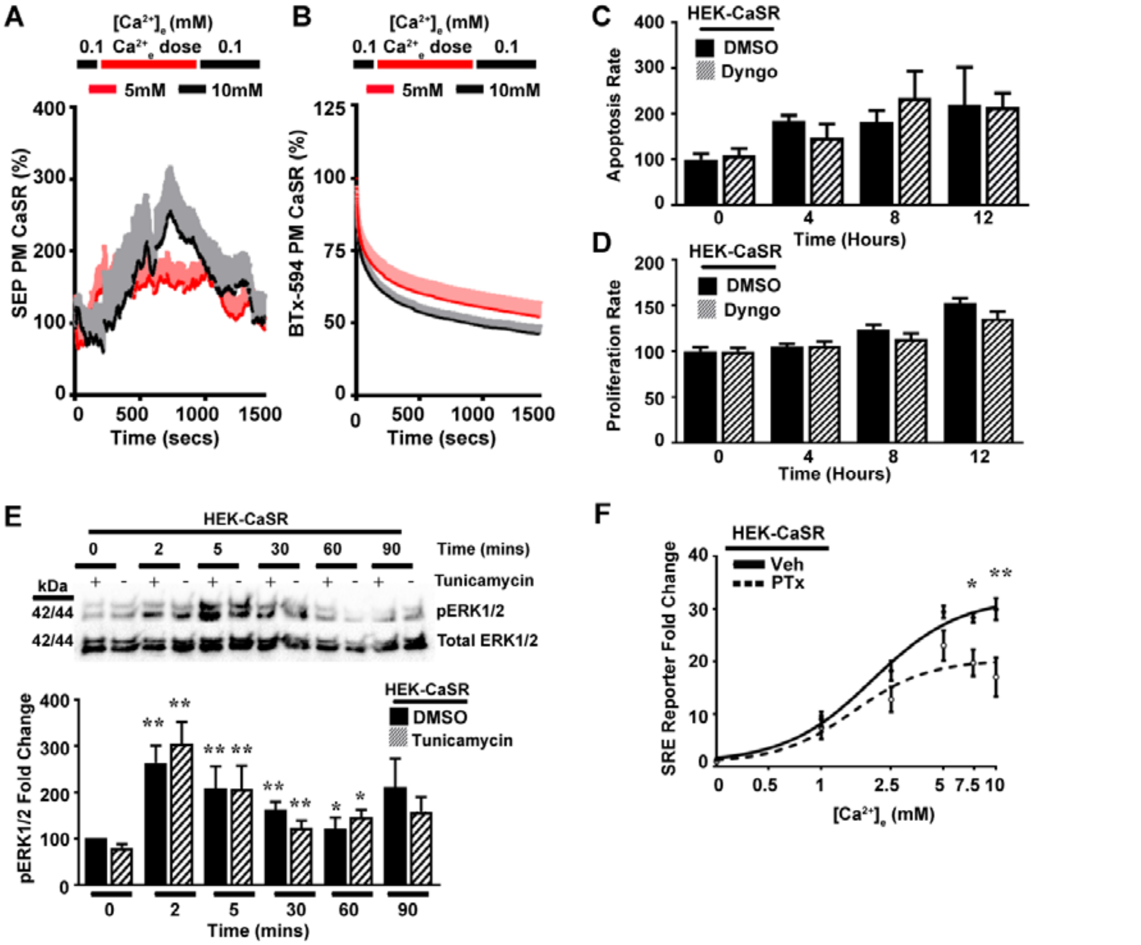

TIRF-M analyses of (A) SEP and (B) BTx-594 in HEK293 cells transiently transfected with BSEP-CaSR-WT, in response to treatment with 5mM or 10mM Ca^{2+}_{e}, to determine optimal conditions to observe ADIS and internalization events. Calcium concentrations are shown above and 'Ca^{2+}_{e} dose' indicates the time when either 5mM or 10mM was added to the cells (n=14). Both 5mM and 10mM doses increased BSEP-CaSR and reduced BTx-594, but this was elevated in cells treated with 10mM Ca^{2+}_{e}. Therefore 10mM was used in subsequent TIRF studies. Treatment of HEK-CaSR cells with Dyngo, and control vehicle (DMSO) did not affect the (C) apoptosis or (D) proliferation rate of cells demonstrating that loss of the sustained pERK1/2 response is not due to Dyngo-mediated changes in cell survival. (E) Western blot analyses of pERK1/2 (top) with densitometric analysis (bottom) following treatment with tunicamycin (+) or DMSO (-). pERK1/2 responses increased over time in tunicamycin-treated cells with the early and sustained responses at 5 minutes and 30 minutes, respectively being present, thereby demonstrating that the sustained signal still arises even though the synthesis pathway is blocked. *$p<0.05$, **$p<0.02$, compared to 0mM response +/- tunicamycin, by 2-way ANOVA analysis. (F) Effect of pertussis toxin (PTx) or ethanol vehicle (veh) on Ca^{2+}_{e}-induced SRE luciferase reporter activity in HEK-CaSR cells. PTx reduced SRE luciferase activity, thereby indicating that $G\alpha_{i/o}$ contributes to the SRE signal. Data shows mean±SEM (n=12); *$p<0.05$ and **$p<0.02$ (2-way ANOVA of veh-treated vs. PTx-treated).

FIGURE S6 Sustained Signal from the CaSR Involves internalization of receptor to Rab5 Endosomes, Related to Figure 6 and 7

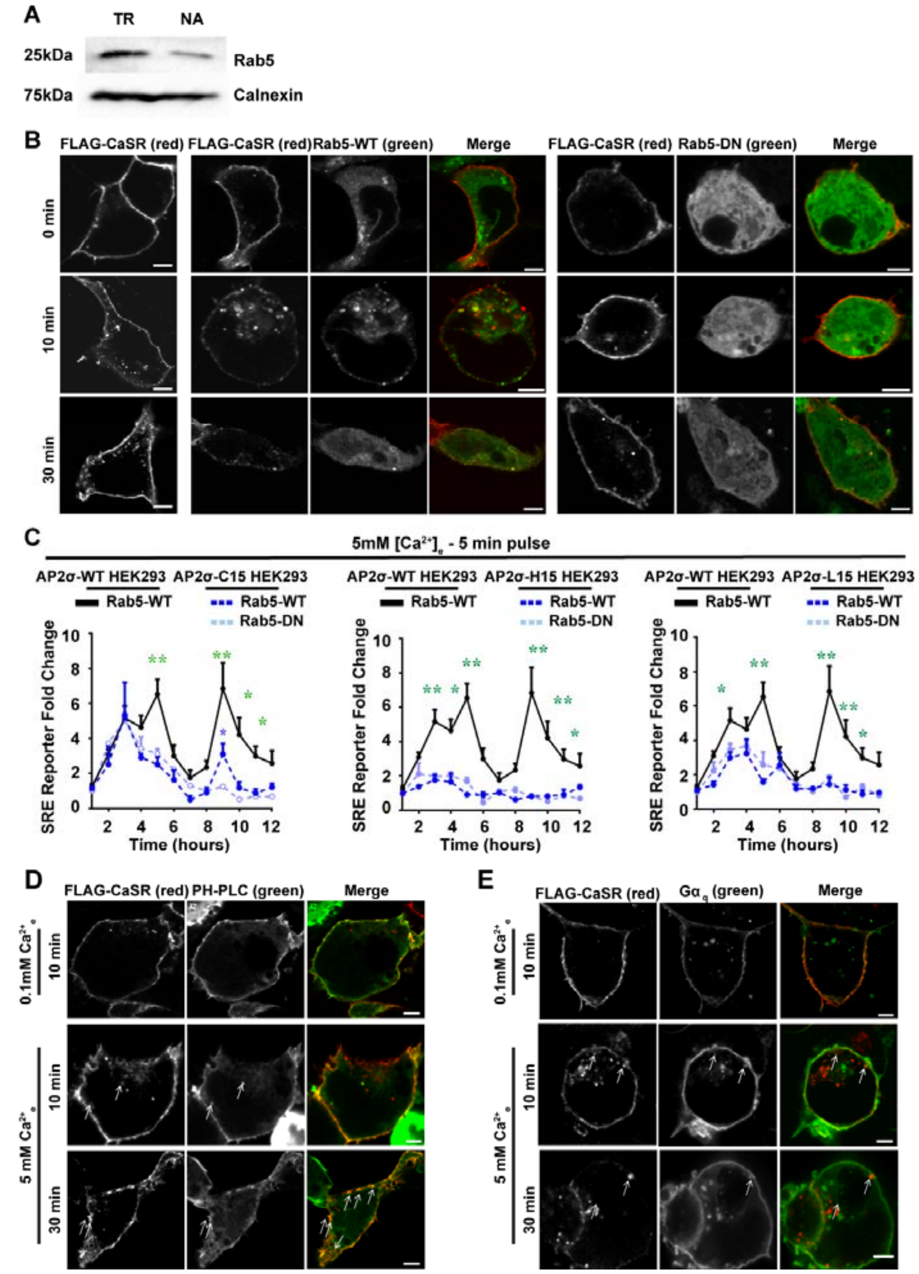

(A) Western blot analysis of cell lysates obtained from native (NA) HEK293 cells for endogenous expression of Rab5, and from HEK293 cells transfected (TR) with mCh-Rab5-WT and overexpressing Rab5. Calnexin was used as a housekeeping protein. (B) Confocal images of HEK293 cells cotransfected with FLAG-CaSR alone (left panel) and with GFP-Rab5-WT or GFP-Rab5-DN. N=8-10 images per construct from 3-4 transfections for B. Scale = 5μm. (C) SRE luciferase reporter response to 5 minute pulses of 5mM Ca^{2+}_e in the presence of Rab5-WT or DN mutant in: AP2σ-WT and AP2σ-mutant cells. Asterisks show AP2σ-WT vs. AP2σ-mutant responses transfected with Rab5-WT (green) and AP2σ-mutant responses in the presence of Rab5-WT vs. Rab5-DN (blue). The Rab5-DN abolished the SRE response in AP2σ-C15 mutant HEK293 cells, thereby indicating that this response requires receptor internalization to Rab5 endosomes. Data are shown as mean+SEM with $*p<0.05$ and $**p<0.02$ (2-way ANOVA). (D-E) Confocal images of cells expressing FLAG-CaSR with either (D) PH-PLC-GFP or (E) $G\alpha_q$-Venus following treatment with 5mM Ca^{2+}_e for 10 and 30 minutes. Merged images reveal partial

colocalization between CaSR and the PLC and $G\alpha_q$ proteins in internalized structures at both time points (indicated by arrows). Scale = 5µm.

Figure S7 CaSR Sustained Signal involves the PLC-DAG-IP₃ pathway but does not require βarrestin proteins, Related to Figure 7

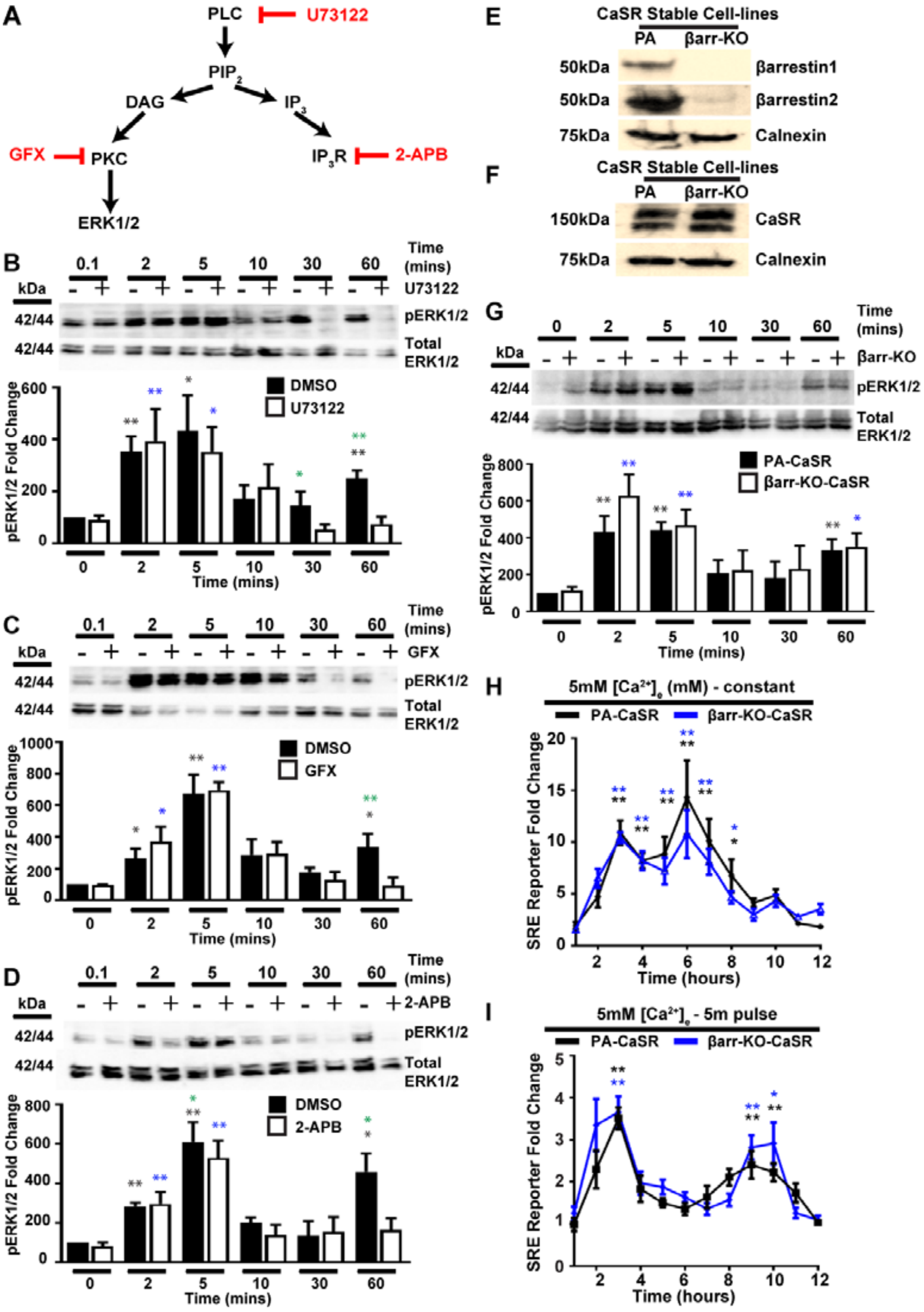

(A) Schematic diagram illustrating the PLC-DAG-IP_3 signaling pathway activated by CaSR, and inhibitors (red) used to examine the role of signal components in sustained signaling. PLC, which can be inhibited by U73122, activates PIP_2 that activates two second messenger proteins DAG and IP_3. PKC, activated by DAG, and the IP_3-receptor (IP_3R), activated by IP_3, are inhibited by the GF-109203X (GFX), and 2-aminoethoxydiphenyl borate (2-APB) compounds, respectively. (B-D) Western blot analyses of pERK1/2 (top) with densitometric analysis (bottom) in HEK-CaSR cells treated with (B) U73122, (C) GFX, or (D) 2-APB. Cells were treated with inhibitor (+) or DMSO (-) and given a 5 minute pulse of 5mM Ca^{2+}_e. The sustained pERK1/2 response was impaired by

U73122, GFX and 2-APB, indicating that the PLC-DAG-IP_3 pathway is involved in the generation of the CaSR sustained signal. *$p<0.05$, **$p<0.02$, by 2-way ANOVA analysis, comparing: responses at 0 mins compared to other time points in DMSO treated cells (black asterisk), responses at 0 mins compared to other time points in U73122, GFX or 2-APB treated cells (blue asterisk), and DMSO vs. U73122, GFX or 2-APB (green asterisk) at each time point. (E) Confirmation of βarrestin-1 and βarrestin-2 protein expression in parental (PA) HEK293 cells stably expressing CaSR, and loss of their expression following deletion in CRISPR-Cas-generated CaSR-HEK293 cell-lines (βarr-KO), by Western blot analysis. (F) Stable overexpression of wild-type CaSR protein in parental HEK293 cells (PA-CaSR) and βarr-KO cells (βarr-KO-CaSR) shown by Western blot analysis. (G) Western blot analyses of pERK1/2 (top) with densitometric analysis (bottom) in the PA-CaSR ((-) in upper panel and PA-CaSR in lower panel) and βarr-KO-CaSR ((+) in upper panel and βarr-KO-CaSR in lower panel) cell-lines following a 5 minute pulse of 5mM Ca^{2+}_e. pERK1/2 responses increased over time with the early and sustained responses being present in both cell-lines, thereby demonstrating that βarrestin-1 and -2 are not required for sustained responses. (H) SRE luciferase reporter responses to treatment with 5mM Ca^{2+}_e over 12 hours in PA-CaSR or βarr-KO-CaSR cells (n=8). (I) SRE luciferase reporter response to 5 minute pulses of 5mM Ca^{2+}_e in PA-CaSR or βarr-KO-CaSR cells (n=8). Data shows mean±SEM. *$p<0.05$, **$p<0.02$. Statistical analyses by 2-way ANOVA, comparing: responses at 0 mins compared to other time points in PA-CaSR cells (black); and responses at 0 mins compared to other time points in βarr-KO-CaSR cells (blue). Densitometric analyses were performed on at least 4 blots from independent lysates.

Supplemental Items

Table S1 List of Oligonucleotide Primer Sequences, Related to Figure S1

REAGENT or RESOURCE	SOURCE	IDENTIFIER
**Genomic Primers**		
AP2S1 Exon 2 Forward	Nesbit *et al*	AGCCCTATCTCCCCTCTGG
AP2S1 Exon 2 Reverse	Nesbit *et al*	GAAGCAAGCAAGCTCAAAGC
**cDNA Primers**		
AP2S1 cDNA Sequencing Primer Forward	Nesbit *et al*	GAAGTCCGCTCTAGCTCTGG
AP2S1 cDNA Sequencing Primer Reverse	Nesbit *et al*	GTTTCAGCACCTTCGTCGG
Forward Primer to generate pcDNA3.1-HisV5 construct	SigmaAldrich	GCCGGATCCATGATCCGCTTTATCCTC
Reverse Primer to generate pcDNA3.1-HisV5 construct	SigmaAldrich	ggccgcGATATCCGTAGAATCGAGACCGAGGAGAGGGTTAGGGATAGGC TTACCTTCGAACCGGCACTCCAGGGACTGTAGCAT
pcDNA3.1 T_7 Primer	SigmaAldrich	TAATACGACTCACTATAGGG
pcDNA3.1 Reverse	SigmaAldrich	TAGAAGGCACAGTCGAGG
Rab5-S34N site-directed mutagenesis Forward	SigmaAldrich	TCCGCTGTTGGCAAAAATAGCCTAGTGCTTCGT
Rab5-S34N site-directed mutagenesis Reverse	SigmaAldrich	ACGAAGCACTAGGCTATTTTTGCCAACAGCGGA
Rab5-Q79L site-directed mutagenesis Forward	SigmaAldrich	TGGGATACAGCTGGTCTTGAACGATACCATAGCC
Rab5-SQ79L site-directed mutagenesis Reverse	SigmaAldrich	GGCTATGGTATCGTTCAAGACCAGCTGTATCCCA
AP2S1 cDNA primer to introduce silent mutations for siRNA sites Forward 1	SigmaAldrich	CGCCTGGCCTTGTGGTATATGCAGTTTGATGAT
AP2S1 cDNA primer to introduce silent mutations for siRNA sites Reverse 1	SigmaAldrich	ATCATCAAACTGCATATACCACAAGGCCAGGCG
AP2S1 cDNA primer to introduce silent mutations for siRNA sites Forward 2	SigmaAldrich	GCCTTGTGGTATATGCAATTCGACGATGATGAG
AP2S1 cDNA primer to introduce silent mutations for siRNA sites Reverse 2	SigmaAldrich	CTCATCATCGTCGAATTGCATATACCACAAGGC
AP2S1 cDNA primer to introduce silent mutations for siRNA sites Forward 3	SigmaAldrich	AGAGACCAGCCAAACCAAAGTACTGAAACAGC
AP2S1 cDNA primer to introduce silent mutations for siRNA sites Reverse 3	SigmaAldrich	GCTGTTTCAGTACTTTGGTTTGGCTGGTCTCT
AP2S1 cDNA primer to introduce silent mutations for siRNA sites Forward 4	SigmaAldrich	CCAGCCAAACCAAAGTACTAAAGCAACTGCTGATG
AP2S1 cDNA primer to introduce silent mutations for siRNA sites Reverse 4	SigmaAldrich	CATCAGCAGTTGCTTTAGTACTTTGGTTTGGCTGG

AP2S1 cDNA primer to introduce silent mutations for siRNA – introduce WT	SigmaAldrich	CGGGCAGGCAAGACGCGCCTGGCCAAATGGTAT
AP2S1 cDNA primer to introduce silent mutations for siRNA – introduce C15	SigmaAldrich	CGGGCAGGCAAGACGTGCCTGGCCAAATGGTAT
AP2S1 cDNA primer to introduce silent mutations for siRNA – introduce H15	SigmaAldrich	CGGGCAGGCAAGACGCACCTGGCCAAATGGTAT
AP2S1 cDNA primer to introduce silent mutations for siRNA – introduce L15	SigmaAldrich	CGGGCAGGCAAGACGCTCCTGGCCAAATGGTAT
**qRT-PCR Primers**		
GAPDH (Hs_GAPDH_1_SG)	Quantitect, Qiagen	Cat#QT00079247
CCND1 (Hs_CCND1_1_SG)	Quantitect, Qiagen	Cat#QT00495285
PGK1 (Hs_PGK1_1_SG)	Quantitect, Qiagen	Cat#QT00013776
CANX (Hs_CANX_1_SG)	Quantitect, Qiagen	Cat#QT00092995
TBP1 (Hs_TBP_1_SG)	Quantitect, Qiagen	Cat#QT00000721
AP2A1 (Hs_AP2A1_1_SG)	Quantitect, Qiagen	Cat#QT00071715
AP2A2 (Hs_AP2A2_1_SG)	Quantitect, Qiagen	Cat#QT00005572
AP2B1 (Hs_AP2B1_1_SG)	Quantitect, Qiagen	Cat#QT01677494
AP2M1 (Hs_AP2M1_1_SG)	Quantitect, Qiagen	Cat#QT00089334
AP2S1 (Hs_AP2S1_1_SG)	Quantitect, Qiagen	Cat#QT01155238
**siRNA**		
siRNA targeting sequence to AP2S1	SantaCruz Biotechnology	sc-97710

Table S2 List of Antibodies, related to Figure S1, S3 and S6

REAGENT or RESOURCE	SOURCE	IDENTIFIER
Mouse monoclonal anti-CaSR (clone 5C10, ADD)	Abcam	Cat#ab19347, RRID: AB_444867
Mouse monoclonal anti-AP2α	BD Transduction Laboratories	Cat#610501, RRID: AB_397867
Mouse monoclonal anti-AP2β	BD Transduction Laboratories	Cat#610381, RRID: AB_397764
Rabbit polyclonal anti-pERK1/2	Cell Signalling Technology	Cat#9101L, RRID: AB_331646
Rabbit polyclonal anti-total ERK1/2 (clone 137F5)	Cell Signalling Technology	Cat#4695S, RRID: 390779
Rabbit polyclonal anti-Calnexin	Millipore	Cat#AB2301, RRID: AB_10948000
Rabbit polyclonal anti-AP2σ	Abcam	Cat#ab173201, RRID:AB_2631096
Rabbit monoclonal anti-AP2μ	Abcam	Cat#ab7995, RRID:AB_1309955
Rabbit polyclonal anti-Rab5	Abcam	Cat#ab18211, RRID:AB_470264
Rabbit polyclonal anti-βarrestin-1	Genetex	Cat#30065, RRID:AB_2715557
Mouse monoclonal (H-9) anti-βarrestin-2	SantaCruz Biotechnology	Cat#sc-13140, RRID:AB_626701
Phalloidin-594	ThermoFisher Scientific	Cat#A12381 RRID:AB_2315633
Donkey anti-rabbit Alexa Fluor 488	Molecular Probes	Cat# A-11094, RRID:AB_221544
Donkey anti-rabbit Alexa Fluor 555	Molecular Probes	Cat# ab150074, RRID:AB_2636997
Transferrin-594	Molecular Probes	Cat#T13343, RRID:AB_2716742
Goat anti-rabbit IgG-HRP conjugate	Biorad	Cat#1706515, RRID: AB_11125142
Donkey anti-mouse IgG-HRP conjugate polyclonal	SantaCruz Biotechnology	Cat#sc-2318, RRID: AB_641171

Supplemental Experimental Procedures

Cell Culture

HEK293 cells were cultured in DMEM Glutamax media (Gibco) with 10% fetal bovine serum (FBS) (Gibco), and those stably expressing either CaSR or AP2σ proteins were supplemented with 400μg geneticin (Gibco). HEK293 cells with deletion of *GNAQ*, *GNA11*, *GNA12*, and *GNA13* genes, encoding $G\alpha_q$, $G\alpha_{11}$, $G\alpha_{12}$ and $G\alpha_{13}$, respectively; or combined deletions of *ARRB1 and ARRB2* encoding βarrestin-1 and βarrestin-2, respectively, were generated by gene-editing using CRISPR-Cas (Devost et al., 2017). All transfections were performed with Lipofectamine 2000 (Invitrogen). AP2σ stable cells were pre-treated with 100nM AP2σ siRNA (SantaCruz Biotechnology) prior to performing functional assays. For functional studies, AP2σ cells were transiently transfected with pEGFP-CaSR-WT (Pearce et al., 1996). All studies were performed in plates pre-treated with poly-L-lysine (Sigma).

Human Lymphoblastoid Cells from FHH3 Patients

Informed consent was obtained from individuals using protocols approved by local and national ethics committees, London, UK (MREC/02/2/93). Epstein-Barr virus (EBV) transformed lymphoblastoid cells were established using methods previously described (Parkinson and Thakker, 1992), using leukocytes, from four affected and four unaffected members of the FHH3 kindred with AP2σ-C15 mutations (Nesbit et al., 2013). Lymphoblastoid cells were maintained in RPMI-1640 media supplemented with 10% FBS and penicillin/streptomycin (Gibco).

Construction of Stable Cell-lines

HEK293 cell-lines stably expressing full-length CaSR (HEK-CaSR) have been previously described (Nesbit et al., 2013). HEK293 cells with deletion of βarrestin-1 and βarrestin-2 (βarr-KO) that stably express the CaSR were generated using a pcDNA3.1 construct (Invitrogen) containing full-length CaSR cDNA, as previously described (Nesbit et al., 2013). HEK293 cell-lines stably expressing either AP2σ-wild-type (WT) or AP2σ-mutant proteins (C15, H15 or L15) were generated using a pcDNA3.1 construct containing full-length AP2σ cDNA with a C-terminal V5 epitope. Silent mutations were introduced to render the construct insensitive to AP2σ-targeted siRNA (SantaCruz Biotechnology). Oligonucleotide sequences used for construct generation and introduction of silent mutations were purchased from Sigma (Table S1). pcDNA3.1-AP2σ-V5 constructs were transfected into HEK293 cells and cultured in geneticin selection media. Individual pcDNA3.1-AP2σ-V5 positive clones were picked and subcultured in fresh selection media. AP2σ expression was assessed by Western Blot analysis. HEK293 cells were chosen as a model cell system to examine trafficking of the CaSR for the following reasons: i) HEK293 have been previously used and established as a model to assess CaSR function; ii) HEK293 cells are used as appropriate cultures of parathyroid and CaSR-expressing kidney cell-lines are not available; iii) the ADIS phenomenon of CaSR was first described in HEK293 cells (Grant et al., 2011); iv) we have previously demonstrated AP2σ-mutant protein effects on intracellular calcium using HEK293 cells (Nesbit et al., 2013); and, v) HEK293 cells transfected with CaSR respond to calcium in a concentration-dependent manner and utilize previously characterized pathways of intracellular calcium release, MAPK activation and cAMP signaling (Conigrave and Ward, 2013). Mutations within the constructs were introduced by site-directed mutagenesis using the Quikchange Lightning XL or Multi kits (Agilent Technologies).

Confirmation of Mutations and DNA Sequencing

The DNA sequence abnormalities in the lymphoblastoid cell-lines were confirmed by using extracted DNA (Gentra Puregene Blood Kit (Qiagen)) and PCR amplification of *AP2S1* exon 2, followed by restriction endonuclease analysis utilizing *HhaI* (New England Biolabs), as previously described (Nesbit et al., 2013). Presence of mutations within constructs were verified using dideoxynucleotide sequencing with the BigDye Terminator v3.1 Cycle Sequencing Kit (Life Technologies) and an automated detection system (ABI3730 Automated capillary sequencer; Applied Biosystems) (Gorvin et al., 2017a; Newey et al., 2013). The oligonucleotide sequences that were used are listed in Table S1.

Western Blot Analysis

The antibodies used for Western blot analysis are listed in Table S2. For studies of protein expression, cells were lysed in NP40 lysis buffer (50mM Tris HCl pH7.4, 1mM EDTA, 150mM NaCl, protease inhibitors) (Newey et al., 2013), lysates were resuspended in Laemmli buffer, boiled and separated on 6% and 12% sodium-dodecyl sulphate (SDS) polyacrylamide gel electrophoresis gels. Following transfer to polyvinylidene difluoride membrane (Amersham), blots were blocked in 5% BSA/TBS-t (pERK1/2 studies) or marvel/TBS-t, then probed with the primary and secondary antibodies. Blots were visualized using the Immuno-Star WesternC kit (BioRad) on a BioRad Chemidoc XRS+ system (Newey et al., 2013). Following development, blots were stripped with Restore Western blot stripping buffer (Thermo Scientific), blocked in marvel/TBS-t and reprobed with primary

antibodies. For studies of AP2 subunit expression, cells were probed with the AP2σ antibody, followed by calnexin (used as a housekeeping protein), AP2μ, AP2β, then AP2α, with stripping of blots between each antibody. For all other Western blots, calnexin was used as the housekeeping protein after initial probing with the gene of interest.

For sustained signaling studies, cells were stimulated with 5mM $CaCl_2$ for 5 minutes, followed by incubation in media containing 0mM $CaCl_2$ for 0-60 minutes. For studies with 30μM Dyngo-4a (Abcam) (Jean-Alphonse et al., 2014), cells were pre-incubated for 30 minutes. For studies with 5μM U73122 (Sigma), 1μM GF-109203X (Sigma), 100 μM 2-aminoethoxydiphenyl borate (2-APB) (Sigma), or 5μg/mL tunicamycin (Sigma) (Avlani et al., 2013; Grant et al., 2011; Luo et al., 2001), compounds were added to the media and cells were incubated after calcium stimulation. After analysis for pERK1/2, blots were stripped and reprobed with an anti-total ERK1/2 antibody (Gorvin et al., 2017a). For studies of Rab5 contribution to sustained signaling, 100ng/ml mCh-Rab5-WT (Addgene plasmid ♯49201), or mCh-Rab5-dominant negative (DN, S34N) or –constitutively active (CA, Q79L) (generated by site-directed mutagenesis using oligonucleotides listed in Table S1) were transfected 48-hours before Western blot analysis. Densitometry analysis was performed using ImageJ and statistical analyses were performed by 2-way ANOVA using Graphpad Prism 6.

Quantitative RT-PCR (qRT-PCR) Analysis

First-strand cDNA was generated using the Quantitect reverse transcription kit (Qiagen) from 1μg total RNA from each lymphoblastoid cell-line extracted using the MirVana (Ambion) kit (Gorvin et al., 2013). All qRT-PCR test samples were normalized to levels of the geometric mean of five reference genes, *GAPDH*, *CCND1*, *PGK1*, *CANX* and *TBP1.* Primers were obtained from Quantitect (Qiagen). Threshold cycle (CT) values were obtained from the start of the log phase on Rotorgene Q Series Software, and CT values analyzed in Microsoft Excel 2011 (Gorvin et al., 2013)and graphs generated using GraphPad Prism 6. Studies were performed in 4 biological replicates each in quadruplicate. Data was analyzed by Student's t-test.

Uptake Assays

Transferrin uptake assays were performed in native HEK293 cells and AP2σ-WT and AP2σ-mutant HEK293 cells. Transferrin assays were performed as previously described (Gorvin et al., 2013). Cells were seeded in 24-well plates, transfected with pEGFP-CaSR-WT to yield AP2σ-WT/CaSR-WT or AP2σ-mutant/CaSR-WT HEK293 cells, and incubated for 24 hours. Cells were incubated in serum-free media (SFM) prior to treatment with 5μg/mL transferrin conjugated to Alexa Fluor 594 (Molecular Probes) for 0 or 10 minutes. Following incubation, cells were washed in PBS and lysed in NP40 buffer. Fluorescence was measured using a CytoFluor microplate reader (PerSeptive Biosystems) at 580nm excitation and 615nm emission wavelengths. Cell surface expression of β2AR was performed in AP2σ-WT and AP2σ-mutant HEK293 cells using an ELISA-based assay and a β2AR-FLAG construct (Jean-Alphonse et al., 2014) using methods adapted from previously reported studies (Grant et al., 2011; Nesbit et al., 2013). Surface expression was assessed in cells treated with 0μM or 10 μM isoproterenol for 30 minutes. Fluorescence was measured using the PHERAstar FS microplate reader (BMG Labtech). Total fluorescence was normalized to total cellular protein measured by Coomassie Bradford Assay (Pierce). Data was normalized to uptake at 0mins in AP2σ-WT cells. Statistical analyses were performed using 2-way ANOVA (Microsoft Excel 2011 and GraphPad Prism 6).

Single-Cell Ca^{2+} Microfluorimetry

Single-cell microfluorimetry experiments were performed in AP2σ-WT or AP2σ-mutant HEK293 cells. Cells were transiently transfected with pEGFP-CaSR-WT. Cells were plated on coverslips 12 hours prior to imaging and incubated in extracellular solution composed of: 140mM NaCl, 5mM KCl, 1.2mM $MgCl_2$, 1mM NaH_2PO_4, 5mM $NaHCO_3$, 10mM HEPES, 10mM glucose, pH7.4. The appropriate $CaCl_2$ concentration was added and adjusted to maintain osmolality to 324.4 mOsm/L. Experiments were performed at 37°C. Fura-2 dye (Life Technologies) was dissolved in DMSO containing 0.03% F127-Pluronic (Sigma). Cells were loaded with 4μM Fura-2 (Molecular Probes) in extracellular solution for 30 minutes at room temperature. Imaging experiments were performed on a Zeiss Axioscope FS2 wide-field microscope with a 40x/1.3 objective. Cells were continuously perfused with extracellular bath solutions, with concentrations of $CaCl_2$ being increased every 2.5 minutes. Cells were imaged initially for the presence of DsRed2 fluorescence, followed by live capture of Fura-2 using 340/380nm excitation and 510nm emission, acquired every 30 seconds using a Hamamatsu OrcaR2 CCD camera controlled by μManager software (Edelstein et al., 2014) and analyzed using ImageJ (NIH) (Schneider et al., 2012). Fura-2 ratios were calculated in Microsoft Excel 2011 and graphs generated using GraphPad Prism 6. Nonlinear regression of concentration-response curves was performed with GraphPad using the normalized response at each $[Ca^{2+}]_e$ for each separate experiment for the determination of EC_{50} (i.e. $[Ca^{2+}]_e$ required for 50% of the maximal response). The maximal signaling response was measured as a fold-change of the peak transient Ca^{2+}_i response to each $[Ca^{2+}]_e$. The EC_{50} values were compared using the *F*-test and the maximal signaling

responses assessed using the Mann-Whitney *U* test. The number of oscillating cells at each $[Ca^{2+}]_e$ was calculated as a percentage of that in WT cells. Statistical analyses were performed using the χ^2 test.

AlphaScreen Assays

AlphaScreen assays (PerkinElmer) were performed in AP2σ-WT or AP2σ-mutant HEK293 cells, HEK-CaSR, HEK293 or lymphoblastoid cells. Assays were performed in 48-well plates and AP2σ-WT and AP2σ-mutant cells transiently transfected with 200ng pEGFP-CaSR 48-hours prior to performance of assays. For pERK1/2 assays, cells were incubated in SFM 12 hours prior to 5 minute treatment with 0-15mM $CaCl_2$. Cells were then lysed in Surefire lysis buffer and pERK1/2 and total ERK1/2 assays performed as previously described (Gorvin et al., 2017a). For cAMP assays involving AP2σ and CaSR, cells were treated with 10μM forskolin for 30 minutes prior to $CaCl_2$ treatment in stimulation buffer (1x Hanks Buffered Saline Solution, 0.1% BSA, 0.1% 3-isobutyl-1-methylxanthine (IBMX), 0.5mM HEPES) plus 0-10mM $CaCl_2$. For studies with PTx, cells were pre-treated with 300ng/mL PTx or vehicle (ethanol) for 6 hours. For studies with UBO-QIC, cells were pre-treated with 1μM UBO-QIC or vehicle (DMSO) for 2 hours. For inhibitor studies, cells were pre-treated with: 15μM gallein or vehicle (DMSO) for 15 minutes (Grant et al., 2011). Cells were incubated with 0-10mM $CaCl_2$ for 15 minutes, then lysed in a HEPES-based solution and AlphaScreen assays performed. For studies of β2AR, cells were transiently transfected with β2AR-FLAG and cells were incubated with 10μM isoproterenol (Sigma) for 30 minutes, then lysed and AlphaScreen assays performed. The fluorescence signal in both assays was measured using the PHERAstar FS microplate reader (BMG Labtech) (Newey et al., 2013). A minimum of 4 independent biological replicates were used. Statistical analysis was performed by 2-way ANOVA with Tukey's multiple-comparisons test using Microsoft Excel 2011, and Graphpad Prism 6.

IP_1 Assays

IP_1 assays were performed in AP2σ-WT and AP2σ-mutant HEK293 cells. IP_1 assays were performed in 24-well plates and cells transiently transfected with 200ng pEGFP-CaSR 48-hours prior to performance of assays. At 24-hours prior to experiments, cells were re-plated in a 384-well plate, and 12-hours later, media changed to serum-free media. IP_1 homogenous time-resolved fluorescence (HTRF) assays (Cisbio) were performed according to manufacturer's instructions, and as previously described (Zhang et al., 2014). Cells were incubated for 5 minutes with stimulation buffer containing a single dose of $CaCl_2$ (between 0.1-10mM), followed by lysis in the supplied lysis buffer. Plates were read on a PHERAStar FS microplate reader one hour later (BMG Labtech).

Luciferase Reporter Assays

Luciferase reporter assays were performed in AP2σ-WT and AP2σ-mutant HEK293 cells, CRISPR-Cas generated HEK293 $G\alpha_{q/11}$, $G\alpha_{12/13}$ and $G\alpha_{q/11/12/13}$ cells, βarr-KO cells or HEK-CaSR cells. Cells were plated in 24-well plates and transiently transfected with 100ng/ml luciferase reporter constructs (either pGL4-NFAT, pGL4-SRE, or pGL4-SRF) and 10ng/ml pRL. For studies in AP2σ-WT and mutant cells, and CRISPR-Cas cells, 100ng/ml pEGFP-CaSR-WT was transfected simultaneously with luciferase reporter and pRL constructs. For studies of Rab5, 100ng/ml mCh-Rab5-WT or Rab5-mutant (DN or –CA) were transfected simultaneously with luciferase reporter and pRL constructs. For all studies, cells were treated with SFM 36 hours after transfection. On the day of the experiment, cells were treated with SFM containing 0-10mM $CaCl_2$ for 4 hours (for concentration-response studies), or, for sustained signaling studies, with SFM containing one of four additions: i) 0mM $CaCl_2$; ii) 5mM $CaCl_2$ for the duration of the experiment (constant); iii) 5 minute pulse of 5mM $CaCl_2$ followed by 0mM $CaCl_2$ with vehicle for the duration of the experiment; or iv) 5 minute pulse of 5mM $CaCl_2$ followed by 0mM $CaCl_2$ with 30μM Dyngo-4a for the duration of the experiment. DMSO was used as the vehicle. Cells were lysed at the end of the 4-hour incubation period for concentration-response studies, or one plate each hour for 12 hours for sustained signaling studies, and luciferase assays performed using Dual-Glo Luciferase (Promega), on a Veritas Luminometer (Promega), as previously described (Gorvin et al., 2017a). Cells were pre-incubated with 1μM UBO-QIC or DMSO for 2 hours, to study $G\alpha_{q/11}$, or for $G\alpha_{i/o}$, cells were treated with 10μM forskolin (MP Biomedicals) and 300ng/ml pertussis toxin (PTx) (Sigma) or vehicle (ethanol diluent) for 6 hours (Avlani et al., 2013), prior to SRE measurement where appropriate. Luciferase:renilla ratios were expressed as fold changes relative to responses at 0mM $CaCl_2$ responses. All assay conditions were performed in 4-8 biological replicates. Statistical analyses were performed using 2-way ANOVA (Microsoft Excel 2011 and GraphPad Prism 6).

Membrane Ruffling

Membrane ruffling studies were performed in AP2σ-WT and AP2σ-mutant HEK293 cells and HEK293 native and CRISPR-Cas $G\alpha_{12/13}$ cells. Cells were plated in 6-well plates on coverslips and transiently transfected with pEGFP-CaSR-WT. Following 24 hour incubation, serum-free media was applied and cells incubated overnight. Methods were adapted from protocols previously described (Bouschet et al., 2007; Davey et al., 2012). Cells were treated with serum-free media containing either 0, 5 or 10mM $CaCl_2$ for 5 minutes, followed by fixing in 4% paraformaldehyde/PBS, permeabilization with triton-X100/PBS, and staining with 4U/mL Phalloidin conjugated

to Alexa Fluor 594 (Molecular Probes). Coverslips were imaged on a Nikon Eclipse E400 wide-field microscope. Cells were classified as ruffled if they fulfilled three criteria: colocalization of CaSR and phalloidin-594, which stains actin; presence of at least two ruffles; and cells were singlets or if in clusters had at least two sides exposed. Up to 10 images of each coverslip were taken, and 82-313 cells were imaged over 15-45 independent transfections. Statistical analyses were performed using χ^2 tests (Microsoft Excel 2011 and GraphPad Prism 6).

Total Internal Reflection Fluorescence Microscopy (TIRF-M)

TIRF-M studies were performed in AP2σ-WT or AP2σ-mutant HEK293, and CRISPR-Cas $G\alpha_{q/11}$ knockout cells. Cells were transfected with BSEP-CaSR (Grant et al., 2011) 24-hours prior to recordings. For clathrin studies, DsRed2-Clathrin was transfected simultaneously with pEGFP-CaSR-WT. Methods for TIRF-M were adapted from previous studies (Grant et al., 2011; Hoppa et al., 2009). Images were obtained with a customized Olympus IX-81 TIRF microscope equipped with a 60×/1.45 Apo lens (Olympus). The 488nm line of an argon ion laser (Melles Griot) was used to excite SEP-CaSR, and a 561nm line of a steady-state diode laser was used to excite BTx-594 or DsRed-Clathrin. Two excitation ports with separate focal pathways were used to make independent adjustments of each laser. The emission pathway for both fluorescent reporters was imaged simultaneously with an image splitter (Dual View, Optical Insights) generating side-by-side images of both emission wavelengths on the chip of an electron multiplying charge-coupled device (EMCCD) camera (Cascade II 512B, Roper Scientific). Alignment of the two emission channels was corrected on a daily basis by imaging 200nm tetraspeck beads (Molecular Probes). Extracellular medium for imaging consisted of 140mM NaCl, 5mM KCl, 0.55 $MgCl_2$, 10mM HEPES and 10mM D-glucose, pH 7.4. The appropriate $CaCl_2$ concentration was added and adjusted with NaCl. All solutions were osmotically balanced. Experiments were performed at 37°C. To monitor CaSR internalization, cells were incubated with 5μg/mL BTx-594 for 3 minutes prior to imaging. Experiments were performed with continuous imaging of cells perfused with basal 0.1mM $CaCl_2$ imaging solution for 1 minute, followed by addition of 10mM $CaCl_2$ imaging solution for 10 minutes. The following 9 minutes were conducted in basal solution. Solutions took 45 seconds from switching the perfusion to reaching the chamber. These methods were chosen following initial experiments in cells using 5mM or 10mM $Ca^{2+}{}_e$ as the stimulant, which demonstrated more robust responses with 10mM $Ca^{2+}{}_e$. Images were captured at 10 frames/sec in BSEP studies. Images were acquired using Cell^R software (Olympus) and analyzed with ImageJ. Fluorescence intensity was measured in each frame of captured movies at both emission wavelengths, and green fluorescence intensity normalized to red fluorescence to acquire total surface CaSR.

For CaSR and clathrin studies, one frame of BSEP-CaSR and DsRed-Clathrin was imaged every 3 seconds for 10 minutes. Cells were initially bathed in basal $CaCl_2$ solution followed by 10mM solution for 9 minutes. CaSR and clathrin positive vesicles were assessed for colocalisation by measuring fluorescence intensity at each emission wavelength. CaSR-clathrin puncta were analyzed if they adhered to the following criteria adapted from previous studies (Mattheyses et al., 2011): 1) CaSR and clathrin colocalized for at least 4 frames (12 secs); 2) Clathrin puncta are diffraction limited; 3) spots disappear simultaneously before the end of the imaging period; 4) vesicle does not merge with other regions of fluorescence. Vesicles with no, or limited movement, during the duration of the movie were classified as non-motile, while those that moved from their original position over a span of two or more frames were classified as motile vesicles (Mukhopadhyay et al., 2011). Vesicles were tracked using ImageJ.

Data from individual cells was averaged over 30-44 independent experiments for AP2σ studies, 15-25 experiments for CRISPR-Cas studies and 95-200 vesicles in 14-16 cells for clathrin studies and analyzed in Microsoft Excel 2011. Graphs were generated in GraphPad Prism 6.

Confocal Microscopy

Confocal imaging was performed in native HEK293 cells using methods adapted from previous studies (Bouschet et al., 2007; Hanyaloglu et al., 2005). Cells were transfected with FLAG-CaSR, GFP-Rab5-WT, GFP-Rab5-DN, PH-PLC-GFP or $G\alpha_q$-Venus. Prior to all studies, cells were incubated in low calcium Earle's solution (140mM NaCl, 5mM KCl, 0.5mM $CaCl_2$, 0.8mM $MgCl_2$, 25mM HEPES pH 7.4, 1M glucose) for 30 minutes. To label receptors, cells were 'fed' with mouse anti-FLAG antibody (Sigma) for 15 minutes prior to ligand stimulation. Cells were fixed in 4% paraformaldehyde/PBS (Sigma) for 20 minutes, permeabilised with 0.2% triton-X100/PBS (Thermo Scientific), followed by immunostaining with secondary antibodies Alexa Fluor 488 or Alexa Fluor 555 (both Molecular Probes). For FLAG-CaSR, cells were incubated with and without 5mM $Ca^{2+}{}_e$ for 10 or 30 minutes. Cells were mounted in ProLong Gold Antifade Mountant (Life Technologies) or Fluoromount-G (Thermo Fisher). Images were captured using a confocal, laser-scanning microscope (Leica SP5) with a Plan-Achromat ×63/1.4 oil DIC objective. Images were analyzed using Leica LAS AF image acquisition software. All subsequent raw image files were analyzed using ImageJ or LAS AF Lite (Leica) to measure level of colocalization.

Pearson's correlation coefficient was calculated for at least 3 regions of interest per cell using the ImageJ plugin JACoP.

Apoptosis and Proliferation Assays

HEK-CaSR cells were plated in 96-well plates and apoptosis and proliferation assessed every 4 hours using the Caspase-Glo 3/7 and CellTiter Blue kits, respectively (Promega). Caspase-Glo 3/7 was measured on a Veritas luminometer, and CellTiter Blue on a CytoFluor microplate reader (PerSeptive Biosystems).

Quantification and Statistical Analyses

Statistical analyses are indicated in the legends of each figure, including the definitions of error bars (e.g. standard error, 95% confidence intervals) and the number of experimental replicates denoted by n. Two-tailed unpaired t test, 2-way ANOVA, Mann-Whitney U-test, χ^2-test, Pearson's correlation coefficient and the F-test were used to calculate statistical significance using Graphpad Prism 6 or ImageJ software. A p value of <0.05 was considered statistically significant. Statistical tests used for each experiment are indicated in the relevant methods section.

Supplemental References

Boucrot, E., Saffarian, S., Zhang, R., and Kirchhausen, T. (2010). Roles of AP-2 in clathrin-mediated endocytosis. PloS one 5, e10597.

Edelstein, A.D., Tsuchida, M.A., Amodaj, N., Pinkard, H., Vale, R.D., and Stuurman, N. (2014). Advanced methods of microscope control using muManager software. Journal of biological methods 1.

Gorvin, C.M., Rogers, A., Stewart, M., Paudyal, A., Hough, T.A., Teboul, L., Wells, S., Brown, S.D.M., Cox, R.D., and Thakker, R.V. (2017b). N-ethyl-N-nitrosourea Induced Adaptor Protein 2 Sigma Subunit 1 (Ap2s1) Mutations Establish Ap2s1 Loss-of-function Mice. JBMR Plus.

Hoppa, M.B., Collins, S., Ramracheya, R., Hodson, L., Amisten, S., Zhang, Q., Johnson, P., Ashcroft, F.M., and Rorsman, P. (2009). Chronic palmitate exposure inhibits insulin secretion by dissociation of Ca(2+) channels from secretory granules. Cell metabolism 10, 455-465.

Lin, K., Wang, D., and Sadee, W. (2002). Serum response factor activation by muscarinic receptors via RhoA. Novel pathway specific to M1 subtype involving calmodulin, calcineurin, and Pyk2. The Journal of biological chemistry 277, 40789-40798.

Luo, D., Broad, L.M., Bird, G.S., and Putney, J.W., Jr. (2001). Signaling pathways underlying muscarinic receptor-induced [Ca2+]i oscillations in HEK293 cells. The Journal of biological chemistry 276, 5613-5621.

Mattheyses, A.L., Atkinson, C.E., and Simon, S.M. (2011). Imaging single endocytic events reveals diversity in clathrin, dynamin and vesicle dynamics. Traffic 12, 1394-1406.

McMurtry, C.T., Schranck, F.W., Walkenhorst, D.A., Murphy, W.A., Kocher, D.B., Teitelbaum, S.L., Rupich, R.C., and Whyte, M.P. (1992). Significant developmental elevation in serum parathyroid hormone levels in a large kindred with familial benign (hypocalciuric) hypercalcemia. The American journal of medicine 93, 247-258.

Mitsunari, T., Nakatsu, F., Shioda, N., Love, P.E., Grinberg, A., Bonifacino, J.S., and Ohno, H. (2005). Clathrin adaptor AP-2 is essential for early embryonal development. Molecular and cellular biology 25, 9318-9323.

Mukhopadhyay, A., Nieves, E., Che, F.Y., Wang, J., Jin, L., Murray, J.W., Gordon, K., Angeletti, R.H., and Wolkoff, A.W. (2011). Proteomic analysis of endocytic vesicles: Rab1a regulates motility of early endocytic vesicles. Journal of cell science 124, 765-775.

Pearce, S.H., Bai, M., Quinn, S.J., Kifor, O., Brown, E.M., and Thakker, R.V. (1996). Functional characterization of calcium-sensing receptor mutations expressed in human embryonic kidney cells. J Clin Invest 98, 1860-1866.

Schneider, C.A., Rasband, W.S., and Eliceiri, K.W. (2012). NIH Image to ImageJ: 25 years of image analysis. Nature methods 9, 671-675.

Zhang, C., Mulpuri, N., Hannan, F.M., Nesbit, M.A., Thakker, R.V., Hamelberg, D., Brown, E.M., and Yang, J.J. (2014). Role of Ca2+ and L-Phe in regulating functional cooperativity of disease-associated "toggle" calcium-sensing receptor mutations. PloS one 9, e113622.

Zhang, W., and Liu, H.T. (2002). MAPK signal pathways in the regulation of cell proliferation in mammalian cells. Cell Res 12, 9-18.
