# Supplementary material for: AP2σ Mutations Impair Calcium-Sensing Receptor Trafficking and Signaling, and Show an Endosomal Pathway to Spatially Direct G-Protein Selectivity
Source: Cell Rep. 2018 Jan 28;22(4):1054–66. doi: 10.1016/j.celrep.2017.12.089 (PMC5792449; doi:10.1016/j.celrep.2017.12.089)
Supplement: Document S2. Article plus Supplemental Information [file mmc2.pdf]

Cell Reports

Article

AP2σ Mutations Impair Calcium-Sensing Receptor Trafficking and Signaling, and Show an Endosomal Pathway to Spatially Direct G-Protein Selectivity

Graphical Abstract

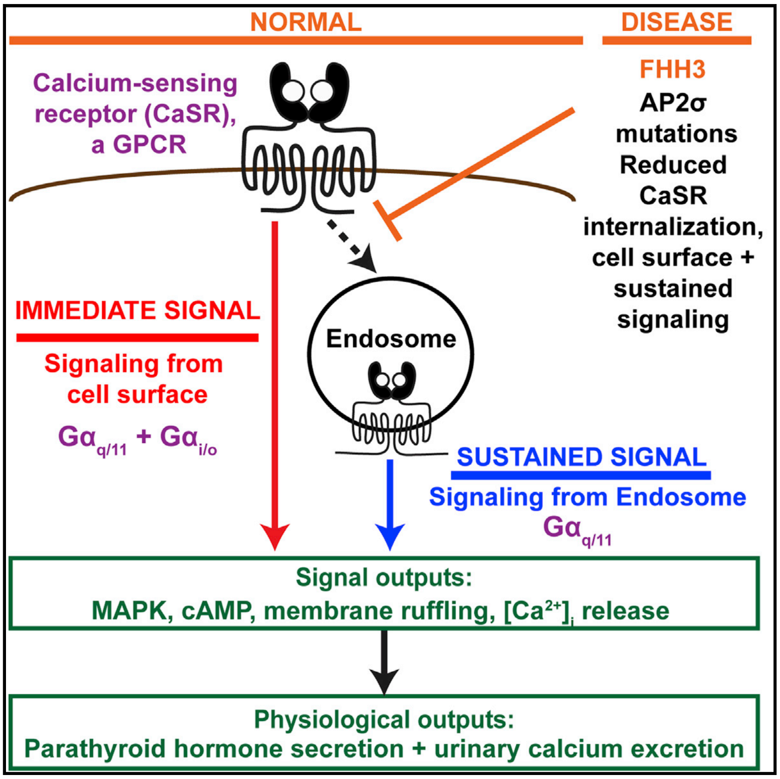

Highlights

- Disease-causing AP2σ mutants impair $G\alpha_{q/11}$ and $G\alpha_{i/o}$ signaling by CaSR, a class C GPCR
- AP2σ mutants impair trafficking of the CaSR
- The CaSR can signal by a sustained endosomal pathway
- CaSR differentially uses $G\alpha_{q/11}$ and $G\alpha_{i/o}$ for cell-surface and endosomal signaling

Authors

Caroline M. Gorvin, Angela Rogers, Benoit Hastoy, ..., Aylin C. Hanyaloglu, Gerda E. Breitwieser, Rajesh V. Thakker

Correspondence

rajesh.thakker@ndm.ox.ac.uk

In Brief

Gorvin et al. show that the class C GPCR calcium-sensing receptor (CaSR) mediates signaling from plasma membranes using $G\alpha_{q/11}$ and $G\alpha_{i/o}$ and from endosomes by using only $G\alpha_{q/11}$. Adaptor protein-2 σ subunit (AP2σ) mutations impair CaSR internalization, leading to reduced sustained endosomal signaling and hypercalcemia in humans.

Gorvin et al., 2018, Cell Reports *22*, 1054–1066
January 23, 2018 © 2017 The Author(s).
https://doi.org/10.1016/j.celrep.2017.12.089

CellPress

OPEN ACCESS
CellPress

Cell Reports
Article

AP2σ Mutations Impair Calcium-Sensing Receptor Trafficking and Signaling, and Show an Endosomal Pathway to Spatially Direct G-Protein Selectivity

Caroline M. Gorvin,[1,8,9] Angela Rogers,[1] Benoit Hastoy,[2] Andrei I. Tarasov,[2] Morten Frost,[1] Silvia Sposini,[3] Asuka Inoue,[4,5] Michael P. Whyte,[6] Patrik Rorsman,[2] Aylin C. Hanyaloglu,[3] Gerda E. Breitwieser,[7] and Rajesh V. Thakker[1,10,*]
[1]Academic Endocrine Unit, Radcliffe Department of Medicine, University of Oxford, Oxford, UK
[2]Diabetes Research Laboratory, Radcliffe Department of Medicine, University of Oxford, Oxford, UK
[3]Institute of Reproductive and Developmental Biology, Faculty of Medicine, Imperial College London, London, UK
[4]Laboratory of Molecular and Cellular Biochemistry, Tohoku University, Sendai, Japan
[5]Japan Science and Technology (JST) Agency, Precursory Research for Embryonic Science and Technology (PRESTO), Kawaguchi, Japan
[6]Center for Metabolic Bone Disease and Molecular Research, Shriners Hospitals for Children, St. Louis, MO, USA
[7]Geisinger Clinic, Weis Center for Research, Department of Functional and Molecular Genomics, Danville, PA, USA
[8]Institute of Metabolism and Systems Research, University of Birmingham, Birmingham, UK
[9]Centre for Endocrinology, Diabetes and Metabolism (CEDAM), Birmingham Health Partners, Birmingham, UK
[10]Lead Contact
*Correspondence: rajesh.thakker@ndm.ox.ac.uk
https://doi.org/10.1016/j.celrep.2017.12.089

SUMMARY

Spatial control of G-protein-coupled receptor (GPCR) signaling, which is used by cells to translate complex information into distinct downstream responses, is achieved by using plasma membrane (PM) and endocytic-derived signaling pathways. The roles of the endomembrane in regulating such pleiotropic signaling via multiple G-protein pathways remain unknown. Here, we investigated the effects of disease-causing mutations of the adaptor protein-2 σ subunit (AP2σ) on signaling by the class C GPCR calcium-sensing receptor (CaSR). These AP2σ mutations increase CaSR PM expression yet paradoxically reduce CaSR signaling. Hypercalcemia-associated AP2σ mutations reduced CaSR signaling via $G\alpha_{q/11}$ and $G\alpha_{i/o}$ pathways. The mutations also delayed CaSR internalization due to prolonged residency time of CaSR in clathrin structures that impaired or abolished endosomal signaling, which was predominantly mediated by $G\alpha_{q/11}$. Thus, compartmental bias for CaSR-mediated $G\alpha_{q/11}$ endomembrane signaling provides a mechanistic basis for multidimensional GPCR signaling.

INTRODUCTION

The G-protein-coupled receptor (GPCR) family is the largest family of signaling receptors, and GPCRs contribute significantly to fundamental cellular functions. The archetypal model of GPCR signaling has evolved from a single, cell-surface receptor activating a specific heterotrimeric G-protein pathway to a complex network in which receptors can activate multiple pathways, exhibit signal crosstalk, and display functional selectivity (Rosenbaum et al., 2009). This is illustrated by the calcium-sensing receptor (CaSR), a class C GPCR that is widely expressed and has calcitropic roles, i.e., regulation of extracellular calcium ($Ca^{2+}{}_e$) by the parathyroids, kidneys, and bone, and non-calcitropic roles such as inflammation, bronchoconstriction, wound healing, gastro-pancreatic hormone secretion, hypertension, and glucose metabolism (Hofer et al., 2000; Rossol et al., 2012; Yarova et al., 2015; Zietek and Daniel, 2015). Thus, the CaSR, which like other class C GPCRs has a large extracellular domain (ECD) containing the ligand binding sites, a seven-transmembrane domain, and a large cytoplasmic C-terminal domain (Katritch et al., 2013), forms dimers and couples to multiple G-protein subtypes (e.g., $G\alpha_{q/11}$, $G\alpha_{i/o}$, $G\alpha_{12/13}$, and $G\alpha_s$) to induce diverse signaling pathways. For example, the CaSR, when stimulated by elevations in $Ca^{2+}{}_e$, signals predominantly via $G\alpha_{q/11}$ to activate phospholipase C (PLC), with consequent hydrolysis of phosphatidylinositol 4, 5-bisphosphate (PIP_2), to the second messengers inositol 1, 4, 5-trisphosphate (IP_3) and diacylglycerol (DAG) (Conigrave and Ward, 2013). IP_3 acts upon IP_3 receptors at the endoplasmic reticulum, allowing intracellular calcium ($Ca^{2+}{}_i$) mobilization into the cytosol, and DAG activates protein kinase C (PKC) signaling cascades, including mitogen-activated protein kinase (MAPK) pathways (Conigrave and Ward, 2013). CaSR has also been reported to signal via $G\alpha_{i/o}$ to inhibit adenylate cyclase (AC) and reduce cyclic AMP (cAMP) (Conigrave and Ward, 2013), $G\alpha_{12/13}$ to initiate cytoskeletal remodeling (Davies et al., 2006; Huang et al., 2004), and $G\alpha_s$, leading to elevated cAMP levels in breast cancer cell lines (Mamillapalli et al., 2008).

These CaSR signaling pathways are dependent on CaSR cell-surface expression, which is regulated by a balance between its plasma membrane (PM) insertion and removal by endocytosis (Grant et al., 2011). The PM insertion of CaSRs involves an anterograde signaling pathway, referred to as agonist-driven insertional signaling (ADIS), in which CaSRs that are continuously produced at the endoplasmic reticulum are rapidly trafficked to

 © 2017 The Author(s).
This is an open access article under the CC BY license (http://creativecommons.org/licenses/by/4.0/).

and inserted at the PM in the presence of high Ca^{2+}_e (Grant et al., 2011). Following activation, CaSRs have been reported to be endocytosed at a constant rate and targeted to the endo-lysosomal pathway for degradation (Grant et al., 2011). However, studies of patients with familial hypocalciuric hypercalcemia type-3 (FHH3), an autosomal dominant calcitropic disorder that is due to mutations of the σ subunit of the heterotetrameric adaptor protein-2 (AP2σ), which has a critical role in clathrin-mediated endocytosis (Nesbit et al., 2013b), have reported that FHH3-associated AP2σ mutations result in increased expression of the CaSR at the PM, which is paradoxically associated with reduced CaSR signaling via $G\alpha_{q/11}$ (Nesbit et al., 2013a). FHH is a genetically heterogeneous disorder, which is characterized by mild to moderate elevations in serum calcium concentrations, low urinary calcium excretion, and normal to elevated circulating parathyroid hormone (PTH), and the three recognized types, FHH1, FHH2, and FHH3, are due to loss-of-function mutations of the CaSR, $G\alpha_{11}$, and AP2σ, respectively (Hannan et al., 2016; Nesbit et al., 2013a, 2013b). FHH3-associated AP2σ mutations have been found to only occur at residue R15, and these comprise one of three missense mutations, R15C, R15H, or R15L, all of which would lead to a loss or weakening of a polar contact with the dileucine-based motif within cytoplasmic regions of membrane-associated cargo proteins and thereby impair their endocytosis (Kelly et al., 2008; Nesbit et al., 2013b). *In vitro* studies of these FHH3-associated mutations demonstrated that these AP2σ mutations decreased CaSR-mediated $G\alpha_{q/11}$ signaling in response to elevations in Ca^{2+}_e in cells expressing the mutants, despite increased CaSR cell-surface expression (Nesbit et al., 2013b).

To explain this paradox, we hypothesized that the FHH3-associated AP2σ mutations may be disrupting the contribution of endosomal sustained signaling to CaSR-dependent G-protein pathways, similar to those reported for some class A GPCRs—e.g., β2-adrenergic receptor (β2AR), dopamine receptor D1 (DRD1), thyroid-stimulating hormone receptor (TSHR), vasopressin receptor 2 (V2R), and luteinizing hormone receptor (LHR)—and class B GPCRs (e.g., parathyroid hormone 1 receptor, PTH1R) (Calebiro et al., 2009; Feinstein et al., 2013; Ferrandon et al., 2009; Irannejad et al., 2013; Jean-Alphonse et al., 2014; Kotowski et al., 2011). These components of the endocytic pathway, which have previously been considered endpoints for signaling, are now known to provide sites for sustained GPCR signals (Feinstein et al., 2013; Ferrandon et al., 2009), although the contribution of endomembrane sustained signaling to GPCR function has only been studied in the context of a single GPCR/G-protein pathway. However, GPCR signaling is complex, with many receptors (e.g., the CaSR) coupling to multiple G-protein-dependent and G-protein-independent pathways, and strategies to pharmacologically select for such specific pathways is increasingly recognized to be important (Rosenbaum et al., 2009). To further elucidate the role of the endocytic system in coordinating the pleiotropic activities of GPCRs, we investigated the effects of the FHH3-associated AP2σ mutations on the different G-protein pathways activated by CaSR and discovered that impaired internalization, by clathrin-mediated endocytosis of CaSR, differentially affects G-protein pathways of CaSR.

RESULTS

Establishing AP2σ Mutant Stable Cell Lines

To investigate further the effects of FHH3-associated AP2σ mutations on CaSR signaling and trafficking, HEK293 cells stably expressing AP2σ wild-type (WT; R15) or mutant (C15, H15, and L15) proteins were established, using appropriate pcDNA3.1-*AP2S1* constructs that also had silent mutations, which rendered them resistant to AP2σ-targeted small interfering RNA (siRNA), thereby allowing study of the mutant protein in the absence of endogenous protein. The presence of AP2σ mutant proteins or siRNA-resistant mutations did not affect expression of endogenous AP2α, AP2β, or AP2μ that with the σ subunit form the heterotetrameric AP2; general clathrin-mediated endocytic functions such as transferrin uptake; or internalization and signaling of another GPCR, the β2AR (Figure S1). These stably expressing AP2σ cells were transiently transfected with pEGFP-CaSR-WT (AP2σ/CaSR-WT) cells (Figure S1). All AP2σ mutant/CaSR-WT cells, when compared to AP2σ-WT/CaSR-WT cells, had a decreased sensitivity to increases in Ca^{2+}_e-induced Ca^{2+}_i, which is mediated by $G\alpha_{q/11}$, with significantly higher half-maximal effective concentration (EC_{50}) values (Figure S2). These results, which are in agreement with our previous results from HEK293 cells transiently expressing AP2σ mutants (Nesbit et al., 2013b), demonstrate that these stably expressing AP2σ mutant cells have impaired $G\alpha_{q/11}$-mediated, Ca^{2+}_e-induced Ca^{2+}_i release and that they are therefore suitable for studying the effects of FHH3-associated AP2σ mutations on CaSR signaling pathways and trafficking.

AP2σ Mutations Reduce $G\alpha_{q/11}$ Signaling

We hypothesized that Ca^{2+}_e-induced Ca^{2+}_i release of AP2σ mutant/CaSR-WT cells may be due to reduced calcium oscillations, and we assessed this by using single-cell microfluorimetry with the calcium-indicating dye Fura-2 in response to increasing concentrations (0–15 mM) of Ca^{2+}_e. CaSR-mediated Ca^{2+}_i oscillations were observed to occur from 1 to 5 mM Ca^{2+}_e, consistent with previous reports, but mutant cells were found to have reduced frequencies, with the AP2σ-C15 and AP2σ-L15 cells requiring higher Ca^{2+}_e concentrations to begin oscillating and AP2σ-H15 cells having oscillations with irregular amplitudes (Figures 1A and S2). Ca^{2+}_i release activates transcription factors such as nuclear factor of activated T cells (NFAT) (Chakravarti et al., 2012). Investigation of the effects of the FHH3-associated AP2σ mutations on gene transcription, using an NFAT-response element (RE)-containing luciferase reporter construct, revealed that the AP2σ mutant/CaSR-WT cells had significantly reduced concentration-dependent increases in NFAT reporter activity when compared to AP2σ-WT/CaSR-WT cells (Figure 1B). Similarly, assessment of the accumulation of inositol monophosphate (IP_1), an IP_3 metabolite, revealed reduced IP_1 in AP2σ mutant cells compared to AP2σ-WT cells (Figure S2), thereby indicating that the PLC-IP_3-DAG pathway is impaired in AP2σ mutant cells.

CaSR $G\alpha_{q/11}$-mediated signaling also activates MAPK pathways (Kifor et al., 2001). Investigation of the AP2σ mutant/CaSR-WT cells using AlphaScreen analyses of ERK1/2 phosphorylation (pERK1/2) in response to elevated Ca^{2+}_e

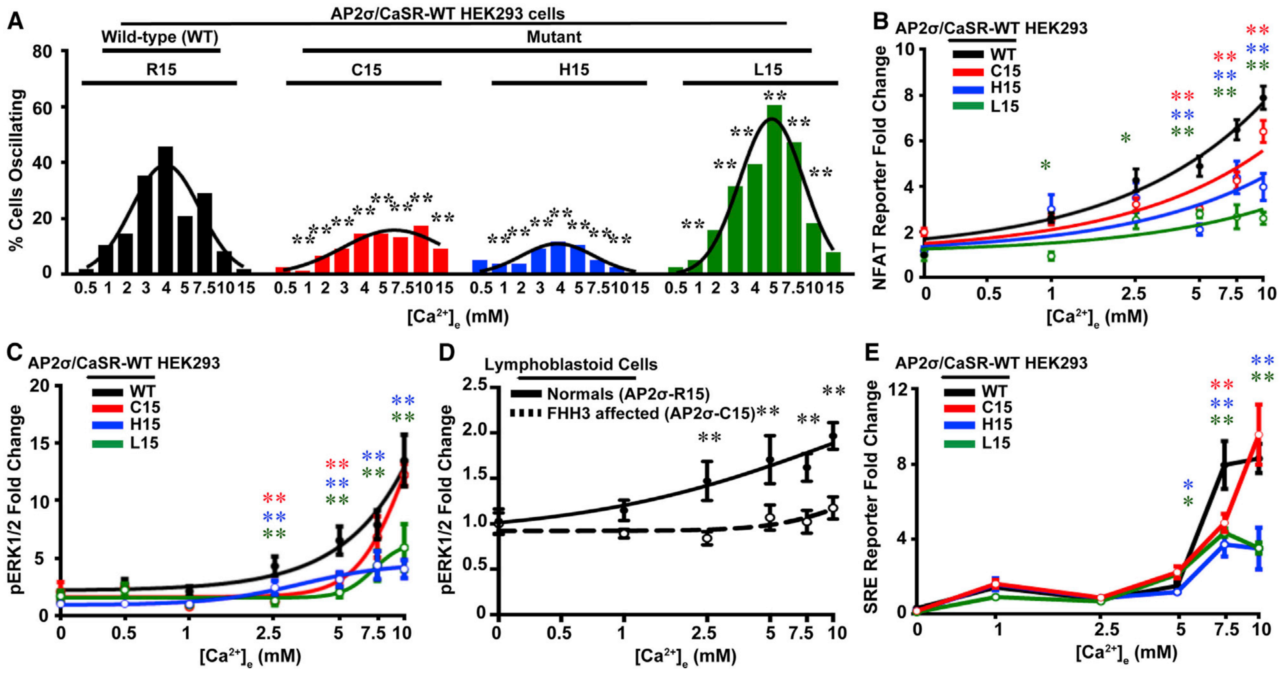

Figure 1. AP2σ-R15 Mutations Impair $G\alpha_{q/11}$ Signaling

(A) Number of oscillating cells measured by normalized Fura-2 ratios in response to increasing doses of Ca^{2+}_e in single AP2σ/CaSR-WT HEK293 cells that stably expressed AP2σ-wild-type (WT; R15) or mutant (C15, H15, or L15) proteins and transiently expressed pEGFP-CaSR-WT (n = 36–50 cells from 9 to 10 transfections). **p < 0.02 versus WT (χ^2 test) (Figures S1 and S2).

(B) Ca^{2+}_e-induced NFAT luciferase reporter responses in AP2σ/CaSR-WT HEK293 cells (n = 8).

(C) Ca^{2+}_e-induced phosphorylation of ERK1/2 (pERK1/2) measured by AlphaScreen (n = 4). AP2σ-WT/CaSR-WT cells had a dose-dependent increase in pERK1/2, which was reduced in AP2σ mutant/CaSR-WT cells within the range 2.5–5 mM Ca^{2+}_e in C15 cells and 2.5–10 mM Ca^{2+}_e in H15 and L15 cells.

(D) Ca^{2+}_e-induced pERK1/2 responses measured by AlphaScreen in EBV-transformed lymphoblastoid cells from members of the FHH3 kindred in which affected members have AP2σ-C15 mutations (Figure S3). Unaffected (normal) relatives (AP2σ-R15) were used as controls (n = 4).

(E) Ca^{2+}_e-induced SRE luciferase reporter responses in AP2σ/CaSR-WT HEK293 cells (n = 8).

(B–E) Data are shown as mean ± SEM with *p < 0.05 and **p < 0.02 (two-way ANOVA of WT versus mutants).

revealed them to have significant reductions in Ca^{2+}_e-induced pERK1/2 responses when compared to AP2σ-WT/CaSR-WT cells (Figure 1C). Moreover, pERK1/2 responses to increases in Ca^{2+}_e were reduced in Epstein-Barr virus (EBV)-transformed lymphoblastoid cells from FHH3 patients with the AP2σ-R15C mutation (Figures 1D and S3), consistent with findings from AP2σ mutant/CaSR-WT cells. Expression of the AP2σ subunit genes and proteins was similar in lymphoblastoids from FHH3 patients with the AP2σ-R15C and unaffected relatives, indicating that the AP2σ-R15C mutation was not affecting the stability of the AP2 complex (Figure S3). ERK1/2 activates genes containing serum response elements (SREs) (Pi et al., 2002). Use of a SRE luciferase reporter revealed the AP2σ mutant/CaSR-WT cells have reduced SRE reporter activity (p < 0.02) (Figure 1E), with the more severe effects being observed in AP2σ-H15 and AP2σ-L15 mutant cells. Thus, these results demonstrate that the FHH3-associated AP2σ mutations cause a reduction in $G\alpha_{q/11}$ signaling via both the IP_3 and the DAG pathways.

CaSR-Mediated cAMP Responses Are Altered by AP2σ Mutations

CaSR activation of the $G\alpha_{i/o}$ pathway inhibits adenylate cyclase and reduces cAMP, and we assessed the effects of the FHH3-associated AP2σ mutations using AlphaScreen analysis to measure Ca^{2+}_e-induced cAMP responses. Ca^{2+}_e was first confirmed to reduce cAMP responses, which were pertussis toxin (PTx) sensitive and therefore due to $G\alpha_{i/o}$ signaling, in HEK293 cells stably expressing CaSR (HEK-CaSR) (Figure 2A). However, $G\alpha_{i/o}$ inhibition only partially affected cAMP production, and treatment with UBO-QIC, an inhibitor of $G\alpha_{q/11}$, revealed that the Ca^{2+}_e-induced reduction in cAMP was also sensitive to $G\alpha_{q/11}$ inhibition, thereby indicating a hitherto unreported role for $G\alpha_{q/11}$ (Figure 2B). Moreover, combined treatment of cells with both UBO-QIC and PTx halted all Ca^{2+}_e-induced reductions in cAMP (Figure 2B) indicating that G proteins other than $G\alpha_{q/11}$ and $G\alpha_{i/o}$ are unlikely to be involved in this CaSR pathway. However, UBO-QIC has been reported to inhibit Gβγ, in addition to $G\alpha_{q/11}$ (Gao and Jacobson, 2016), but gallein, an inhibitor of Gβγ, had no effect on cAMP signaling (Figure 2C), thereby indicating that Gβγ is unlikely to have a role in CaSR-mediated cAMP reductions. Increases in $[Ca^{2+}]_e$ also led to a dose-dependent reduction in cAMP in AP2σ-WT/CaSR-WT cells, but not in AP2σ mutant/CaSR-WT cells, with cAMP in AP2σ-C15/CaSR-WT cells remaining at basal levels (Figure 2D) and with AP2σ-H15/CaSR-WT and AP2σ-L15/CaSR-WT cells responding

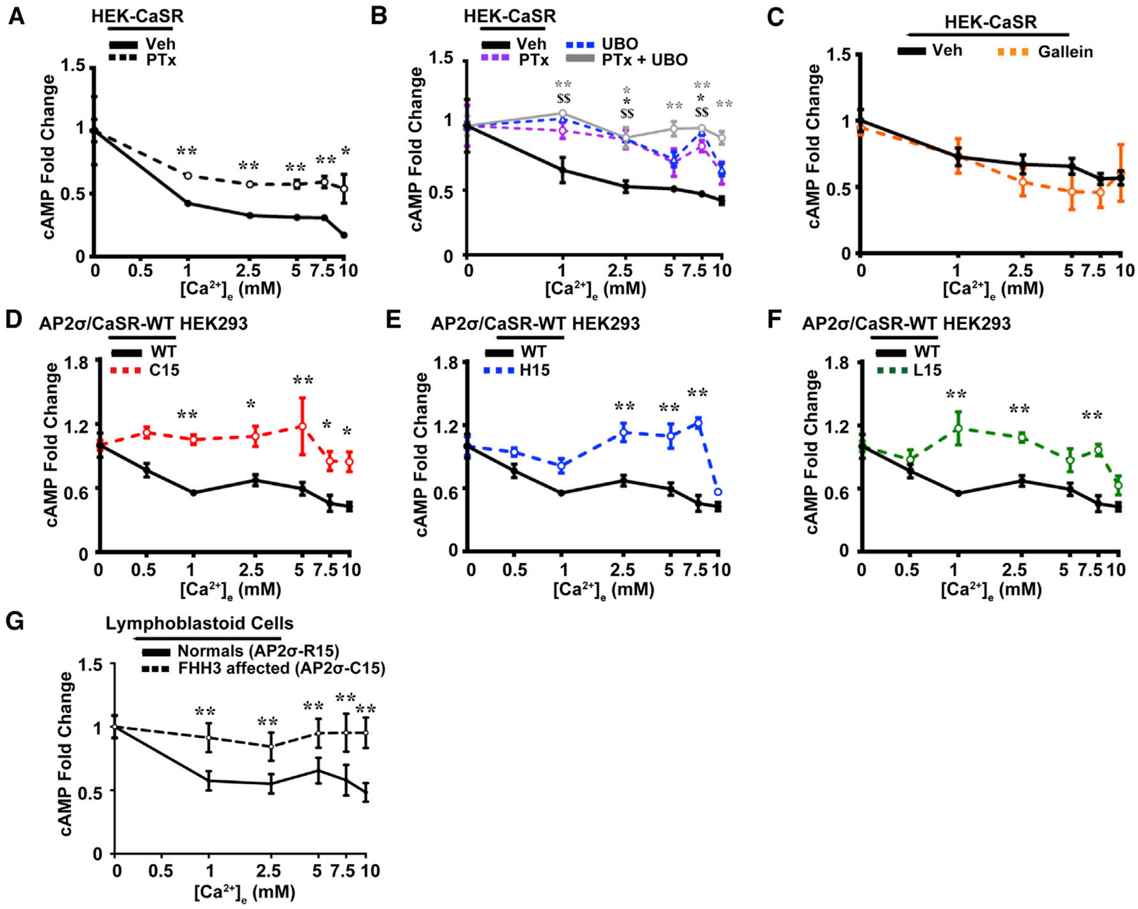

Figure 2. AP2σ-R15 Mutations Impair the G$\alpha_{i/o}$ Signaling Pathway

$Ca^{2+}{}_e$-induced cAMP inhibition was measured by AlphaScreen.

(A) Effect of ethanol-diluent (vehicle, veh) or pertussis toxin (PTx) on $Ca^{2+}{}_e$-induced cAMP inhibition in HEK-CaSR-WT cells. PTx inhibits G$\alpha_{i/o}$-mediated, $Ca^{2+}{}_e$-induced cAMP reductions (n = 4).

(B) Effect of veh, PTx, the G$\alpha_{q/11}$ inhibitor UBO-QIC (UBO), or combined PTx and UBO treatment on $Ca^{2+}{}_e$-induced cAMP inhibition in HEK-CaSR-WT cells (n = 4).

(C) Effect of DMSO (vehicle, veh) or the G$\beta\gamma$ inhibitor gallein on $Ca^{2+}{}_e$-induced cAMP inhibition in HEK-CaSR-WT cells. Gallein did not significantly alter $Ca^{2+}{}_e$-induced cAMP responses when compared to vehicle (n = 4).

(D–F) $Ca^{2+}{}_e$-induced cAMP inhibition in AP2σ-WT/CaSR-WT and AP2σ mutant/CaSR-WT HEK293 cells. AP2σ mutant cells—(D) C15, (E) H15, and (F) L15—had impaired responses when compared to WT (AP2σ-R15) cells (n = 8–12).

(G) $Ca^{2+}{}_e$-induced cAMP inhibition in EBV-transformed lymphoblastoid cells from FHH3 patients, with AP2σ-C15 mutation, and unaffected (normal) relatives (Figure S3).

Data are shown as mean ± SEM with *$p < 0.05$ and **$p < 0.02$ (two-way ANOVA comparing WT versus mutant in AP2σ HEK293 cells and normal versus FHH3 affected in lymphoblastoid cells). (B) shows vehicle versus PTx (black asterisk), UBO (dollar signs), and combined PTx and UBO (gray asterisks).

with reductions in cAMP (Figures 2E and 2F). Moreover, lymphoblastoid cells from FHH3 patients with the AP2σ-R15C mutation, when compared to those from normal relatives, did not have $Ca^{2+}{}_e$-induced cAMP responses (Figure 2G), consistent with findings from the AP2σ-C15/CaSR-WT cells. Thus, the FHH3-associated AP2σ mutants reduce G$\alpha_{i/o}$- and G$\alpha_{q/11}$-mediated effects on cAMP responses.

AP2σ Mutations Reduce Membrane Ruffling

CaSR has been reported to induce cytoskeletal changes such as membrane ruffling by both G$\alpha_{q/11}$ and G$\alpha_{12/13}$ signaling (Bouschet et al., 2007; Huang et al., 2004; Pi et al., 2002). We therefore investigated the effects of FHH3-associated AP2σ mutants on membrane ruffling, using AP2σ mutant/CaSR-WT cells and phalloidin-594 as an actin marker. Elevations of $Ca^{2+}{}_e$ increased membrane ruffling in AP2σ-WT and mutant cells, although AP2σ mutant cells had significantly reduced membrane ruffling compared to WT cells ($p < 0.02$) (Figures 3A and S4). Assessment of membrane ruffling-induced gene transcription (Tojkander et al., 2012) using a serum response factor (SRF)-RE reporter construct revealed AP2σ mutant cells to have significantly reduced SRF activity compared to AP2σ-WT cells (Figure 3B). Further investigation of SRF reporter assays in HEK293 cells transiently expressing CaSR but depleted of G$\alpha_{q/11}$, G$\alpha_{12/13}$, or G$\alpha_{q/11/12/13}$ revealed SRF activity to be abolished in G$\alpha_{q/11}$ and G$\alpha_{q/11/12/13}$ knockout cells but to be significantly higher in

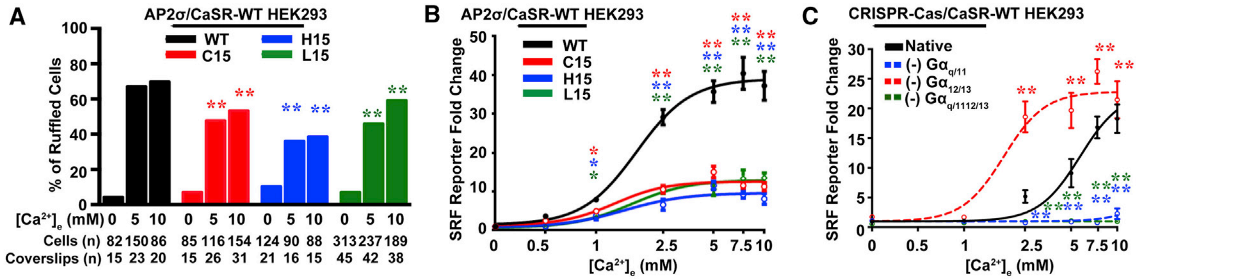

Figure 3. AP2σ-R15 Mutations Impair Membrane Ruffling via Reduction in $G\alpha_{q/11}$ Signaling

(A) Percentage of AP2σ/CaSR-WT cells with membrane ruffling (Figure S4) at each $Ca^{2+}{}_e$ concentration measured. Numbers (n) of cells—AP2σ-WT (R15) or mutant (C15, H15, or L15)—and coverslips are indicated. $^{**}p < 0.02$ (χ^2 test).

(B) $Ca^{2+}{}_e$-induced SRF luciferase reporter activity (n = 8). Responses were reduced in AP2σ mutant cells.

(C) $Ca^{2+}{}_e$-induced SRF luciferase reporter activity in native HEK293 cells or CRISPR-Cas gene-edited HEK293 knockout cells of $G\alpha_{q/11}$, $G\alpha_{12/13}$, or $G\alpha_{q/11/12/13}$ transfected with pEGFP-CaSR-WT. (−) denotes genes deleted. SRF reporter activity was abolished in cells depleted of $G\alpha_{q/11}$ and $G\alpha_{q/11/12/13}$ but elevated in cells depleted of $G\alpha_{12/13}$.

Data are shown as mean ± SEM (n = 8) with $^*p < 0.05$ and $^*p < 0.02$ (two-way ANOVA of WT, or native, versus mutant).

$G\alpha_{12/13}$ knockout cells than in native cells (Figure 3C). Moreover, quantification of membrane ruffling in $G\alpha_{12/13}$ knockout cells and native HEK293 cells transiently expressing CaSR showed them to have similar levels of ruffling (Figure S4), thereby indicating the existence of $G\alpha_{12/13}$-independent ruffling pathways. Overall, these results indicate that $Ca^{2+}{}_e$-induced membrane ruffling in HEK293 expressing CaSR is mediated by $G\alpha_{q/11}$ signaling and that FHH3-associated AP2σ mutations, which impair $G\alpha_{q/11}$ signaling, reduce membrane ruffling.

AP2σ Mutations Impair CaSR Internalization and Differentially Affect CaSR Cell-Surface Expression, which Both Require $G\alpha_{q/11}$

FHH3-associated AP2σ mutations have been reported to result in increased CaSR cell-surface expression, which represents the net balance between its PM insertion by ADIS and removal by endocytosis (Grant et al., 2011). We therefore simultaneously measured the effects of the FHH3-associated AP2σ mutations on ADIS and endocytosis by transfecting AP2σ-WT and AP2σ mutant cells with a plasmid construct containing full-length CaSR, with an N-terminal modification that in tandem comprised a minimal α-bungarotoxin (BTx)-binding site to monitor endocytosis and superecliptic pHluorin (SEP) to monitor total cell-surface CaSR, referred to as BSEP-CaSR (Figure 4A) (Grant et al., 2011). Total internal reflection fluorescence (TIRF) microscopy was used to assess CaSR cell-surface expression under basal (0.1 mM $Ca^{2+}{}_e$) conditions or following exposure to 5 or 10 mM $Ca^{2+}{}_e$. Immediately before TIRF microscopy continuous recordings, cells were exposed to BTx with a fluorescent tag (BTx-594). AP2σ-WT and mutant cells expressed CaSR at the cell surface (Figures 4B and 4C), and both 5 and 10 mM $Ca^{2+}{}_e$ induced elevations in SEP fluorescence and reductions in BTx-594. These were greater at 10 mM $Ca^{2+}{}_e$, which was used for subsequent imaging experiments (Figures 4B, 4C, and S5). Thus, elevations in $Ca^{2+}{}_e$ increased CaSR PM insertion (Figures 4B and 4C), and returning $Ca^{2+}{}_e$ to basal conditions induced a reduction in cell surface CaSR, observed by a decline in SEP fluorescence (Figure 4C). Maximal SEP fluorescence in AP2σ-C15 cells was similar to WT, but AP2σ mutant L15 cells had reduced SEP fluorescence and H15 cells had significantly higher CaSR PM expression ($p < 0.01$, F test) (Figures 4B and 4C). All AP2σ mutant cells had slower declines in BTx-594 PM fluorescence when compared to AP2σ-WT cells, thereby indicating delayed internalization (Figure 4D). The time to internalize 75% of the BTx-594 at the PM was significantly increased from 268 s in AP2σ-WT to 346, 741, and 350 s in AP2σ-C15, AP2σ-H15, and AP2σ-L15 mutant cells, respectively ($p < 0.05$ to $p < 0.02$) (Figure 4E). This was greatest in the AP2σ-H15 cells, which may partly account for the very high CaSR PM expression in these cells (Figure 4C). Moreover, TIRF microscopy analysis of $G\alpha_{q/11}$ knockout cells transfected with BSEP-CaSR showed that the $Ca^{2+}{}_e$-induced increase in SEP fluorescence (i.e., increased CaSR PM expression via ADIS) was lost and that CaSR internalization measured by BTx-594 fluorescence was severely impaired (Figures 4F and 4G). These findings indicate that $G\alpha_{q/11}$ signaling is required for ADIS responses and that CaSR endocytosis requires a signal within the $G\alpha_{q/11}$ pathway for its maintenance.

CaSR Delayed Internalization Is due to Prolonged CaSR-Clathrin Colocalization in AP2σ Mutant Cells

AP2σ mutants impair but do not abolish CaSR internalization (Figure 4), indicating that AP2 and clathrin are still recruited to the forming endocytic pit but that CaSR internalization occurs at a slower rate. We therefore predicted that the duration of colocalization between CaSR and clathrin may be prolonged, reflecting this slower internalization rate. We investigated this by transfecting AP2σ mutant and AP2σ-WT cells with BSEP-CaSR and dsRed-Clathrin and analyzed colocalization by TIRF microscopy. Clathrin fluorescence increased in the AP2σ-WT and AP2σ mutant cells during the TIRF microscopy recording, indicating that clathrin is recruited to the PM, although the increase in clathrin recruitment to the PM was significantly greater in AP2σ-WT than in AP2σ mutant cells ($p < 0.02$) (Figure 5A). Vesicles containing both clathrin and CaSR were analyzed for motility, because higher motility is associated with increased

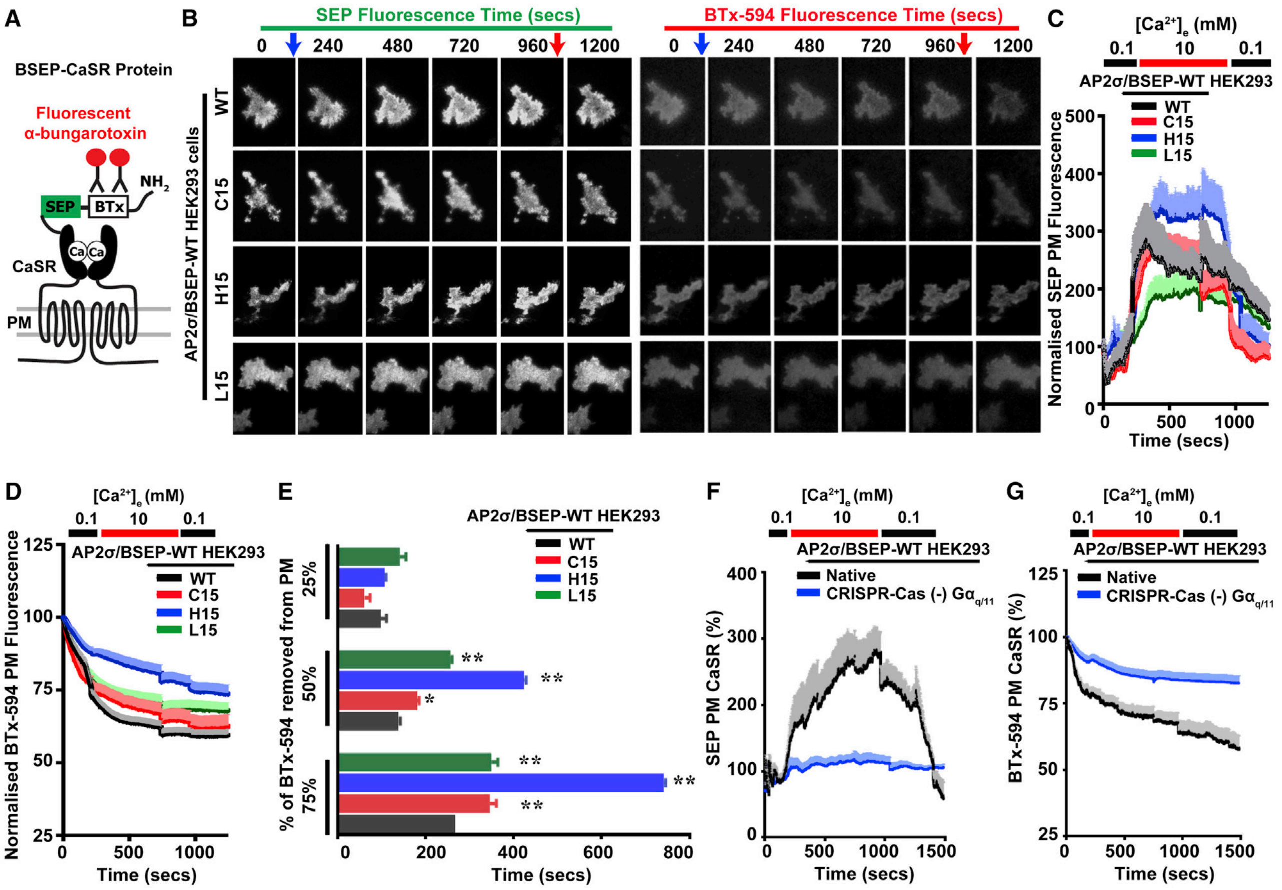

Figure 4. AP2σ-R15 Mutations Impair CaSR Internalization

TIRF microscopy analyses in AP2σ-WT (R15) or mutant (C15, H15, or L15) HEK293 cells transfected with BSEP-CaSR.

(A) Schematic diagram of BSEP-CaSR. BSEP-CaSR encodes CaSR with an N-terminal modification of a minimal bungarotoxin (BTx) binding site, to which BTx-594 binds to measure endocytosis, and superecliptic pHluorin (SEP), which maximally fluoresces at neutral pH and measures total cell surface CaSR.

(B) TIRF microscopy images of SEP and BTx-594 fluorescence. Blue arrows indicate addition of 10 mM, and red arrows the return to 0.1 mM $Ca^{2+}{}_e$.

(C and D) Quantification of fluorescence in each movie frame for (C) SEP and (D) BTx-594 images. $[Ca^{2+}]_e$ is shown above. Data are normalized to the fluorescence in the first frame of each movie (set at 100%). Data are shown as mean + SEM.

(E) Time taken to reduce BTx-594 expression by 25%, 50%, and 75%.

(F and G) TIRF microscopy analyses in native HEK293 cells or CRISPR-Cas gene-edited HEK293 cells of $G\alpha_{q/11}$ transfected with BSEP-CaSR. Quantification of fluorescence in each movie frame for (F) SEP and (G) BTx-594 images. $[Ca^{2+}]_e$ is shown above. (−) denotes genes deleted. Cells depleted of $G\alpha_{q/11}$ had impaired ADIS and endocytosis. Data are shown as mean + SEM with $*p < 0.05$ and $**p < 0.02$ for comparison to WT (two-way ANOVA).

likelihood of viable endocytic events (Rappoport and Simon, 2003). Vesicles that had both CaSR and clathrin were highly motile in AP2σ-WT cells, which had a greater proportion of highly motile CaSR-clathrin-containing vesicles than AP2σ-H15 and AP2σ-L15 cells; instead, these AP2σ mutant cells had a significantly greater number of non-motile CaSR-clathrin-containing vesicles ($p < 0.02$) (Figures 5B and 5C). The reduced motility of the CaSR-clathrin-containing positive vesicles in AP2σ mutant cells would delay vesicle internalization and thereby likely prolong the colocalization of CaSR and clathrin in clathrin-coated pits. Assessment of the duration of CaSR-clathrin colocalization in individual vesicles revealed that all AP2σ mutant cells, when compared to AP2σ-WT cells, had prolonged CaSR-clathrin associations (Figure 5D). However, motile vesicles in AP2σ-WT and AP2σ-C15 cells had a significantly shorter duration of colocalization when compared to non-motile vesicles, indicating that these motile vesicles are likely resulting in endocytic events, although there was no significant difference between motile and non-motile vesicles in H15 and L15 cells (Figure 5D). These results indicate that CaSR internalization is impaired in AP2σ mutant cells at distinct stages of endocytosis by prolonged residency time at clathrin-coated pits and/or vesicles.

CaSR Is Able to Induce Sustained Signaling from a Cytoplasmic Location

The FHH3-associated AP2σ mutations resulted in impaired CaSR-induced signaling (Figures 1, 2, and 3), despite increased CaSR cell-surface expression (Figure 4) due to delayed internalization. This led us to hypothesize that CaSR signaling may

OPEN ACCESS
CellPress

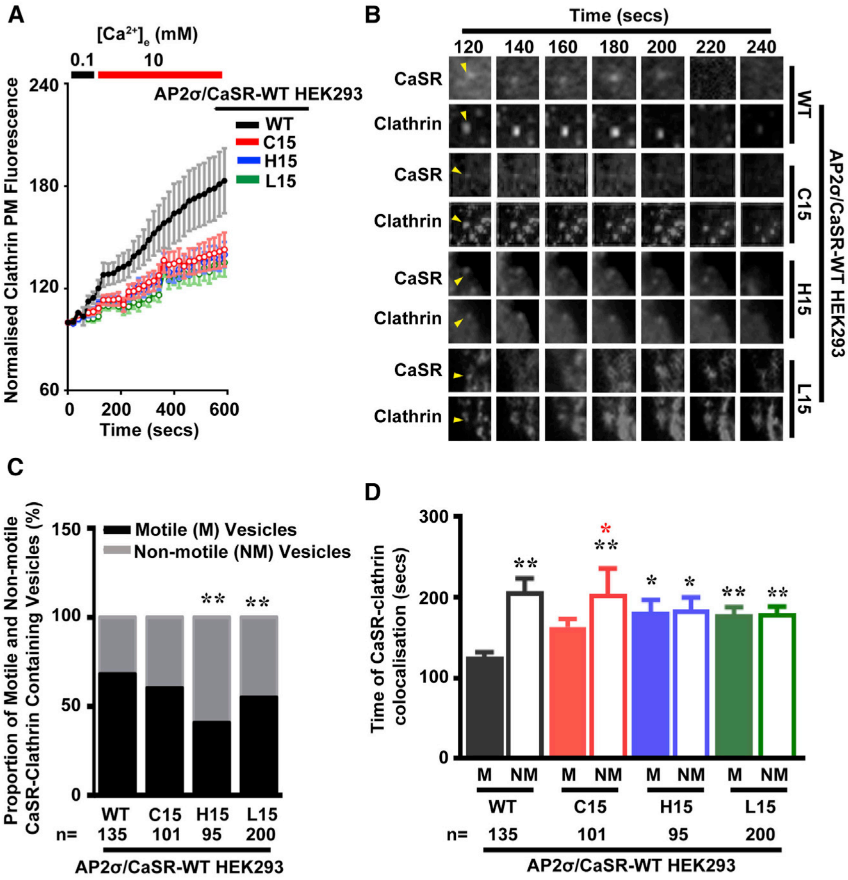

Figure 5. Impairments in CaSR Internalization Are due to Prolonged CaSR-Clathrin Co-localization

TIRF microscopy analyses of colocalized CaSR (BSEP-CaSR) and Clathrin (dsRed-Clathrin) performed in AP2σ-WT (R15) or mutant (C15, H15, or L15) cells.

(A) Quantification of clathrin fluorescence with changes in $[Ca^{2+}]_e$ (shown above). Data are normalized to the fluorescence in the first frame of each movie (set at 100%). Data shown as mean ± SEM.

(B) Images of CaSR and clathrin expression in single vesicles (yellow arrow).

(C and D) Proportion of motile (M) versus non-motile (NM) CaSR and clathrin-containing vesicles (C), and duration of colocalization between CaSR and Clathrin in individual (motile, M, filled box, and non-motile, NM, open box) vesicles (D).

Data from 95 to 200 vesicles (n = 14–16 recordings) are expressed as mean ± SEM with $^{*}p < 0.05$ and $^{**}p < 0.02$ (two-way ANOVA) illustrated by black and red asterisks for WT motile versus mutant motile vesicles and C15 motile versus non-motile vesicles, respectively.

require, or be enhanced, by receptor internalization that would contribute to sustained (i.e., non-canonical) signaling. To test this hypothesis, we treated HEK293-CaSR cells with the dynamin-blocking agent Dyngo, which would abolish endocytosis and prevent endosomal signaling, and assessed their MAPK signaling responses by measurement of pERK1/2 to a 5 min pulse of 5 mM Ca^{2+}_e. pERK1/2 accumulated in Dyngo-treated and control DMSO-treated cells from 2 to 5 min and then rapidly decreased in Dyngo-treated cells, but not DMSO-treated cells; in the latter, pERK1/2 remained significantly increased at 30 min, indicating a potential sustained signaling response (Figures 6A, 6B, and S5). Loss of this sustained response in Dyngo-treated cells was not due to increased apoptosis, decreased proliferation, or inhibition of CaSR protein synthesis, because the sustained rise in pERK1/2 was not blocked by tunicamycin (Figure S5). The effects of this sustained pERK1/2 signaling on transcription were investigated by SRE reporter activity in HEK-CaSR cells treated with constant or 5 min pulsed elevations in Ca^{2+}_e. Constant treatment with 5 mM Ca^{2+}_e, when compared to 0.1 mM Ca^{2+}_e, resulted in rapid increases in SRE reporter activity that peaked between 4 and 6 hr, after which they rapidly reduced (Figure 6C). However, pulsed elevations with 5 and 7.5 mM, followed by incubation with basal 0.1 mM Ca^{2+}_e for 0–12 hr, resulted in a peaked response between 4 and 6 hr that was followed by a second peaked response at 9 hr, consistent with a sustained signaling response (Figure 6D). Treatment with Dyngo abolished the second peaked response in HEK-CaSR cells given a 5 min pulse of 5 mM Ca^{2+}_e (Figure 6E), thereby indicating that the sustained signaling response was likely originating from endosomes. An endosomal origin of this sustained response was further investigated by measuring pERK1/2 responses at 5 and 30 min in HEK-CaSR cells overexpressing the early endosome guanosine triphosphatase (GTPase) Rab5; a dominant-negative (DN) S34N guanosine diphosphate (GDP)-bound form, which delays endocytosis by retaining cargo in clathrin-coated pits (CCPs); and a constitutively active (CA) Q79L form, which enhances endocytic processes (Galperin and Sorkin, 2003; Stenmark et al., 1994). Rab5 was shown to be overexpressed by these constructs, and confocal microscopy showed that FLAG-CaSR-WT internalized over time in response to 5 mM Ca^{2+}_e and partially colocalized with Rab5-WT-containing structures (Figure S6). Expression of Rab5-WT did not affect CaSR internalization, while the Rab5-DN protein delayed and reduced receptor internalization (Figure S6). In addition, HEK-CaSR cells expressing Rab5-CA when compared to Rab5-WT had enhanced pERK1/2 signals at 5 and 30 min, while Rab5-DN had reduced pERK1/2 signals at 30 min (Figures 6F and 6G). Furthermore, investigation of SRE reporter responses showed that the Rab5-DN reduced overall CaSR-driven SRE reporter activity (Figure 6H), which was due to loss of the sustained signal at 9 hr rather than reduction in immediate signaling (Figure 6I). MAPK signaling can be activated via $G\alpha_{q/11}$ and $G\alpha_{i/o}$ pathways (Figure S5) (Holstein et al., 2004). To assess the contribution of $G\alpha_{q/11}$ and $G\alpha_{i/o}$ signaling to sustained endosomal signaling, we measured SRE reporter activity in HEK-CaSR cells treated with UBO-QIC, an inhibitor of $G\alpha_{q/11}$, or PTx, a specific inhibitor

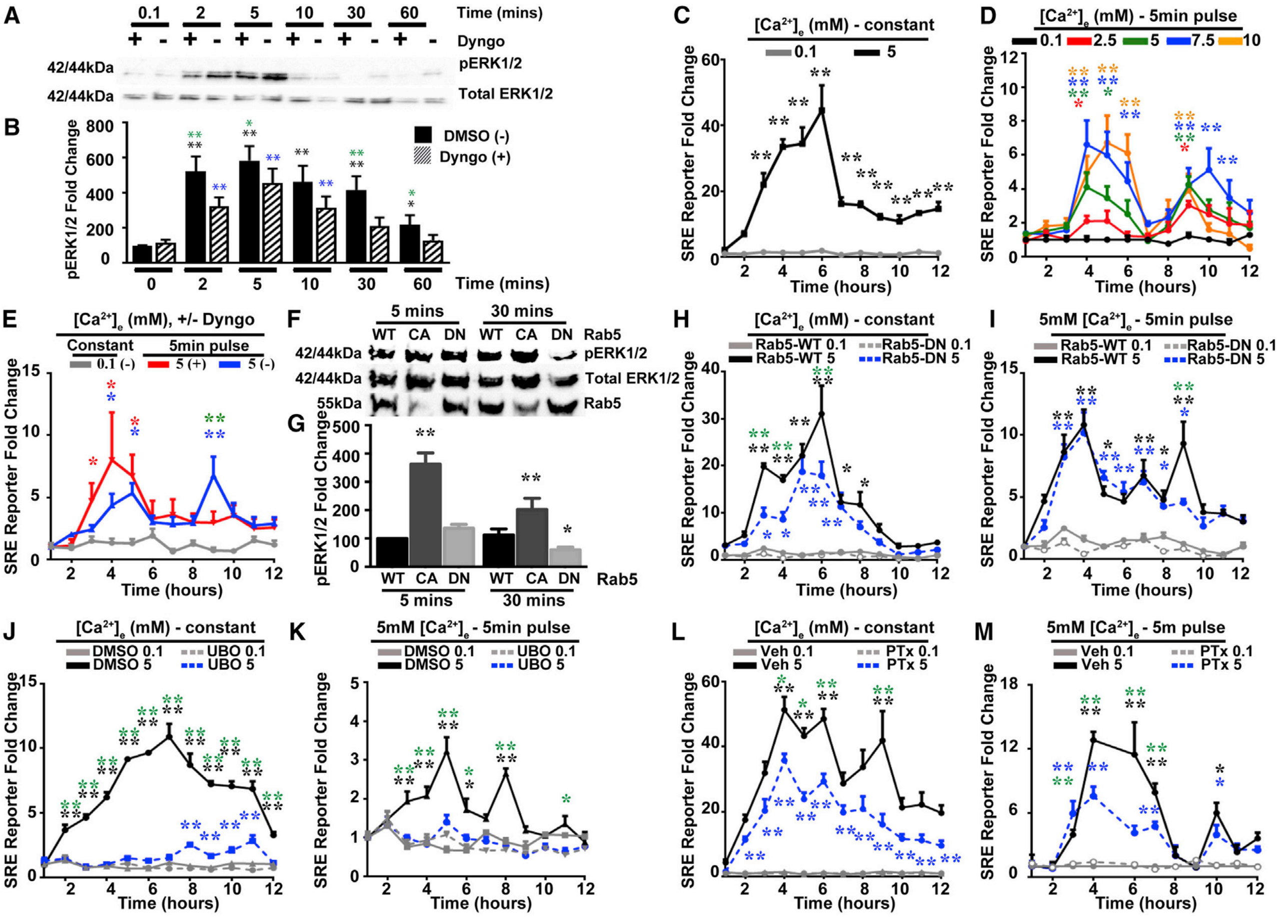

Figure 6. Second Signal of CaSR Is from the Rab5-Endosomal Internalization Pathway

(A) Effects of dynamin inhibitor Dyngo on MAPK signaling by western blot analyses of pERK1/2 responses in HEK-CaSR cells treated with Dyngo (+) or DMSO (−), given a 5 min pulse of 5 mM Ca^{2+}_e, and then incubated in 0.1 mM Ca^{2+}_e.

(B) Densitometry analysis showing data from blots (n = 8). Black and blue asterisks indicate p values of response versus response at 0 min for DMSO and Dyngo treated, respectively; green asterisks indicate DMSO versus Dyngo responses.

(C) SRE luciferase reporter responses to treatment of either 0.1 or 5 mM Ca^{2+}_e over 12 hr in HEK-CaSR cells. Asterisks indicate p values of response versus response to 0.1 mM (n = 4).

(D) SRE luciferase reporter activity in response to 5 min pulses of 0–10 mM Ca^{2+}_e in HEK-CaSR cells. Asterisks indicate p values of 0.1 mM responses versus 2.5 mM (red), 5 mM (green), 7.5 mM (blue), and 10 mM (yellow) (two-way ANOVA) (n = 4). Both initial and sustained peaks were enhanced by increasing concentrations of Ca^{2+}_e, which plateaued at 7.5 mM. Subsequent experiments were performed at Ca^{2+}_e = 5 mM.

(E) SRE luciferase reporter responses to a 5 min pulse of 0.1 or 5 mM Ca^{2+}_e with DMSO (−) or Dyngo (+) in HEK-CaSR cells. DMSO (blue)-treated cells and Dyngo (red)-treated cells had a peak at 4 hr, while the second peak at 9 hr was abolished by treatment with Dyngo. Asterisks indicate p values of 0.1 mM Ca^{2+}_e versus DMSO (blue) or Dyngo (red) and DMSO versus Dyngo (green) (two-way ANOVA).

(F) Western blot analysis of pERK1/2 responses in HEK-CaSR cells exposed for 5 or 30 min to 5 mM Ca^{2+}_e. Cells were transiently transfected with the Rab5 WT (S34/Q79) or the constitutively active (CA; L79) or dominant-negative (DN; N34) Rab5 mutants.

(G) Densitometric analyses of pERK1/2 in western blots (n = 4). Asterisks indicate p values of mutants compared to WT responses at each time point (two-way ANOVA). Rab5-CA had higher expression of pERK1/2 after 5 and 30 min of treatment, while Rab5-DN had lower pERK1/2 responses after 30 min.

(H) SRE luciferase reporter responses to treatment of 0.1 or 5 mM Ca^{2+}_e over 12 hr in HEK-CaSR cells transiently transfected with Rab5-WT or Rab5-DN mutant (n = 8).

(I) SRE luciferase reporter response to 5 min pulses of 0.1 or 5 mM Ca^{2+}_e in HEK-CaSR cells transiently transfected with Rab5-WT or Rab5-DN mutant (n = 8).

(J) SRE luciferase reporter responses to treatment of 0.1 or 5 mM Ca^{2+}_e over 12 hr in HEK-CaSR cells treated with DMSO or the $G\alpha_{q/11}$ inhibitor UBO-QIC (UBO) (n = 4).

(K) SRE luciferase reporter response to 5 min pulses of 0.1 or 5 mM Ca^{2+}_e in HEK-CaSR cells treated with DMSO or UBO (n = 4).

(L) SRE luciferase reporter responses to treatment of 0.1 or 5 mM Ca^{2+}_e over 12 hr in HEK-CaSR cells treated with vehicle (Veh) or PTx, a $G\alpha_{i/o}$ inhibitor (n = 8).

(M) SRE luciferase reporter response to 5 min pulses of 0.1 or 5 mM Ca^{2+}_e in HEK-CaSR cells treated with Veh or PTx (n = 8).

Rab5-DN, UBO, and PTx all reduced constant Ca^{2+}_e responses. In (H)–(M), asterisks show basal 0.1 mM Ca^{2+}_e responses versus 5 mM Ca^{2+}_e responses in Rab5-WT-, DMSO-, or Veh-treated cells (black); basal 0.1 mM Ca^{2+}_e responses versus 5 mM Ca^{2+}_e responses in Rab5-DN-, UBO-, or PTx-treated cells (blue); and Rab5-WT versus Rab5-DN, DMSO versus UBO, or Veh versus PTx (green) (two-way ANOVA). **$p < 0.02$, *$p < 0.05$. Rab5-DN and UBO reduced the sustained MAPK signal, while PTx had no effect on the sustained signal.

of $G\alpha_{i/o}$ (Figures 6J–6M). In the presence of constant 5 mM $Ca^{2+}{}_e$, SRE reporter activity was reduced in UBO-QIC- and PTx-treated cells compared to vehicle-treated cells (Figures 6J and 6L). However, in cells treated with a 5 min pulse of 5 mM $Ca^{2+}{}_e$, UBO-QIC and PTx similarly impaired the early SRE response (Figures 6K and 6M), but only UBO-QIC reduced the sustained signal, which was not affected by PTx (Figures 6K and 6M). Thus, these findings indicate that $G\alpha_{i/o}$ does not contribute to the sustained MAPK response from endosomes, which solely involves $G\alpha_{q/11}$. The presence of $G\alpha_{q/11}$ signaling pathway components in endosomes containing internalized CaSR was confirmed by using HEK293 cells transfected with FLAG-tagged CaSR and either $G\alpha_q$-Venus or a known GFP-tagged biosensor of PIP_2 (the lipid catalyzed by PLC), which contains the pleckstrin homology domain of PLC-delta (PH-PLC) (Stauffer et al., 1998). Before addition of 5 mM $Ca^{2+}{}_e$, colocalization of CaSR with either $G\alpha_q$ or PH-PLC was observed only at the PM; however, following treatment with 5 mM $Ca^{2+}{}_e$ for 10 and 30 min, a subpopulation of CaSR-containing endosomes that colocalized with $G\alpha_q$ or PH-PLC was detected, thereby indicating that internalized CaSR endosomes have $G\alpha_{q/11}$ signaling components (Pearson's correlation coefficients = 0.658 ± 0.027 for CaSR/$G\alpha_q$ and 0.652 ± 0.024 for CaSR/PH-PLC at 10 min and 0.693 ± 0.049 for CaSR/$G\alpha_q$ and 0.743 ± 0.059 for CaSR/PH-PLC at 30 min; n = 8–15) (Figure S6). To further assess the role of PLC in sustained signaling, we measured the effect of inhibitors of the PLC-DAG-IP_3 pathway (Figure S7) on pERK1/2 responses. HEK-CaSR cells were pulsed with 5 mM $Ca^{2+}{}_e$ and then treated with DMSO or with U73122, GF-109203X (GFX), or 2-aminoethoxydiphenyl borate (2-APB), which inhibits PLC, PKC, or the IP_3 receptor (IP_3R), respectively (Figure S7). pERK1/2 accumulated in all cells from 2 to 5 min, and sustained responses were observed in DMSO-treated cells but were significantly reduced in U73122, GFX, and 2-APB-treated cells (Figure S7), thereby confirming the requirement of this $G\alpha_{q/11}$ effector for sustained signaling. Finally, we assessed the effects of the scaffold proteins βarrestin-1 and βarrestin-2, which are important for endosomal signaling of GPCRs such as V2R and PTH1R (Feinstein et al., 2013; Wehbi et al., 2013), on the sustained signaling in HEK-CaSR cells and HEK293 cells that had deletions of βarrestin-1 and βarrestin-2, which were generated by CRISPR-Cas and stably overexpressed CaSR (Figure S7). The pERK1/2 and SRE reporter responses to a 5 min pulse of $Ca^{2+}{}_e$ in these cells lacking βarrestin-1 and βarrestin-2 showed no difference in responses when compared to WT cells, thereby indicating that βarrestin-1 and βarrestin-2 are not required for the CaSR sustained signal (Figure S7).

AP2σ-R15 Mutations Impair Sustained Endosomal Signaling

FHH3-associated AP2σ mutations impair CaSR signaling and internalization. We hypothesized that these AP2σ mutations were inhibiting sustained endosomal CaSR signaling and tested this by measuring the non-canonical SRE reporter responses in AP2σ-WT/CaSR-WT and AP2σ mutant/CaSR-WT cells treated with Dyngo, or overexpressing DN Rab5 (Figures 7A and S6). In the presence of constant 5 mM $Ca^{2+}{}_e$, SRE reporter responses were significantly higher in AP2σ-WT than in mutant cells, with peak expression occurring between 3 and 5 hr, in all cell lines (Figure 7A). Measurements of SRE reporter activity following a 5 min pulse of 5 mM $Ca^{2+}{}_e$ showed that the second Dyngo-sensitive peak was significantly reduced in C15 cells and abolished in H15 and L15 cells compared to WT cells (Figure 7B), thereby revealing that the FHH3-associated AP2σ mutations impaired early and sustained endosomal signaling. Moreover, the reduced sustained signaling in AP2σ-C15 cells was abolished by Rab5-DN, further demonstrating the endosomal origin of the sustained signaling (Figure S6). In summary, our results show that CaSR can induce sustained MAPK signaling from Rab5 endosomes and that FHH3-associated AP2σ mutations (C15, H15, and L15) impair $Ca^{2+}{}_i$ signaling, MAPK responses, cAMP reductions, and membrane ruffling and impair or abolish sustained signaling from the endosome.

DISCUSSION

Our study, which demonstrates that CaSR sustained signaling can occur by a non-canonical endosomal pathway, in addition to the established canonical PM pathway (Figure 7C), provides an explanation for the observed reduction in CaSR signaling that is paradoxically associated with increased CaSR PM expression because of FHH3-associated AP2σ mutations (Figures 1, 2, 3, and 4) (Nesbit et al., 2013b). Thus, in normal cells, total CaSR signaling comprises the output from the PM immediate and endosomal sustained pathways (Figure 7C); however, in cells with FHH3-associated AP2σ mutations, which impair CaSR internalization (Figure 4), the contribution from the endosomal pathway is lost or markedly reduced, with the remaining CaSR signaling occurring from the PM pathway (Figure 7C). Thus, CaSR endosomal signaling, which is sensitive to the dynamin-blocking agent Dyngo (Figure 6) and to DN mutants of the early endosomal protein Rab5 (Figure 6), occurs via $G\alpha_{q/11}$ (Figures 5 and 6). $G\alpha_{q/11}$ mediates alterations in Ca^{2+} (Figure 1), cAMP (Figure 2), membrane ruffling (Figure 2), and MAPK responses (Figure 1), all of which are impaired in cells expressing FHH3-associated mutations of AP2σ (Figures 1 and 2) that forms part of the heterotetrameric AP2 that plays a critical role in clathrin-mediated endocytosis. This CaSR sustained signaling is also not affected by tunicamycin (Figure S5), indicating a lack of requirement for newly synthesized CaSRs (Grant et al., 2011).

The three FHH3-associated AP2σ-R15 mutants, which all affected CaSR internalization—but not uptake of other clathrin-mediated endocytic cargos, such as transferrin or another GPCR, the β2AR (Figure S1)—had different effects on CaSR endocytosis and consequently different effects on signaling. Critically, these AP2 mutations unveiled that $G\alpha_{q/11}$ signaling was more sensitive to alterations in CaSR endocytosis than the $G\alpha_{i/o}$ pathway. Thus, the AP2σ-C15 mutant delayed CaSR internalization at the CCP (Dyngo sensitive) stage, whereas the AP2σ-H15 and AP2σ-L15 mutants inhibited CaSR internalization at the clathrin-coated vesicle (CCV) (Rab5-DN sensitive) stage. These milder effects of the AP2σ-C15 mutant on CaSR internalization still reduced $G\alpha_{q/11}$ signaling, thereby indicating a possible threshold requirement for receptor occupancy within endosomes for activation of this G-protein pathway. In addition,

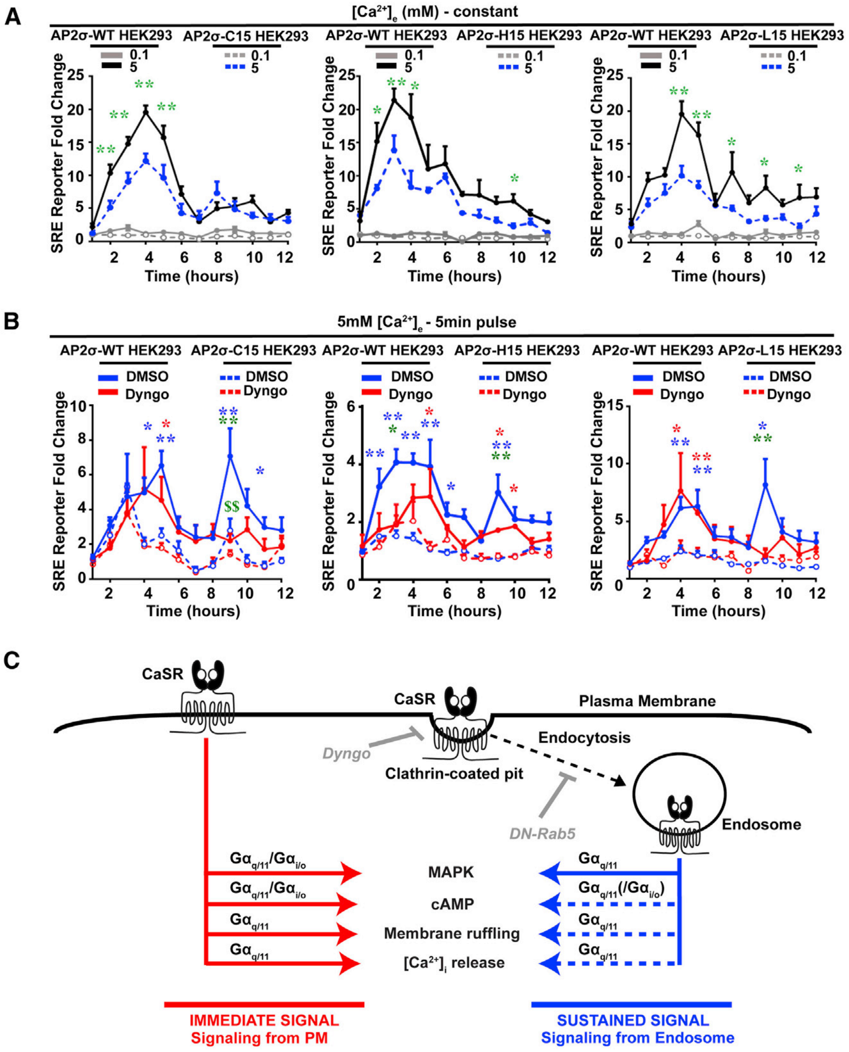

Figure 7. AP2σ-R15 Mutations Impair Sustained Signaling from Endosomes

Studies of sustained signaling using SRE luciferase reporter assays in AP2σ-WT/CaSR-WT and AP2σ mutant/CaSR-WT HEK293 cells.

(A) SRE luciferase reporter responses to constant treatment of 0.1 or 5 mM Ca^{2+}_e. Asterisks indicate p values for WT versus mutant responses (green) (n = 10–12). Statistical comparisons between 0.1 and 5 mM in the same cell type are not shown but were significantly greater for 5 mM in all cells between hours 2 and 11 ($p < 0.05$). Responses to 5 mM Ca^{2+}_e were significantly greater in AP2σ-WT (R15) cells compared to AP2σ mutant (C15, H15, and L15) cells. Data are shown as mean + SEM with *$p < 0.05$, **$p < 0.02$ (two-way ANOVA).

(B) SRE luciferase reporter response to 5 min pulses of 5 mM Ca^{2+}_e treated with DMSO (blue) or Dyngo (red) in AP2σ-WT or AP2σ mutant cells (n = 10–12). Blue and red asterisks indicate WT versus mutant cells treated with DMSO and with Dyngo, respectively, and green asterisks and dollar signs indicate WT DMSO versus WT Dyngo and mutant DMSO versus mutant Dyngo, respectively. Data are shown as mean + SEM with *$p < 0.05$, **$p < 0.02$ or $$p < 0.02 (two-way ANOVA).

(C) Summary of effects of AP2σ-R15 mutations on CaSR signaling pathways. CaSR is able to signal from the PM (red), using the $G\alpha_{q/11}$ and $G\alpha_{i/o}$ pathways to enhance MAPK signaling and to reduce cAMP, and increase membrane ruffling and Ca^{2+}_i release, using $G\alpha_{q/11}$. Following activation, CaSR is clustered into CCPs, before vesicle scission and internalization in clathrin-coated vesicles, and then into endosomes. Our results show that CaSR can induce sustained MAPK signaling (blue) from Rab5 endosomes and that FHH3-associated AP2σ mutations (C15, H15, and L15) impair all immediate signaling pathways (red) and impair or abolish sustained $G\alpha_{q/11}$ signaling from the endosome, with responses of MAPK shown as a solid blue line (Figures 6 and 7) and other likely responses shown as a broken blue line and in parentheses. Pit invagination can be blocked by Dyngo, and maturation to Rab5-positive vesicles can be blocked by DN Rab5 mutant.

the AP2σ-C15 mutant, but not AP2σ-L15 or AP2σ-H15, significantly affected $G\alpha_{i/o}$ signaling at high $[Ca^{2+}]_e$, i.e., 10 mM (Figure 2), thereby suggesting that CaSR-mediated $G\alpha_{i/o}$ signaling at high $[Ca^{2+}]_e$ is regulated at the CCPs, as opposed to Rab5 endosomes. Furthermore, $G\alpha_{i/o}$, which can enhance MAPK signaling (Kifor et al., 2001), does not contribute to the sustained signal (Figures 6L and 6M), demonstrating the stronger requirement of receptor endocytosis for $G\alpha_{q/11}$ signaling. In contrast, the AP2σ-L15 mutant, which had impaired CaSR internalization and abolished $G\alpha_{q/11}$-mediated sustained MAPK signaling, resulting in the most severely reduced $G\alpha_{q/11}$ signaling, had markedly reduced ADIS responses (Figure 4). These findings indicate not only that endosomal $G\alpha_{q/11}$ signaling is critical for ADIS (Figures 4, 5, and 6) but also that there is a link between CaSR trafficking and signaling, thereby providing support for the proposed communication between endosomal compartments and the secretory machinery that links GPCR trafficking to maintain membrane receptor functionality (Clague and Urbé, 2001). Finally, the regulation of CaSR sustained signaling via its local environment within the endosome has yet to be established. Studies of the effect of different ligands, pH, receptor density, and tissue-specific differences that have previously been recognized for the CaSR (Conigrave and Ward, 2013; Quinn et al., 2004) require further investigation within the sustained signal context.

Our results reveal that the CaSR, a class C GPCR, induces sustained endosomal signaling (Figures 5, 6, and 7). This has similarities to reports for class A GPCRs, such as β2AR and LHR, which do not require βarrestin for endosomal and/or MAPK sustained signals (Irannejad et al., 2013; Jean-Alphonse et al., 2014). Moreover, GPCRs that use non-canonical signals often do so to facilitate biased agonism. This is illustrated by the class A GPCR V2R, which elicits sustained endosomal signals with vasopressin but rapid signals with oxytocin (Feinstein

et al., 2013), and the class B PTH1R, which has sustained signals for PTH but rapid signals for PTH-related peptide (Ferrandon et al., 2009). Such spatial control of GPCR signaling has emerged as an important mechanism by which cells translate complex information into distinct cellular responses using a finite number of signal proteins. This is particularly the case for the CaSR, which has wide-ranging functions in diverse cell types, is able to couple to multiple G proteins, and responds to a variety of ligands. Thus, the ability to use immediate and sustained signaling pathways could account for some tissue- and cell-specific functions of the CaSR. For example, an immediate signaling pathway would likely facilitate the CaSR to rapidly respond to changes in $[Ca^{2+}]_e$ to restore calcium homeostasis by parathyroid and renal cells. In contrast, the role of CaSR in fetal development and bone mineralization (Goltzman and Hendy, 2015; Riccardi et al., 2013), which may require long-acting signals, may be facilitated by a sustained signaling pathway, providing a mechanism for the functional diversity of the CaSR.

In conclusion, our studies have demonstrated that the CaSR, a class C GPCR, mediates a sustained signal from an internal location that is likely to be the endosomes. In addition, our systematic characterization of CaSR signaling by such non-canonical, internalization-dependent (e.g., endosomal) pathways provides a paradigm for understanding how pleiotropic signaling pathways activated by a single GPCR can be resolved via spatially directed G-protein selectivity.

EXPERIMENTAL PROCEDURES

Detailed methods and information on constructs, oligonucleotides, and antibodies can be found in the Supplemental Experimental Procedures.

Ethics Statement

Informed consent was obtained from individuals using protocols approved by local and national ethics committees, London, UK (MREC/02/2/93).

Cell Culture

HEK-CaSR have been described (Nesbit et al., 2013b). HEK293 cells stably expressing AP2σ WT or mutant proteins were generated using a pcDNA3.1 construct (Invitrogen) containing full-length AP2σ cDNA with silent mutations to protect against AP2σ siRNA (Santa Cruz Biotechnology). Clonal cells were generated as described (Nesbit et al., 2013b), and cells with deletion of $G\alpha_q$, $G\alpha_{11}$, $G\alpha_{12}$, $G\alpha_{13}$, βarrestin-1, and βarrestin-2 by CRISPR-Cas have been described (Devost et al., 2017). Epstein-Barr virus-transformed lymphoblastoid cells were generated from members of the FHH3 kindred as described (Parkinson and Thakker, 1992). Transfections were performed with Lipofectamine 2000 (Invitrogen). Mutations within constructs were introduced by site-directed mutagenesis using Quikchange Lightning XL or Multi kits (Agilent Technologies) and confirmed by sequencing as described (Newey et al., 2013).

Western Blot

For sustained signaling studies, cells were stimulated with 5 mM $CaCl_2$ for 5 min, followed by incubation in 0 mM $CaCl_2$ for 0–60 min. For studies with 30 μM Dyngo-4a (Abcam) (Jean-Alphonse et al., 2014), cells were pre-incubated for 30 min. For studies with 5 μM U73122 (Sigma), 1 μM GFX (Sigma), 100 μM 2-APB (Sigma), or 5 μg/mL tunicamycin (Sigma), compounds were added to the media and cells were incubated after calcium stimulation. For studies of Rab5 contribution to sustained signaling, 100 ng/mL mCh-Rab5-WT (Addgene plasmid 49201), mCh-Rab5 dominant negative (DN; S34N) or mCh-Rab5 CA (Q79L), were transfected 48 hr before western blot analysis. Western blots for pERK1/2 were then performed as described (Gorvin et al., 2017).

Functional Assays

Transferrin assays were performed as described (Gorvin et al., 2013). IP_1 assays were performed according to manufacturer's instructions. For pERK1/2 AlphaScreen assays, cells were transfected with pEGFP-CaSR and treated with 0–10 mM $CaCl_2$ for 5 min. For cAMP assays, cells were pre-treated with forskolin for 30 min. For inhibitor studies, cells were pre-treated with 300 ng/mL PTx or vehicle (ethanol) for 6 hr, 1 μM UBO-QIC or vehicle (DMSO) for 2 hr, or 15 μM gallein or vehicle (DMSO) for 15 min (Grant et al., 2011). AlphaScreen assays were performed as previously described (Gorvin et al., 2017). Apoptosis and proliferation were assessed using Caspase-Glo 3/7 and CellTiter Blue kits, respectively (Promega). For luciferase reporter assays, cells were transfected with pEGFP-CaSR, a reporter construct (pGL4-NFAT, pGL4-SRE, or pGL4-SRF), and a renilla construct (pRL) as described (Gorvin et al., 2017). Cells were treated with 0–10 mM $CaCl_2$ for 4 hr. For sustained signaling studies, HEK-CaSR cells were transfected with luciferase construct and pRL and given one of four treatments: (1) 0.1 mM $CaCl_2$, (2) 5 mM $CaCl_2$ for the whole experiment (constant), (3) 5 min pulse of 5 mM $CaCl_2$ followed by 0.1 mM $CaCl_2$ with vehicle (DMSO) for the duration of the experiment, or (4) 5 min pulse of 5 mM $CaCl_2$ followed by 0.1 mM $CaCl_2$ with 30 μM Dyngo-4a for the duration of the experiment. Cells were pre-incubated with 1 μM UBO-QIC or DMSO for 2 hr or 10 μM forskolin (MP Biomedicals) and 300 ng/mL PTx (Sigma) or vehicle (ethanol diluent) for 6 hr (Avlani et al., 2013). Luciferase assays and Caspase-Glo 3/7 were measured on a Veritas luminometer (Promega), and CellTiter Blue was measured on a CytoFluor microplate reader (PerSeptive Biosystems).

Fluorescent Imaging

For membrane ruffling, cells were transfected with pEGFP-CaSR, and actin was visualized with Phalloidin-594 (Molecular Probes) following treatment with 0, 5, and 10 mM $Ca^{2+}{}_e$. Cells were imaged on a Nikon Eclipse E400 wide-field microscope using adapted protocols (Bouschet et al., 2007; Davey et al., 2012). Single-cell microfluorimetry experiments were performed in AP2σ-WT or mutant cells transiently transfected with pEGFP-CaSR. Cells were loaded with Fura-2 (Molecular Probes) for 30 min and imaged on a Nikon TE2000 inverted microscope. Cells were perfused with extracellular bath solution with increasing $CaCl_2$ concentrations. Fura-2 images were acquired using 340/380 nm excitation and 510 nm emission on μManager software (NIH). Methods for TIRF microscopy were adapted from previous studies (Grant et al., 2011; Hoppa et al., 2009). Images were obtained with an Olympus IX-81 TIRF microscope. To monitor CaSR internalization, cells were pre-incubated with BTx-594 and then perfused with 0.1 or 10 mM $CaCl_2$ imaging solution. Images were captured at 10 frames/s in BSEP studies and 3 frames/s for clathrin studies. Images were acquired using Cell^R software (Olympus). Confocal imaging was performed in HEK293 cells using methods adapted from previous studies (Bouschet et al., 2007; Hanyaloglu et al., 2005). Images were captured using a confocal, laser-scanning microscope (Leica SP5). All images were analyzed using ImageJ (NIH).

Statistical Analysis

Two-tailed unpaired t test, two-way ANOVA, χ^2 test, Mann-Whitney U test, Pearson's correlation coefficient, and F test were used to calculate statistical significance using GraphPad Prism 6 software. A $p < 0.05$ was considered statistically significant. Statistical tests used are indicated in the methods in the Supplemental Experimental Procedures and figure legends.

SUPPLEMENTAL INFORMATION

Supplemental Information includes Supplemental Experimental Procedures, seven figures, and two tables and can be found with this article online at https://doi.org/10.1016/j.celrep.2017.12.089.

ACKNOWLEDGMENTS

This work was supported by grants from the Medical Research Council (G1000467 to C.M.G. and R.V.T.), National Institute for Health Research

(NIHR) Oxford Biomedical Research Centre (to R.V.T.), Shriners Hospitals for Children (to M.P.W.), and a Goodger and Schorstein Scholarship from the Radcliffe Department of Medicine, University of Oxford (to C.M.G.). R.V.T. has Senior Investigator Awards from the Wellcome Trust (106995/z/15/z) and NIHR (NF-SI-0514-10091). A.R. was a Wellcome Trust Clinical Training Fellow.

AUTHOR CONTRIBUTIONS

C.M.G., B.H., A.I.T., A.C.H., and R.V.T. designed experiments; M.P.W., A.C.H., G.E.B., P.R., and R.V.T. provided materials; C.M.G., A.R., M.F., S.S., and A.C.H. performed experiments and analyzed data; C.M.G., A.C.H., and R.V.T. wrote the manuscript; C.M.G., B.H., A.I.T., A.R., M.F., S.S., A.I., M.P.W., A.C.H., G.E.B., and R.V.T. reviewed and edited the manuscript.

DECLARATION OF INTERESTS

The authors declare no competing interests.

Received: June 16, 2017
Revised: November 30, 2017
Accepted: December 22, 2017
Published: January 23, 2018

REFERENCES

Avlani, V.A., Ma, W., Mun, H.C., Leach, K., Delbridge, L., Christopoulos, A., and Conigrave, A.D. (2013). Calcium-sensing receptor-dependent activation of CREB phosphorylation in HEK293 cells and human parathyroid cells. Am. J. Physiol. Endocrinol. Metab. *304*, E1097–E1104.

Bouschet, T., Martin, S., Kanamarlapudi, V., Mundell, S., and Henley, J.M. (2007). The calcium-sensing receptor changes cell shape via a beta-arrestin-1 ARNO ARF6 ELMO protein network. J. Cell Sci. *120*, 2489–2497.

Calebiro, D., Nikolaev, V.O., Gagliani, M.C., de Filippis, T., Dees, C., Tacchetti, C., Persani, L., and Lohse, M.J. (2009). Persistent cAMP-signals triggered by internalized G-protein-coupled receptors. PLoS Biol. *7*, e1000172.

Chakravarti, B., Chattopadhyay, N., and Brown, E.M. (2012). Signaling through the extracellular calcium-sensing receptor (CaSR). In Advances in Experimental Medicine and Biology, C. Signaling and M.S. Islam, eds. (Springer), pp. 103–142.

Clague, M.J., and Urbé, S. (2001). The interface of receptor trafficking and signalling. J. Cell Sci. *114*, 3075–3081.

Conigrave, A.D., and Ward, D.T. (2013). Calcium-sensing receptor (CaSR): pharmacological properties and signaling pathways. Best Pract. Res. Clin. Endocrinol. Metab. *27*, 315–331.

Davey, A.E., Leach, K., Valant, C., Conigrave, A.D., Sexton, P.M., and Christopoulos, A. (2012). Positive and negative allosteric modulators promote biased signaling at the calcium-sensing receptor. Endocrinology *153*, 1232–1241.

Davies, S.L., Gibbons, C.E., Vizard, T., and Ward, D.T. (2006). Ca2+-sensing receptor induces Rho kinase-mediated actin stress fiber assembly and altered cell morphology, but not in response to aromatic amino acids. Am. J. Physiol. Cell Physiol. *290*, C1543–C1551.

Devost, D., Sleno, R., Pétrin, D., Zhang, A., Shinjo, Y., Okde, R., Aoki, J., Inoue, A., and Hébert, T.E. (2017). Conformational profiling of the AT1 angiotensin II receptor reflects biased agonism, G protein coupling, and cellular context. J. Biol. Chem. *292*, 5443–5456.

Feinstein, T.N., Yui, N., Webber, M.J., Wehbi, V.L., Stevenson, H.P., King, J.D., Jr., Hallows, K.R., Brown, D., Bouley, R., and Vilardaga, J.P. (2013). Noncanonical control of vasopressin receptor type 2 signaling by retromer and arrestin. J. Biol. Chem. *288*, 27849–27860.

Ferrandon, S., Feinstein, T.N., Castro, M., Wang, B., Bouley, R., Potts, J.T., Gardella, T.J., and Vilardaga, J.P. (2009). Sustained cyclic AMP production by parathyroid hormone receptor endocytosis. Nat. Chem. Biol. *5*, 734–742.

Galperin, E., and Sorkin, A. (2003). Visualization of Rab5 activity in living cells by FRET microscopy and influence of plasma-membrane-targeted Rab5 on clathrin-dependent endocytosis. J. Cell Sci. *116*, 4799–4810.

Gao, Z.G., and Jacobson, K.A. (2016). On the selectivity of the Gαq inhibitor UBO-QIC: a comparison with the Gαi inhibitor pertussis toxin. Biochem. Pharmacol. *107*, 59–66.

Goltzman, D., and Hendy, G.N. (2015). The calcium-sensing receptor in bone—mechanistic and therapeutic insights. Nat. Rev. Endocrinol. *11*, 298–307.

Gorvin, C.M., Wilmer, M.J., Piret, S.E., Harding, B., van den Heuvel, L.P., Wrong, O., Jat, P.S., Lippiat, J.D., Levtchenko, E.N., and Thakker, R.V. (2013). Receptor-mediated endocytosis and endosomal acidification is impaired in proximal tubule epithelial cells of Dent disease patients. Proc. Natl. Acad. Sci. USA *110*, 7014–7019.

Gorvin, C.M., Hannan, F.M., Howles, S.A., Babinsky, V.N., Piret, S.E., Rogers, A., Freidin, A.J., Stewart, M., Paudyal, A., Hough, T.A., et al. (2017). Gα11 mutation in mice causes hypocalcemia rectifiable by calcilytic therapy. JCI Insight *2*, e91103.

Grant, M.P., Stepanchick, A., Cavanaugh, A., and Breitwieser, G.E. (2011). Agonist-driven maturation and plasma membrane insertion of calcium-sensing receptors dynamically control signal amplitude. Sci. Signal. *4*, ra78.

Hannan, F.M., Babinsky, V.N., and Thakker, R.V. (2016). Disorders of the calcium-sensing receptor and partner proteins: insights into the molecular basis of calcium homeostasis. J. Mol. Endocrinol. *57*, R127–R142.

Hanyaloglu, A.C., McCullagh, E., and von Zastrow, M. (2005). Essential role of Hrs in a recycling mechanism mediating functional resensitization of cell signaling. EMBO J. *24*, 2265–2283.

Hofer, A.M., Curci, S., Doble, M.A., Brown, E.M., and Soybel, D.I. (2000). Intercellular communication mediated by the extracellular calcium-sensing receptor. Nat. Cell Biol. *2*, 392–398.

Holstein, D.M., Berg, K.A., Leeb-Lundberg, L.M., Olson, M.S., and Saunders, C. (2004). Calcium-sensing receptor-mediated ERK1/2 activation requires Galphai2 coupling and dynamin-independent receptor internalization. J. Biol. Chem. *279*, 10060–10069.

Hoppa, M.B., Collins, S., Ramracheya, R., Hodson, L., Amisten, S., Zhang, Q., Johnson, P., Ashcroft, F.M., and Rorsman, P. (2009). Chronic palmitate exposure inhibits insulin secretion by dissociation of Ca(2+) channels from secretory granules. Cell Metab. *10*, 455–465.

Huang, C., Hujer, K.M., Wu, Z., and Miller, R.T. (2004). The Ca2+-sensing receptor couples to Galpha12/13 to activate phospholipase D in Madin-Darby canine kidney cells. Am. J. Physiol. Cell Physiol. *286*, C22–C30.

Irannejad, R., Tomshine, J.C., Tomshine, J.R., Chevalier, M., Mahoney, J.P., Steyaert, J., Rasmussen, S.G., Sunahara, R.K., El-Samad, H., Huang, B., and von Zastrow, M. (2013). Conformational biosensors reveal GPCR signalling from endosomes. Nature *495*, 534–538.

Jean-Alphonse, F., Bowersox, S., Chen, S., Beard, G., Puthenveedu, M.A., and Hanyaloglu, A.C. (2014). Spatially restricted G protein-coupled receptor activity via divergent endocytic compartments. J. Biol. Chem. *289*, 3960–3977.

Katritch, V., Cherezov, V., and Stevens, R.C. (2013). Structure-function of the G protein-coupled receptor superfamily. Annu. Rev. Pharmacol. Toxicol. *53*, 531–556.

Kelly, B.T., McCoy, A.J., Späte, K., Miller, S.E., Evans, P.R., Höning, S., and Owen, D.J. (2008). A structural explanation for the binding of endocytic dileucine motifs by the AP2 complex. Nature *456*, 976–979.

Kifor, O., MacLeod, R.J., Diaz, R., Bai, M., Yamaguchi, T., Yao, T., Kifor, I., and Brown, E.M. (2001). Regulation of MAP kinase by calcium-sensing receptor in bovine parathyroid and CaR-transfected HEK293 cells. Am. J. Physiol. Renal Physiol. *280*, F291–F302.

Kotowski, S.J., Hopf, F.W., Seif, T., Bonci, A., and von Zastrow, M. (2011). Endocytosis promotes rapid dopaminergic signaling. Neuron *71*, 278–290.

Mamillapalli, R., VanHouten, J., Zawalich, W., and Wysolmerski, J. (2008). Switching of G-protein usage by the calcium-sensing receptor reverses its

effect on parathyroid hormone-related protein secretion in normal versus malignant breast cells. J. Biol. Chem. *283*, 24435–24447.

Nesbit, M.A., Hannan, F.M., Howles, S.A., Babinsky, V.N., Head, R.A., Cranston, T., Rust, N., Hobbs, M.R., Heath, H., 3rd, and Thakker, R.V. (2013a). Mutations affecting G-protein subunit α11 in hypercalcemia and hypocalcemia. N. Engl. J. Med. *368*, 2476–2486.

Nesbit, M.A., Hannan, F.M., Howles, S.A., Reed, A.A., Cranston, T., Thakker, C.E., Gregory, L., Rimmer, A.J., Rust, N., Graham, U., et al. (2013b). Mutations in AP2S1 cause familial hypocalciuric hypercalcemia type 3. Nat. Genet. *45*, 93–97.

Newey, P.J., Gorvin, C.M., Cleland, S.J., Willberg, C.B., Bridge, M., Azharuddin, M., Drummond, R.S., van der Merwe, P.A., Klenerman, P., Bountra, C., and Thakker, R.V. (2013). Mutant prolactin receptor and familial hyperprolactinemia. N. Engl. J. Med. *369*, 2012–2020.

Parkinson, D.B., and Thakker, R.V. (1992). A donor splice site mutation in the parathyroid hormone gene is associated with autosomal recessive hypoparathyroidism. Nat. Genet. *1*, 149–152.

Pi, M., Spurney, R.F., Tu, Q., Hinson, T., and Quarles, L.D. (2002). Calcium-sensing receptor activation of rho involves filamin and rho-guanine nucleotide exchange factor. Endocrinology *143*, 3830–3838.

Quinn, S.J., Bai, M., and Brown, E.M. (2004). pH sensing by the calcium-sensing receptor. J. Biol. Chem. *279*, 37241–37249.

Rappoport, J.Z., and Simon, S.M. (2003). Real-time analysis of clathrin-mediated endocytosis during cell migration. J. Cell Sci. *116*, 847–855.

Riccardi, D., Brennan, S.C., and Chang, W. (2013). The extracellular calcium-sensing receptor, CaSR, in fetal development. Best Pract. Res. Clin. Endocrinol. Metab. *27*, 443–453.

Rosenbaum, D.M., Rasmussen, S.G., and Kobilka, B.K. (2009). The structure and function of G-protein-coupled receptors. Nature *459*, 356–363.

Rossol, M., Pierer, M., Raulien, N., Quandt, D., Meusch, U., Rothe, K., Schubert, K., Schöneberg, T., Schaefer, M., Krügel, U., et al. (2012). Extracellular Ca2+ is a danger signal activating the NLRP3 inflammasome through G protein-coupled calcium sensing receptors. Nat. Commun. *3*, 1329.

Stauffer, T.P., Ahn, S., and Meyer, T. (1998). Receptor-induced transient reduction in plasma membrane PtdIns(4,5)P2 concentration monitored in living cells. Curr. Biol. *8*, 343–346.

Stenmark, H., Parton, R.G., Steele-Mortimer, O., Lütcke, A., Gruenberg, J., and Zerial, M. (1994). Inhibition of rab5 GTPase activity stimulates membrane fusion in endocytosis. EMBO J. *13*, 1287–1296.

Tojkander, S., Gateva, G., and Lappalainen, P. (2012). Actin stress fibers—assembly, dynamics and biological roles. J. Cell Sci. *125*, 1855–1864.

Wehbi, V.L., Stevenson, H.P., Feinstein, T.N., Calero, G., Romero, G., and Vilardaga, J.P. (2013). Noncanonical GPCR signaling arising from a PTH receptor-arrestin-G$\beta\gamma$ complex. Proc. Natl. Acad. Sci. USA *110*, 1530–1535.

Yarova, P.L., Stewart, A.L., Sathish, V., Britt, R.D., Jr., Thompson, M.A., Lowe, A.P.P., Freeman, M., Aravamudan, B., Kita, H., Brennan, S.C., et al. (2015). Calcium-sensing receptor antagonists abrogate airway hyperresponsiveness and inflammation in allergic asthma. Sci. Transl. Med. *7*, 284ra260.

Zietek, T., and Daniel, H. (2015). Intestinal nutrient sensing and blood glucose control. Curr. Opin. Clin. Nutr. Metab. Care *18*, 381–388.

Cell Reports, Volume 22

Supplemental Information

AP2σ Mutations Impair Calcium-Sensing Receptor Trafficking and Signaling, and Show an Endosomal Pathway to Spatially Direct G-Protein Selectivity

Caroline M. Gorvin, Angela Rogers, Benoit Hastoy, Andrei I. Tarasov, Morten Frost, Silvia Sposini, Asuka Inoue, Michael P. Whyte, Patrik Rorsman, Aylin C. Hanyaloglu, Gerda E. Breitwieser, and Rajesh V. Thakker

SUPPLEMENTAL INFORMATION

AP2σ mutations impair calcium-sensing receptor trafficking and signaling, and reveal an endosomal pathway that spatially-directs G-protein selectivity

Caroline M Gorvin[1], Angela Rogers[1], Benoit Hastoy[2], Andrei I Tarasov[2], Morten Frost[1], Silvia Sposini[3], Asuka Inoue[4,5], Michael P Whyte[6], Patrik Rorsman[2], Aylin C Hanyaloglu[3], Gerda E Breitwieser[7], Rajesh V Thakker[1]

Supplemental Figures

FIGURE S1 Development of HEK293 AP2σ Stable Cell-lines, Related to Figure 1

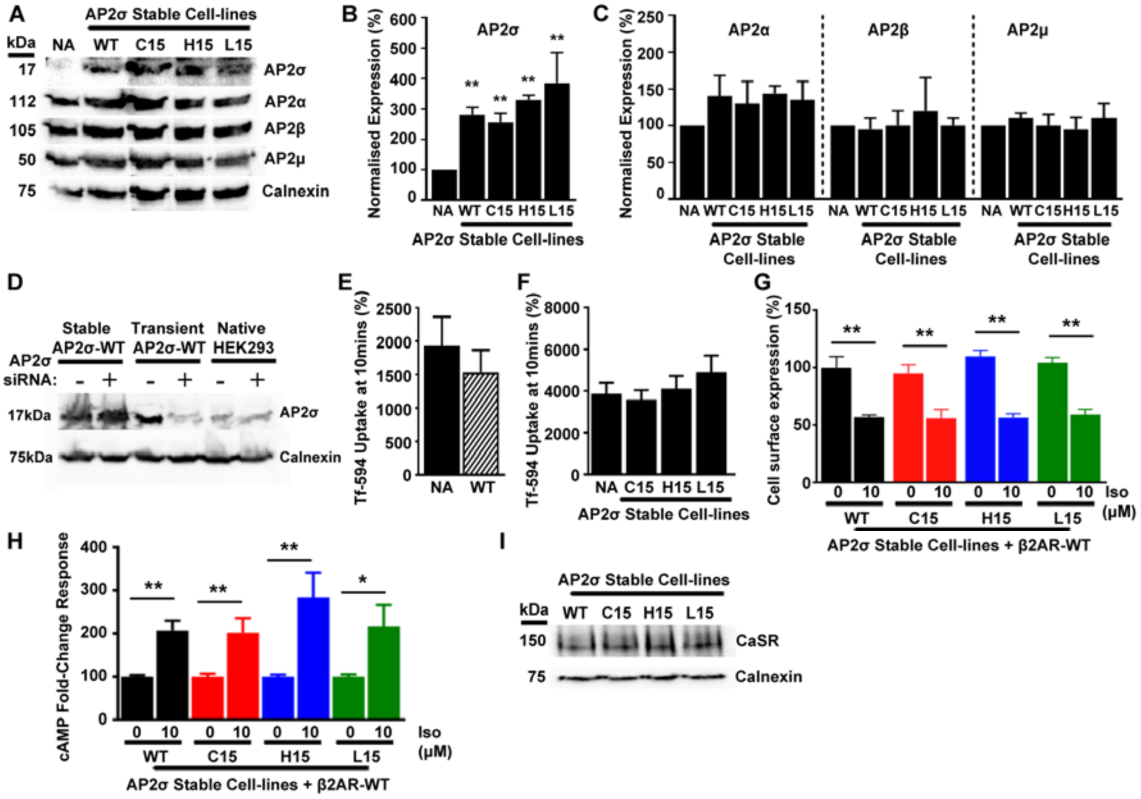

Stable overexpression of AP2σ protein (wild-type (WT, R15) or mutant (C15, H15, L15)) in HEK293 cells compared to parental native (NA) cells was confirmed by (A) Western blot analysis and (B) densitometric analysis from 4 independent blots. Data are shown as mean+SEM. $^{**}p<0.02$ (2-way ANOVA); NA cells vs. test cells. Note that the Western blots detecting AP2σ were exposed for 2 seconds, whereas those for AP2α, AP2β and AP2μ were exposed for 1 minute. This is because the expression of AP2σ in the cells stably expressing AP2σ was ~200% greater than that of the other subunits and of AP2σ in the NA cells, which appear therefore to have a low expression. Longer exposure of these AP2σ Western blots would reveal the expression of AP2σ in NA cells, but would lead to dense (over-exposed) bands in the AP2σ stably expressing cells that would not have allowed any meaningful quantification by densitometry. (C) Expression of AP2 subunits α, β and μ, was unaffected by AP2σ overexpression. These findings are in contrast to other studies in which deletion of one AP2 subunit affected the expression and stability of other AP2 components (Boucrot et al., 2010; Mitsunari et al., 2005), but are consistent with our studies of mice with a heterozygous N-ethyl-N-nitrosourea-induced splice-site mutation, which results in loss of 17 amino acids from AP2σ, and a ~50% reduction in AP2σ protein, in which the expression of AP2 α, β and μ are similar to wild-type mice (Gorvin et al., 2017b). Data are from Western blot densitometry analysis of 4 independent blots and are shown as mean+SEM with 2-way ANOVA analysis. Calnexin was used as a housekeeping protein. (D) Western blot analysis confirmed that siRNA to AP2σ is able to selectively knockdown AP2σ expression in native HEK293 cells and in native HEK293 cells transiently expressing AP2σ-WT (Transient), but not in AP2σ stable cell-lines. Calnexin was used as a housekeeping protein. (E-F) Transferrin (Tf) uptake following 10 minutes of Tf-594 treatment in (E) Native (NA) HEK293 cells stably expressing AP2σ-WT protein (WT, R15), and (F) HEK293 cells stably expressing AP2σ-WT (R15) or AP2σ-mutant proteins (C15, H15 or L15). Data is presented as a percentage compared to uptake at 0 minutes. Uptake of fluorescent transferrin is not affected by overexpression of the AP2σ protein or by AP2σ mutation. Data are from n=8 biological replicates with two-way ANOVA analysis. (G) Cell surface expression of FLAG-β2-adrenergic receptor (β2AR) measured by ELISA, and (H) cAMP signaling by β2AR in response to 0μM or 10μM isoproterenol, assessed in AP2σ-WT or AP2σ-mutant cell-lines. Treatment with Iso led to reduced cell surface expression and increased cAMP responses in all AP2σ-WT and AP2σ-mutant cells, and no significant differences were seen between responses when comparing AP2σ-WT and AP2σ-mutant cells. Data shows mean+SEM and represents data from n=6-12

biological replicates; **$p<0.02$ for 0 vs 10 μM (2-way ANOVA). (I) Western blot analysis confirmed that the pEGFP-CaSR-WT construct expressed CaSR-WT at similar levels when transfected into AP2σ-WT or AP2σ-mutant cells.

FIGURE S2 $Ca^{2+}{}_e$-induced $Ca^{2+}{}_i$ Signaling is Impaired in AP2σ-Mutant Cells Transiently Expressing CaSR due to Delayed Oscillation Events, Related to Figure 1

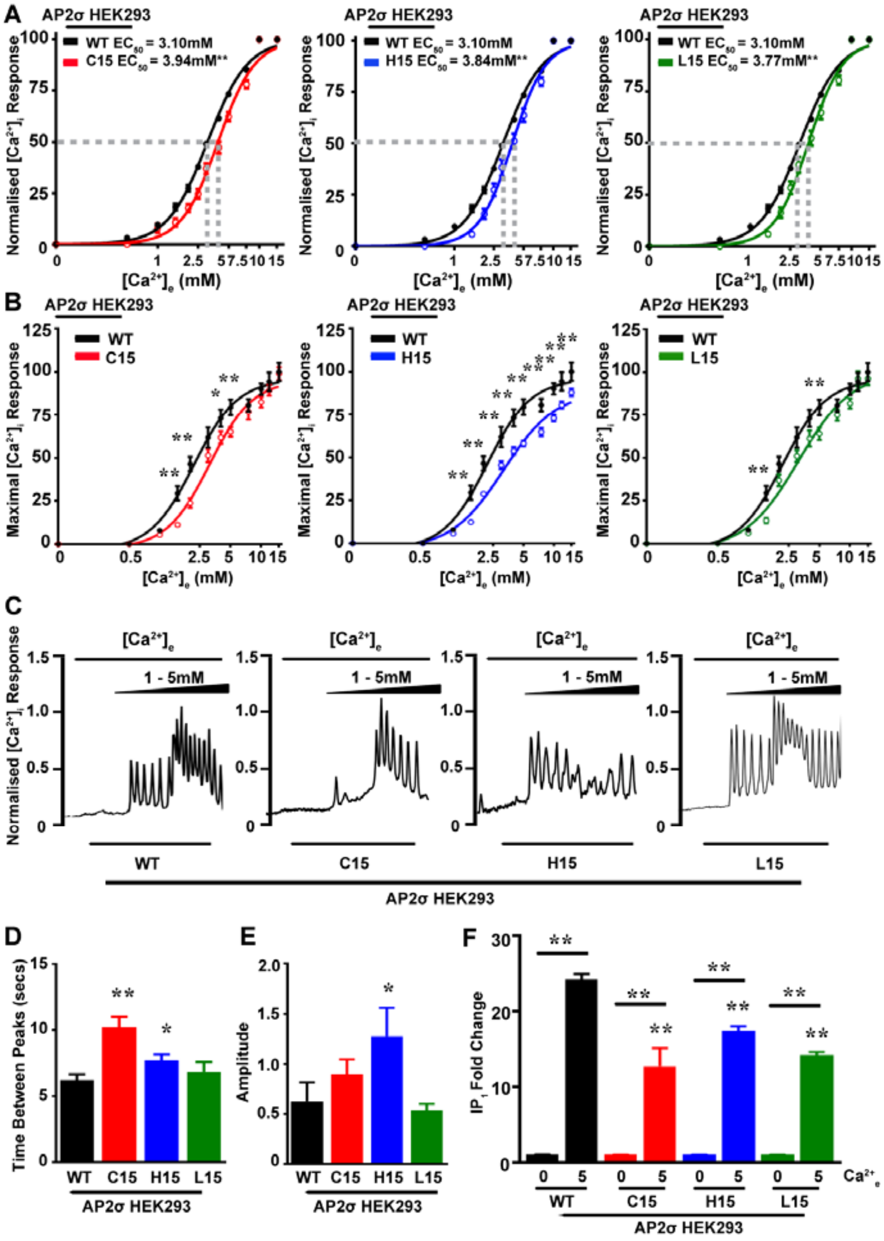

(A) Normalized Fura-2 ratios in response to increasing doses of $Ca^{2+}{}_e$ in single cells expressing AP2σ-WT (R15) or mutant (C15, H15 or L15) proteins and transiently transfected with pEGFP-CaSR. Data are shown as mean+95% confidence intervals (CI), n=36-50 cells from 9-10 transfections. $Ca^{2+}{}_e$-induced $Ca^{2+}{}_i$ responses are rightward-shifted in mutants resulting in higher half-maximal $Ca^{2+}{}_i$ responses (EC50s) (dashed grey line) when compared to wild-type cells. EC_{50} values (WT, 3.10mM (95% confidence interval (CI) 3.05-3.16), C15, 3.94mM (95% CI 3.81-4.06), H15, 3.84mM (95% CI 3.70-3.98), L15, 3.77mM (3.61-3.91), p<0.02 for all). **p<0.02 mutant vs WT (*F*-test). (B) Maximal $Ca^{2+}{}_e$-induced (Emax) $Ca^{2+}{}_i$ responses. Emax values were significantly lower at $[Ca^{2+}{}_e]$ in the range 2-10mM in AP2σ-H15 cells, and in the range 2-5mM in AP2σ-C15 and AP2σ-L15 cells. The maximal signaling response of a GPCR is influenced by its ability to couple to its cognate G-protein, and thus it is possible that the AP2σ-H15 mutant impairs coupling and/or dissociation of $G\alpha_{q/11}$ from the CaSR more than the AP2σ-C15 and –L15 mutants. Data is expressed as mean±SEM. **p<0.02 vs WT. (C) Representative images of calcium oscillations observed in AP2σ-WT (R15) and AP2σ-mutant (C15, H15, L15) cells. (D) Frequency of oscillations was reduced in AP2σ-C15 and –H15 cells, while (E) amplitude was only affected in

AP2σ-H15 cells. (F) Accumulation of the IP_3 breakdown product IP_1 in AP2σ-WT/CaSR-WT and AP2σ-mutant/CaSR-WT HEK293 cells in response to 0mM or 5mM Ca^{2+}_e. AP2σ-mutant cells had impaired responses compared to AP2σ-WT cells. Panels (B-D) data are shown as mean+SEM and are from n = 36-50 cells from 9-10 independent transfections; *p<0.05 and **p<0.02 for mutant vs. WT (Mann-Whitney U-test (panel B) and 2-way ANOVA (panels D and E)). Panel F shows mean+SEM and represents data from n=4 biological replicates; **p<0.02 for mutant vs. WT (2-way ANOVA).

FIGURE S3 Normal Expression of AP2 Subunits in FHH3 Patients and their Unaffected (Normal Control) Relatives, Related to Figure 1 and 2

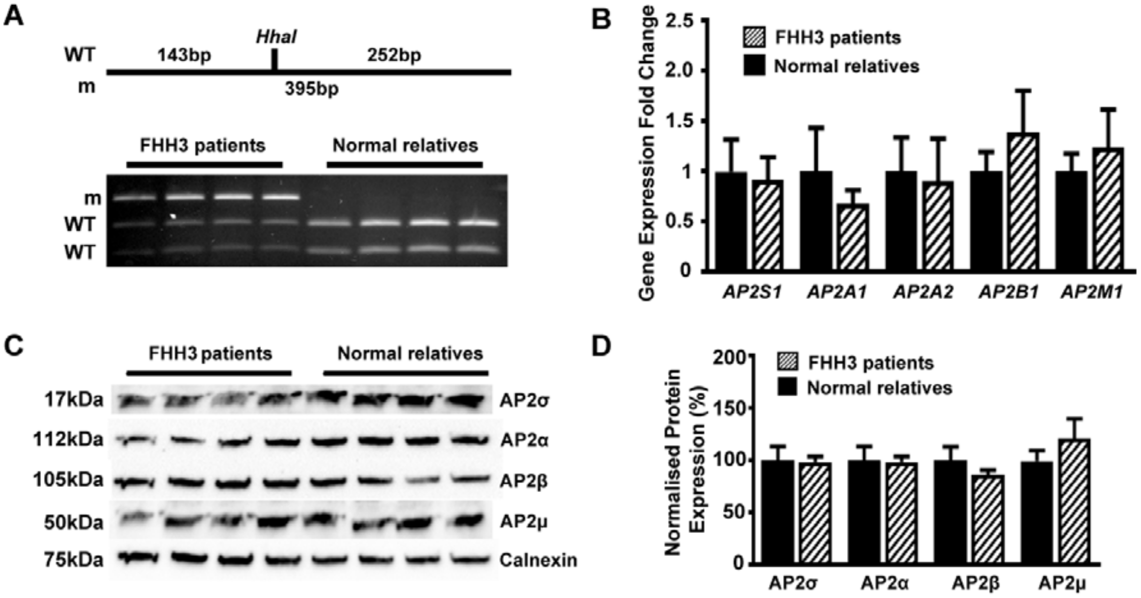

Confirmation of the AP2σ mutation (R15C) in EBV-transformed lymphoblastoid cells from patients of a previously described FHH3 kindred (McMurtry et al., 1992; Nesbit et al., 2013). (A) Restriction endonuclease map showing the cleavage site of *HhaI* that is disrupted by the mutation. Thus, the full-length WT is cleaved once with *HhaI* to yield two products at 143bp and 252bp. The *HhaI* site is lost in AP2σ-mutants (m). Lower panel shows the restriction endonuclease digests of DNA from PCR products of *AP2S1* exon 2, which shows that patients with FHH3, who are heterozygous for the AP2σ R15C mutation, have a mutant (m) uncleaved (395bp) and wild-type (WT) cleaved products (143bp and 252bp); whereas normal relatives are homozygous for the cleaved WT products only. (B) qRT-PCR analysis of genes (*AP2S1*, *AP2A1*, *AP2A2, AP2B1,* and *AP2M1*) which encode the AP2 σ, α, β, and μ subunits, respectively in EBV-transformed lymphoblastoid cells from FHH3 patients and normal relatives. Presence of the AP2σ mutation has no effect on expression levels of any of the subunits. Data is expressed as mean+SEM (n=4). (C) Western blot analysis of the AP2 subunits in protein lysates extracted from EBV-transformed lymphoblastoids of the FHH3 patients and normal (control) relatives, and (D) densitometric analyses of Western blots (n=4). Calnexin was used as a housekeeping protein. The expression of the AP2 subunits was not significantly different in the cells of patients and normal relatives. Expressed as mean+SEM (n=4). Statistical analyses were performed by 2-way ANOVA and student's t-test.

Figure S4 **Membrane Ruffling is Impaired in AP2σ-Mutant Cells, Related to Figure 3**

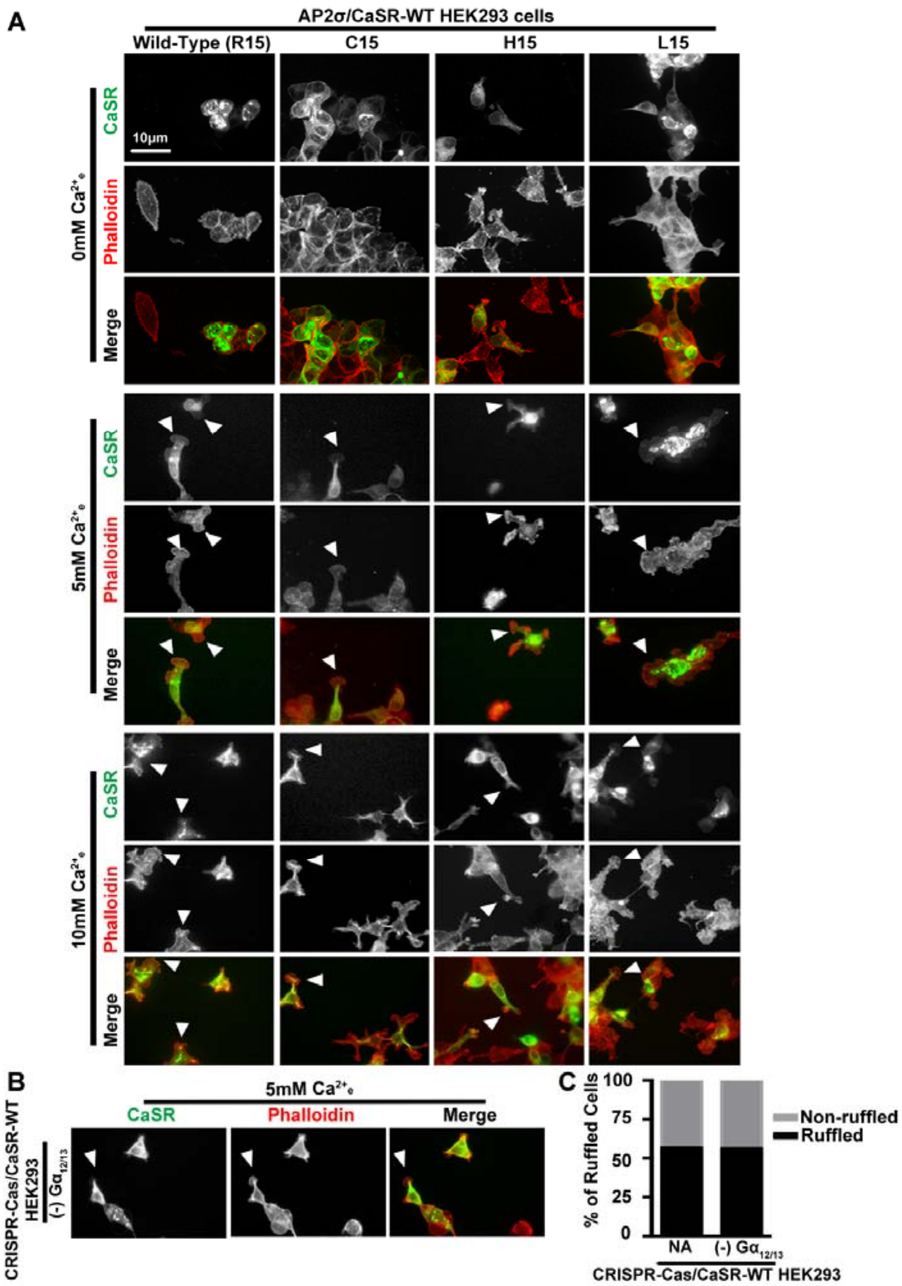

(A) Representative images of Ca^{2+}_{e}-induced membrane ruffling in AP2σ-wild-type (WT, R15) or mutant (C15, H15, L15) cells transfected with pEGFP-CaSR-WT to visualize the receptor and Phalloidin-594 as an actin marker (n = 36-50 cells from 9-10 transfections). (B) Representative images of Ca^{2+}_{e}-induced membrane ruffling in CRISPR-Cas generated HEK293 $G\alpha_{12/13}$ knockout cells ((-)$G\alpha_{12/13}$) transfected with pEGFP-CaSR-WT. (C) Percentage of cells with ruffled and non-ruffled membranes, at 5mM Ca^{2+}_{e}, in mutant ((-)$G\alpha_{12/13}$) cells did not differ significantly from that in native (NA) cells, thereby indicating that membrane ruffling, in cells expressing CaSR, can occur in the absence of $G\alpha_{12/13}$. These similar levels of membrane ruffling in $G\alpha_{12/13}$ knockout cells and native cells expressing the CaSR, was associated with increased SRF activity (Figure 3C) and this suggests that: other signaling inputs, which are downstream of $G\alpha_{q/11}$, and affect SRF transcription but not membrane ruffling, may be involved and these may include MAPK pathways via p38, JNK and ERK, that are activated by CaSR and enhance SRF (Kifor et al., 2001; Zhang and Liu, 2002), or Ca^{2+}_{i} that is utilized by muscarinic receptors of Jurkat T-lymphocytes (Lin et al., 2002); and that the SRF reporter assay may be more sensitive than the membrane ruffling assay in detecting subtle changes in signaling pathways.

FIGURE S5 Optimisation of TIRF-M Conditions and Signaling Assays for Observation of ADIS and Endocytosis, and Sustained Signaling, Related, to Figure 4 and 6

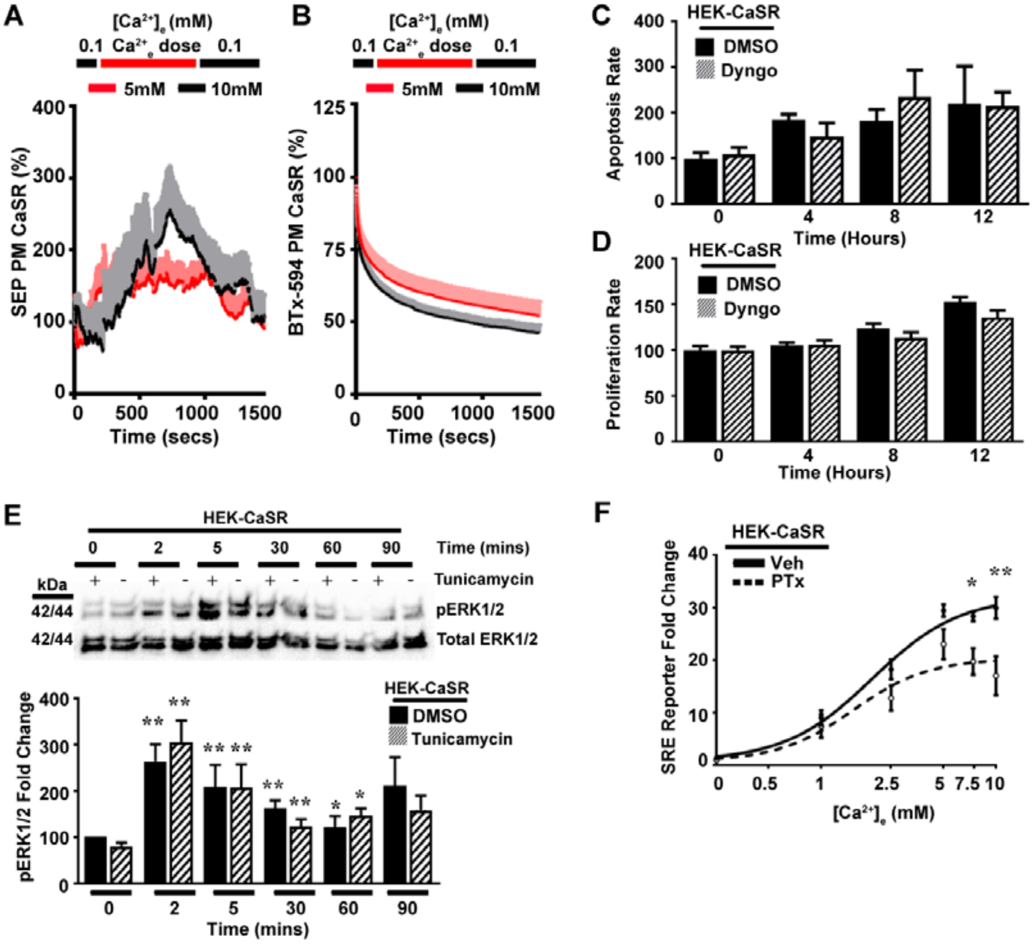

TIRF-M analyses of (A) SEP and (B) BTx-594 in HEK293 cells transiently transfected with BSEP-CaSR-WT, in response to treatment with 5mM or 10mM Ca^{2+}_{e}, to determine optimal conditions to observe ADIS and internalization events. Calcium concentrations are shown above and 'Ca^{2+}_{e} dose' indicates the time when either 5mM or 10mM was added to the cells (n=14). Both 5mM and 10mM doses increased BSEP-CaSR and reduced BTx-594, but this was elevated in cells treated with 10mM Ca^{2+}_{e}. Therefore 10mM was used in subsequent TIRF studies. Treatment of HEK-CaSR cells with Dyngo, and control vehicle (DMSO) did not affect the (C) apoptosis or (D) proliferation rate of cells demonstrating that loss of the sustained pERK1/2 response is not due to Dyngo-mediated changes in cell survival. (E) Western blot analyses of pERK1/2 (top) with densitometric analysis (bottom) following treatment with tunicamycin (+) or DMSO (-). pERK1/2 responses increased over time in tunicamycin-treated cells with the early and sustained responses at 5 minutes and 30 minutes, respectively being present, thereby demonstrating that the sustained signal still arises even though the synthesis pathway is blocked. *$p<0.05$, **$p<0.02$, compared to 0mM response +/- tunicamycin, by 2-way ANOVA analysis. (F) Effect of pertussis toxin (PTx) or ethanol vehicle (veh) on Ca^{2+}_{e}-induced SRE luciferase reporter activity in HEK-CaSR cells. PTx reduced SRE luciferase activity, thereby indicating that $G\alpha_{i/o}$ contributes to the SRE signal. Data shows mean±SEM (n=12); *$p<0.05$ and **$p<0.02$ (2-way ANOVA of veh-treated vs. PTx-treated).

FIGURE S6 Sustained Signal from the CaSR Involves internalization of receptor to Rab5 Endosomes, Related to Figure 6 and 7

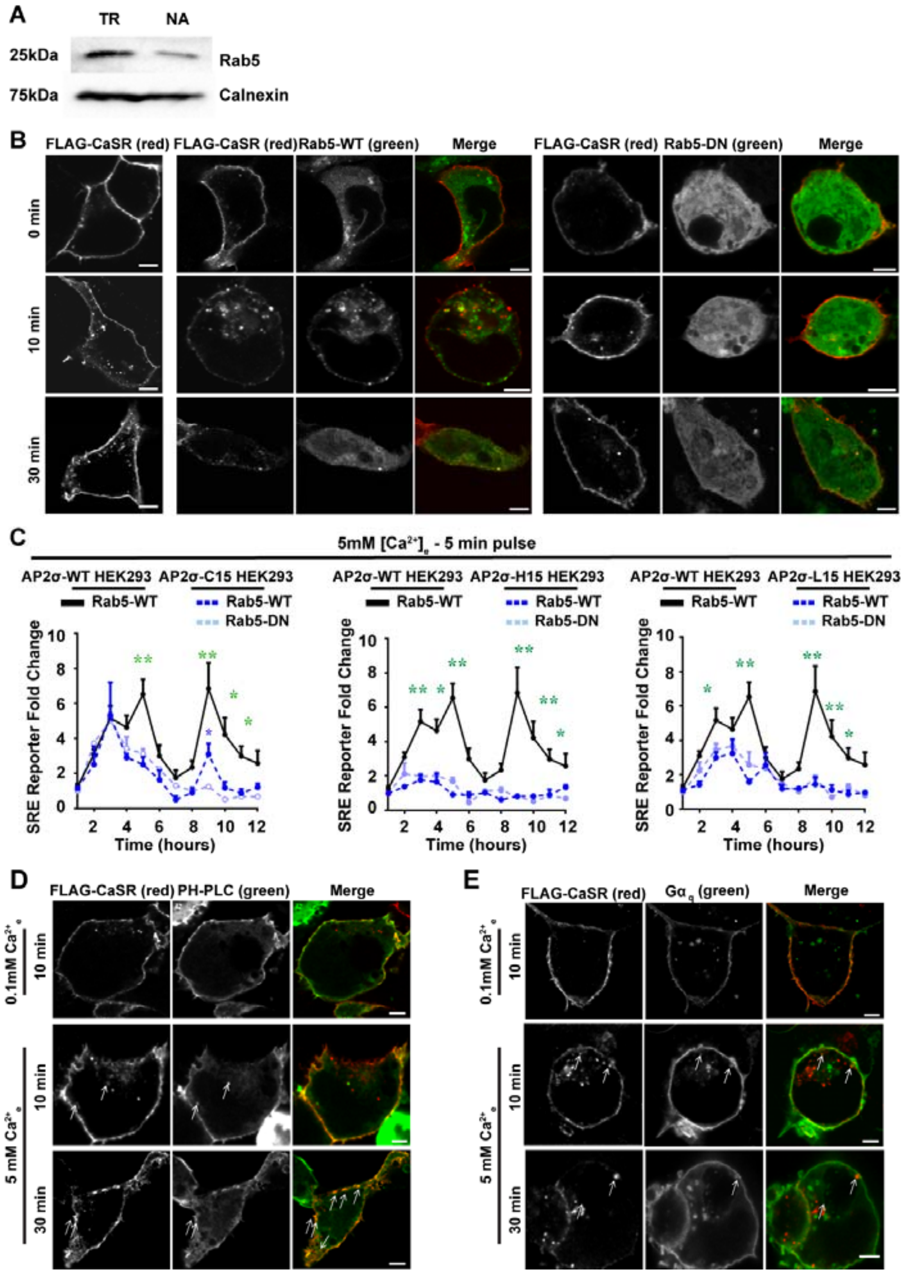

(A) Western blot analysis of cell lysates obtained from native (NA) HEK293 cells for endogenous expression of Rab5, and from HEK293 cells transfected (TR) with mCh-Rab5-WT and overexpressing Rab5. Calnexin was used as a housekeeping protein. (B) Confocal images of HEK293 cells cotransfected with FLAG-CaSR alone (left panel) and with GFP-Rab5-WT or GFP-Rab5-DN. N=8-10 images per construct from 3-4 transfections for B. Scale = 5μm. (C) SRE luciferase reporter response to 5 minute pulses of 5mM Ca^{2+}_e in the presence of Rab5-WT or DN mutant in: AP2σ-WT and AP2σ-mutant cells. Asterisks show AP2σ-WT vs. AP2σ-mutant responses transfected with Rab5-WT (green) and AP2σ-mutant responses in the presence of Rab5-WT vs. Rab5-DN (blue). The Rab5-DN abolished the SRE response in AP2σ-C15 mutant HEK293 cells, thereby indicating that this response requires receptor internalization to Rab5 endosomes. Data are shown as mean+SEM with *$p<0.05$ and **$p<0.02$ (2-way ANOVA). (D-E) Confocal images of cells expressing FLAG-CaSR with either (D) PH-PLC-GFP or (E) $G\alpha_q$-Venus following treatment with 5mM Ca^{2+}_e for 10 and 30 minutes. Merged images reveal partial

colocalization between CaSR and the PLC and Gα_q proteins in internalized structures at both time points (indicated by arrows). Scale = 5µm.

Figure S7 CaSR Sustained Signal involves the PLC-DAG-IP$_3$ pathway but does not require βarrestin proteins, Related to Figure 7

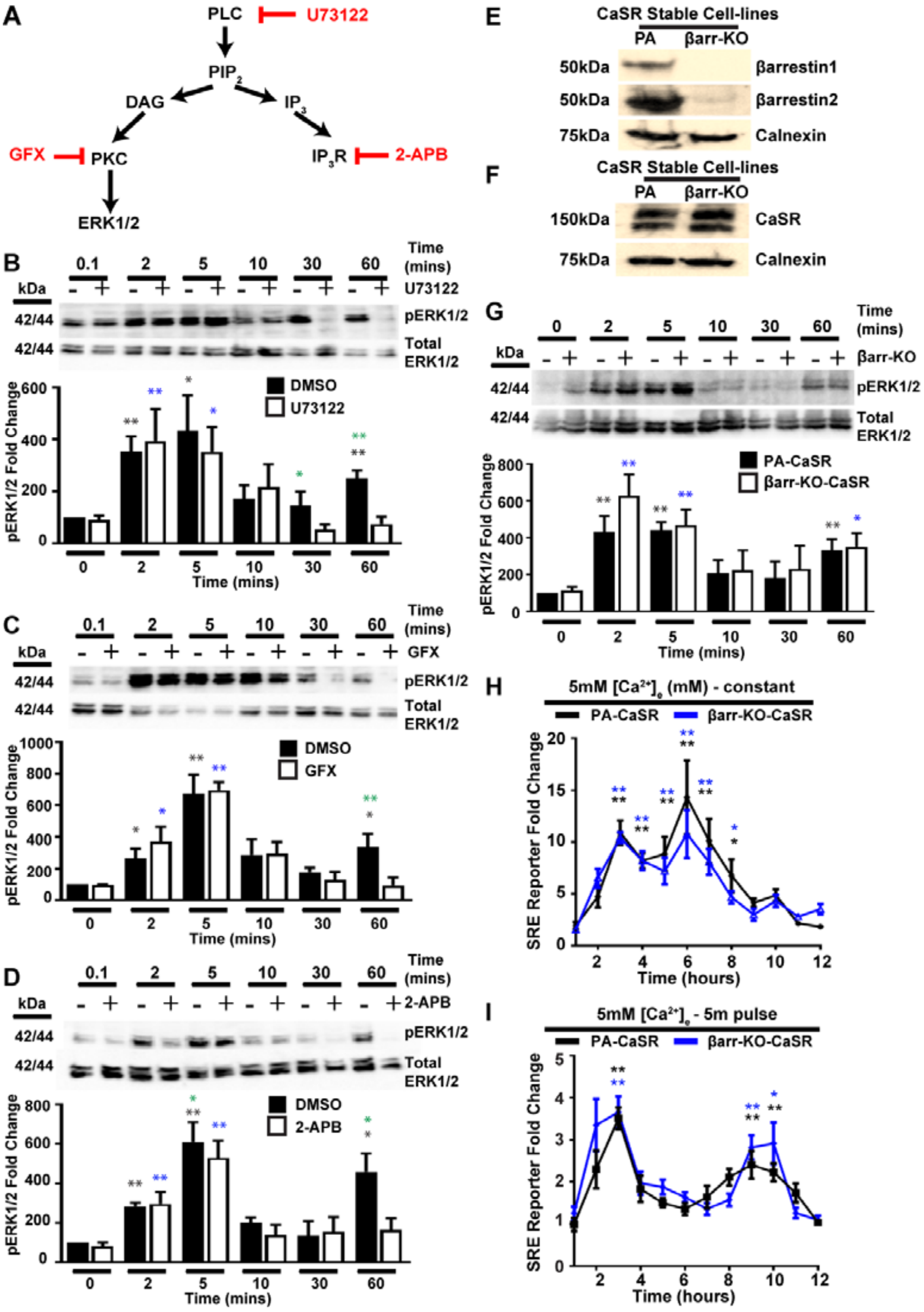

(A) Schematic diagram illustrating the PLC-DAG-IP$_3$ signaling pathway activated by CaSR, and inhibitors (red) used to examine the role of signal components in sustained signaling. PLC, which can be inhibited by U73122, activates PIP$_2$ that activates two second messenger proteins DAG and IP$_3$. PKC, activated by DAG, and the IP$_3$-receptor (IP$_3$R), activated by IP$_3$, are inhibited by the GF-109203X (GFX), and 2-aminoethoxydiphenyl borate (2-APB) compounds, respectively. (B-D) Western blot analyses of pERK1/2 (top) with densitometric analysis (bottom) in HEK-CaSR cells treated with (B) U73122, (C) GFX, or (D) 2-APB. Cells were treated with inhibitor (+) or DMSO (-) and given a 5 minute pulse of 5mM Ca^{2+}_e. The sustained pERK1/2 response was impaired by

U73122, GFX and 2-APB, indicating that the PLC-DAG-IP_3 pathway is involved in the generation of the CaSR sustained signal. *p<0.05, **p<0.02, by 2-way ANOVA analysis, comparing: responses at 0 mins compared to other time points in DMSO treated cells (black asterisk), responses at 0 mins compared to other time points in U73122, GFX or 2-APB treated cells (blue asterisk), and DMSO vs. U73122, GFX or 2-APB (green asterisk) at each time point. (E) Confirmation of βarrestin-1 and βarrestin-2 protein expression in parental (PA) HEK293 cells stably expressing CaSR, and loss of their expression following deletion in CRISPR-Cas-generated CaSR-HEK293 cell-lines (βarr-KO), by Western blot analysis. (F) Stable overexpression of wild-type CaSR protein in parental HEK293 cells (PA-CaSR) and βarr-KO cells (βarr-KO-CaSR) shown by Western blot analysis. (G) Western blot analyses of pERK1/2 (top) with densitometric analysis (bottom) in the PA-CaSR ((-) in upper panel and PA-CaSR in lower panel) and βarr-KO-CaSR ((+) in upper panel and βarr-KO-CaSR in lower panel) cell-lines following a 5 minute pulse of 5mM Ca^{2+}_e. pERK1/2 responses increased over time with the early and sustained responses being present in both cell-lines, thereby demonstrating that βarrestin-1 and -2 are not required for sustained responses. (H) SRE luciferase reporter responses to treatment with 5mM Ca^{2+}_e over 12 hours in PA-CaSR or βarr-KO-CaSR cells (n=8). (I) SRE luciferase reporter response to 5 minute pulses of 5mM Ca^{2+}_e in PA-CaSR or βarr-KO-CaSR cells (n=8). Data shows mean±SEM. *p<0.05, **p<0.02. Statistical analyses by 2-way ANOVA, comparing: responses at 0 mins compared to other time points in PA-CaSR cells (black); and responses at 0 mins compared to other time points in βarr-KO-CaSR cells (blue). Densitometric analyses were performed on at least 4 blots from independent lysates.

Supplemental Items

Table S1 List of Oligonucleotide Primer Sequences, Related to Figure S1

REAGENT or RESOURCE	SOURCE	IDENTIFIER
**Genomic Primers**		
AP2S1 Exon 2 Forward	Nesbit *et al*	AGCCCTATCTCCCCTCTGG
AP2S1 Exon 2 Reverse	Nesbit *et al*	GAAGCAAGCAAGCTCAAAGC
**cDNA Primers**		
AP2S1 cDNA Sequencing Primer Forward	Nesbit *et al*	GAAGTCCGCTCTAGCTCTGG
AP2S1 cDNA Sequencing Primer Reverse	Nesbit *et al*	GTTTCAGCACCTTCGTCGG
Forward Primer to generate pcDNA3.1-HisV5 construct	SigmaAldrich	GCCGGATCCATGATCCGCTTTATCCTC
Reverse Primer to generate pcDNA3.1-HisV5 construct	SigmaAldrich	ggccgcGATATCCGTAGAATCGAGACCGAGGAGAGGGTTAGGGATAGGC TTACCTTCGAACCGGCACTCCAGGGACTGTAGCAT
pcDNA3.1 T_7 Primer	SigmaAldrich	TAATACGACTCACTATAGGG
pcDNA3.1 Reverse	SigmaAldrich	TAGAAGGCACAGTCGAGG
Rab5-S34N site-directed mutagenesis Forward	SigmaAldrich	TCCGCTGTTGGCAAAAATAGCCTAGTGCTTCGT
Rab5-S34N site-directed mutagenesis Reverse	SigmaAldrich	ACGAAGCACTAGGCTATTTTTGCCAACAGCGGA
Rab5-Q79L site-directed mutagenesis Forward	SigmaAldrich	TGGGATACAGCTGGTCTTGAACGATACCATAGCC
Rab5-SQ79L site-directed mutagenesis Reverse	SigmaAldrich	GGCTATGGTATCGTTCAAGACCAGCTGTATCCCA
AP2S1 cDNA primer to introduce silent mutations for siRNA sites Forward 1	SigmaAldrich	CGCCTGGCCTTGTGGTATATGCAGTTTGATGAT
AP2S1 cDNA primer to introduce silent mutations for siRNA sites Reverse 1	SigmaAldrich	ATCATCAAACTGCATATACCACAAGGCCAGGCG
AP2S1 cDNA primer to introduce silent mutations for siRNA sites Forward 2	SigmaAldrich	GCCTTGTGGTATATGCAATTCGACGATGATGAG
AP2S1 cDNA primer to introduce silent mutations for siRNA sites Reverse 2	SigmaAldrich	CTCATCATCGTCGAATTGCATATACCACAAGGC
AP2S1 cDNA primer to introduce silent mutations for siRNA sites Forward 3	SigmaAldrich	AGAGACCAGCCAAACCAAAGTACTGAAACAGC
AP2S1 cDNA primer to introduce silent mutations for siRNA sites Reverse 3	SigmaAldrich	GCTGTTTCAGTACTTTGGTTTGGCTGGTCTCT
AP2S1 cDNA primer to introduce silent mutations for siRNA sites Forward 4	SigmaAldrich	CCAGCCAAACCAAAGTACTAAAGCAACTGCTGATG
AP2S1 cDNA primer to introduce silent mutations for siRNA sites Reverse 4	SigmaAldrich	CATCAGCAGTTGCTTTAGTACTTTGGTTTGGCTGG

AP2S1 cDNA primer to introduce silent mutations for siRNA – introduce WT	SigmaAldrich	CGGGCAGGCAAGACGCGCCTGGCCAAATGGTAT
AP2S1 cDNA primer to introduce silent mutations for siRNA – introduce C15	SigmaAldrich	CGGGCAGGCAAGACGTGCCTGGCCAAATGGTAT
AP2S1 cDNA primer to introduce silent mutations for siRNA – introduce H15	SigmaAldrich	CGGGCAGGCAAGACGCACCTGGCCAAATGGTAT
AP2S1 cDNA primer to introduce silent mutations for siRNA – introduce L15	SigmaAldrich	CGGGCAGGCAAGACGCTCCTGGCCAAATGGTAT
**qRT-PCR Primers**		
GAPDH (Hs_GAPDH_1_SG)	Quantitect, Qiagen	Cat#QT00079247
CCND1 (Hs_CCND1_1_SG)	Quantitect, Qiagen	Cat#QT00495285
PGK1 (Hs_PGK1_1_SG)	Quantitect, Qiagen	Cat#QT00013776
CANX (Hs_CANX_1_SG)	Quantitect, Qiagen	Cat#QT00092995
TBP1 (Hs_TBP_1_SG)	Quantitect, Qiagen	Cat#QT00000721
AP2A1 (Hs_AP2A1_1_SG)	Quantitect, Qiagen	Cat#QT00071715
AP2A2 (Hs_AP2A2_1_SG)	Quantitect, Qiagen	Cat#QT00005572
AP2B1 (Hs_AP2B1_1_SG)	Quantitect, Qiagen	Cat#QT01677494
AP2M1 (Hs_AP2M1_1_SG)	Quantitect, Qiagen	Cat#QT00089334
AP2S1 (Hs_AP2S1_1_SG)	Quantitect, Qiagen	Cat#QT01155238
**siRNA**		
siRNA targeting sequence to AP2S1	SantaCruz Biotechnology	sc-97710

Table S2 List of Antibodies, related to Figure S1, S3 and S6

REAGENT or RESOURCE	SOURCE	IDENTIFIER
Mouse monoclonal anti-CaSR (clone 5C10, ADD)	Abcam	Cat#ab19347, RRID: AB_444867
Mouse monoclonal anti-AP2α	BD Transduction Laboratories	Cat#610501, RRID: AB_397867
Mouse monoclonal anti-AP2β	BD Transduction Laboratories	Cat#610381, RRID: AB_397764
Rabbit polyclonal anti-pERK1/2	Cell Signalling Technology	Cat#9101L, RRID: AB_331646
Rabbit polyclonal anti-total ERK1/2 (clone 137F5)	Cell Signalling Technology	Cat#4695S, RRID: 390779
Rabbit polyclonal anti-Calnexin	Millipore	Cat#AB2301, RRID: AB_10948000
Rabbit polyclonal anti-AP2σ	Abcam	Cat#ab173201, RRID:AB_2631096
Rabbit monoclonal anti-AP2μ	Abcam	Cat#ab7995, RRID:AB_1309955
Rabbit polyclonal anti-Rab5	Abcam	Cat#ab18211, RRID:AB_470264
Rabbit polyclonal anti-βarrestin-1	Genetex	Cat#30065, RRID:AB_2715557
Mouse monoclonal (H-9) anti-βarrestin-2	SantaCruz Biotechnology	Cat#sc-13140, RRID:AB_626701
Phalloidin-594	ThermoFisher Scientific	Cat#A12381 RRID:AB_2315633
Donkey anti-rabbit Alexa Fluor 488	Molecular Probes	Cat# A-11094, RRID:AB_221544
Donkey anti-rabbit Alexa Fluor 555	Molecular Probes	Cat# ab150074, RRID:AB_2636997
Transferrin-594	Molecular Probes	Cat#T13343, RRID:AB_2716742
Goat anti-rabbit IgG-HRP conjugate	Biorad	Cat#1706515, RRID: AB_11125142
Donkey anti-mouse IgG-HRP conjugate polyclonal	SantaCruz Biotechnology	Cat#sc-2318, RRID: AB_641171

Supplemental Experimental Procedures

Cell Culture

HEK293 cells were cultured in DMEM Glutamax media (Gibco) with 10% fetal bovine serum (FBS) (Gibco), and those stably expressing either CaSR or AP2σ proteins were supplemented with 400µg geneticin (Gibco). HEK293 cells with deletion of *GNAQ*, *GNA11*, *GNA12*, and *GNA13* genes, encoding $G\alpha_q$, $G\alpha_{11}$, $G\alpha_{12}$ and $G\alpha_{13}$, respectively; or combined deletions of *ARRB1 and ARRB2* encoding βarrestin-1 and βarrestin-2, respectively, were generated by gene-editing using CRISPR-Cas (Devost et al., 2017). All transfections were performed with Lipofectamine 2000 (Invitrogen). AP2σ stable cells were pre-treated with 100nM AP2σ siRNA (SantaCruz Biotechnology) prior to performing functional assays. For functional studies, AP2σ cells were transiently transfected with pEGFP-CaSR-WT (Pearce et al., 1996). All studies were performed in plates pre-treated with poly-L-lysine (Sigma).

Human Lymphoblastoid Cells from FHH3 Patients

Informed consent was obtained from individuals using protocols approved by local and national ethics committees, London, UK (MREC/02/2/93). Epstein-Barr virus (EBV) transformed lymphoblastoid cells were established using methods previously described (Parkinson and Thakker, 1992), using leukocytes, from four affected and four unaffected members of the FHH3 kindred with AP2σ-C15 mutations (Nesbit et al., 2013). Lymphoblastoid cells were maintained in RPMI-1640 media supplemented with 10% FBS and penicillin/streptomycin (Gibco).

Construction of Stable Cell-lines

HEK293 cell-lines stably expressing full-length CaSR (HEK-CaSR) have been previously described (Nesbit et al., 2013). HEK293 cells with deletion of βarrestin-1 and βarrestin-2 (βarr-KO) that stably express the CaSR were generated using a pcDNA3.1 construct (Invitrogen) containing full-length CaSR cDNA, as previously described (Nesbit et al., 2013). HEK293 cell-lines stably expressing either AP2σ-wild-type (WT) or AP2σ-mutant proteins (C15, H15 or L15) were generated using a pcDNA3.1 construct containing full-length AP2σ cDNA with a C-terminal V5 epitope. Silent mutations were introduced to render the construct insensitive to AP2σ-targeted siRNA (SantaCruz Biotechnology). Oligonucleotide sequences used for construct generation and introduction of silent mutations were purchased from Sigma (Table S1). pcDNA3.1-AP2σ-V5 constructs were transfected into HEK293 cells and cultured in geneticin selection media. Individual pcDNA3.1-AP2σ-V5 positive clones were picked and subcultured in fresh selection media. AP2σ expression was assessed by Western Blot analysis. HEK293 cells were chosen as a model cell system to examine trafficking of the CaSR for the following reasons: i) HEK293 have been previously used and established as a model to assess CaSR function; ii) HEK293 cells are used as appropriate cultures of parathyroid and CaSR-expressing kidney cell-lines are not available; iii) the ADIS phenomenon of CaSR was first described in HEK293 cells (Grant et al., 2011); iv) we have previously demonstrated AP2σ-mutant protein effects on intracellular calcium using HEK293 cells (Nesbit et al., 2013); and, v) HEK293 cells transfected with CaSR respond to calcium in a concentration-dependent manner and utilize previously characterized pathways of intracellular calcium release, MAPK activation and cAMP signaling (Conigrave and Ward, 2013). Mutations within the constructs were introduced by site-directed mutagenesis using the Quikchange Lightning XL or Multi kits (Agilent Technologies).

Confirmation of Mutations and DNA Sequencing

The DNA sequence abnormalities in the lymphoblastoid cell-lines were confirmed by using extracted DNA (Gentra Puregene Blood Kit (Qiagen)) and PCR amplification of *AP2S1* exon 2, followed by restriction endonuclease analysis utilizing *HhaI* (New England Biolabs), as previously described (Nesbit et al., 2013). Presence of mutations within constructs were verified using dideoxynucleotide sequencing with the BigDye Terminator v3.1 Cycle Sequencing Kit (Life Technologies) and an automated detection system (ABI3730 Automated capillary sequencer; Applied Biosystems) (Gorvin et al., 2017a; Newey et al., 2013). The oligonucleotide sequences that were used are listed in Table S1.

Western Blot Analysis

The antibodies used for Western blot analysis are listed in Table S2. For studies of protein expression, cells were lysed in NP40 lysis buffer (50mM Tris HCl pH7.4, 1mM EDTA, 150mM NaCl, protease inhibitors) (Newey et al., 2013), lysates were resuspended in Laemmli buffer, boiled and separated on 6% and 12% sodium-dodecyl sulphate (SDS) polyacrylamide gel electrophoresis gels. Following transfer to polyvinylidene difluoride membrane (Amersham), blots were blocked in 5% BSA/TBS-t (pERK1/2 studies) or marvel/TBS-t, then probed with the primary and secondary antibodies. Blots were visualized using the Immuno-Star WesternC kit (BioRad) on a BioRad Chemidoc XRS+ system (Newey et al., 2013). Following development, blots were stripped with Restore Western blot stripping buffer (Thermo Scientific), blocked in marvel/TBS-t and reprobed with primary

antibodies. For studies of AP2 subunit expression, cells were probed with the AP2σ antibody, followed by calnexin (used as a housekeeping protein), AP2μ, AP2β, then AP2α, with stripping of blots between each antibody. For all other Western blots, calnexin was used as the housekeeping protein after initial probing with the gene of interest.

For sustained signaling studies, cells were stimulated with 5mM $CaCl_2$ for 5 minutes, followed by incubation in media containing 0mM $CaCl_2$ for 0-60 minutes. For studies with 30μM Dyngo-4a (Abcam) (Jean-Alphonse et al., 2014), cells were pre-incubated for 30 minutes. For studies with 5μM U73122 (Sigma), 1μM GF-109203X (Sigma), 100 μM 2-aminoethoxydiphenyl borate (2-APB) (Sigma), or 5μg/mL tunicamycin (Sigma) (Avlani et al., 2013; Grant et al., 2011; Luo et al., 2001), compounds were added to the media and cells were incubated after calcium stimulation. After analysis for pERK1/2, blots were stripped and reprobed with an anti-total ERK1/2 antibody (Gorvin et al., 2017a). For studies of Rab5 contribution to sustained signaling, 100ng/ml mCh-Rab5-WT (Addgene plasmid ♯49201), or mCh-Rab5-dominant negative (DN, S34N) or –constitutively active (CA, Q79L) (generated by site-directed mutagenesis using oligonucleotides listed in Table S1) were transfected 48-hours before Western blot analysis. Densitometry analysis was performed using ImageJ and statistical analyses were performed by 2-way ANOVA using Graphpad Prism 6.

Quantitative RT-PCR (qRT-PCR) Analysis
First-strand cDNA was generated using the Quantitect reverse transcription kit (Qiagen) from 1μg total RNA from each lymphoblastoid cell-line extracted using the MirVana (Ambion) kit (Gorvin et al., 2013). All qRT-PCR test samples were normalized to levels of the geometric mean of five reference genes, *GAPDH*, *CCND1*, *PGK1*, *CANX* and *TBP1.* Primers were obtained from Quantitect (Qiagen). Threshold cycle (CT) values were obtained from the start of the log phase on Rotorgene Q Series Software, and CT values analyzed in Microsoft Excel 2011 (Gorvin et al., 2013)and graphs generated using GraphPad Prism 6. Studies were performed in 4 biological replicates each in quadruplicate. Data was analyzed by Student's t-test.

Uptake Assays
Transferrin uptake assays were performed in native HEK293 cells and AP2σ-WT and AP2σ-mutant HEK293 cells. Transferrin assays were performed as previously described (Gorvin et al., 2013). Cells were seeded in 24-well plates, transfected with pEGFP-CaSR-WT to yield AP2σ-WT/CaSR-WT or AP2σ-mutant/CaSR-WT HEK293 cells, and incubated for 24 hours. Cells were incubated in serum-free media (SFM) prior to treatment with 5μg/mL transferrin conjugated to Alexa Fluor 594 (Molecular Probes) for 0 or 10 minutes. Following incubation, cells were washed in PBS and lysed in NP40 buffer. Fluorescence was measured using a CytoFluor microplate reader (PerSeptive Biosystems) at 580nm excitation and 615nm emission wavelengths. Cell surface expression of β2AR was performed in AP2σ-WT and AP2σ-mutant HEK293 cells using an ELISA-based assay and a β2AR-FLAG construct (Jean-Alphonse et al., 2014) using methods adapted from previously reported studies (Grant et al., 2011; Nesbit et al., 2013). Surface expression was assessed in cells treated with 0μM or 10 μM isoproterenol for 30 minutes. Fluorescence was measured using the PHERAstar FS microplate reader (BMG Labtech). Total fluorescence was normalized to total cellular protein measured by Coomassie Bradford Assay (Pierce). Data was normalized to uptake at 0mins in AP2σ-WT cells. Statistical analyses were performed using 2-way ANOVA (Microsoft Excel 2011 and GraphPad Prism 6).

Single-Cell Ca^{2+} Microfluorimetry
Single-cell microfluorimetry experiments were performed in AP2σ-WT or AP2σ-mutant HEK293 cells. Cells were transiently transfected with pEGFP-CaSR-WT. Cells were plated on coverslips 12 hours prior to imaging and incubated in extracellular solution composed of: 140mM NaCl, 5mM KCl, 1.2mM $MgCl_2$, 1mM NaH_2PO_4, 5mM $NaHCO_3$, 10mM HEPES, 10mM glucose, pH7.4. The appropriate $CaCl_2$ concentration was added and adjusted to maintain osmolality to 324.4 mOsm/L. Experiments were performed at 37°C. Fura-2 dye (Life Technologies) was dissolved in DMSO containing 0.03% F127-Pluronic (Sigma). Cells were loaded with 4μM Fura-2 (Molecular Probes) in extracellular solution for 30 minutes at room temperature. Imaging experiments were performed on a Zeiss Axioscope FS2 wide-field microscope with a 40x/1.3 objective. Cells were continuously perfused with extracellular bath solutions, with concentrations of $CaCl_2$ being increased every 2.5 minutes. Cells were imaged initially for the presence of DsRed2 fluorescence, followed by live capture of Fura-2 using 340/380nm excitation and 510nm emission, acquired every 30 seconds using a Hamamatsu OrcaR2 CCD camera controlled by μManager software (Edelstein et al., 2014) and analyzed using ImageJ (NIH) (Schneider et al., 2012). Fura-2 ratios were calculated in Microsoft Excel 2011 and graphs generated using GraphPad Prism 6. Nonlinear regression of concentration-response curves was performed with GraphPad using the normalized response at each $[Ca^{2+}]_e$ for each separate experiment for the determination of EC_{50} (i.e. $[Ca^{2+}]_e$ required for 50% of the maximal response). The maximal signaling response was measured as a fold-change of the peak transient Ca^{2+}_i response to each $[Ca^{2+}]_e$. The EC_{50} values were compared using the *F*-test and the maximal signaling

responses assessed using the Mann-Whitney *U* test. The number of oscillating cells at each $[Ca^{2+}]_e$ was calculated as a percentage of that in WT cells. Statistical analyses were performed using the χ^2 test.

AlphaScreen Assays

AlphaScreen assays (PerkinElmer) were performed in AP2σ-WT or AP2σ-mutant HEK293 cells, HEK-CaSR, HEK293 or lymphoblastoid cells. Assays were performed in 48-well plates and AP2σ-WT and AP2σ-mutant cells transiently transfected with 200ng pEGFP-CaSR 48-hours prior to performance of assays. For pERK1/2 assays, cells were incubated in SFM 12 hours prior to 5 minute treatment with 0-15mM $CaCl_2$. Cells were then lysed in Surefire lysis buffer and pERK1/2 and total ERK1/2 assays performed as previously described (Gorvin et al., 2017a). For cAMP assays involving AP2σ and CaSR, cells were treated with 10μM forskolin for 30 minutes prior to $CaCl_2$ treatment in stimulation buffer (1x Hanks Buffered Saline Solution, 0.1% BSA, 0.1% 3-isobutyl-1-methylxanthine (IBMX), 0.5mM HEPES) plus 0-10mM $CaCl_2$. For studies with PTx, cells were pre-treated with 300ng/mL PTx or vehicle (ethanol) for 6 hours. For studies with UBO-QIC, cells were pre-treated with 1μM UBO-QIC or vehicle (DMSO) for 2 hours. For inhibitor studies, cells were pre-treated with: 15μM gallein or vehicle (DMSO) for 15 minutes (Grant et al., 2011). Cells were incubated with 0-10mM $CaCl_2$ for 15 minutes, then lysed in a HEPES-based solution and AlphaScreen assays performed. For studies of β2AR, cells were transiently transfected with β2AR-FLAG and cells were incubated with 10μM isoproterenol (Sigma) for 30 minutes, then lysed and AlphaScreen assays performed. The fluorescence signal in both assays was measured using the PHERAstar FS microplate reader (BMG Labtech) (Newey et al., 2013). A minimum of 4 independent biological replicates were used. Statistical analysis was performed by 2-way ANOVA with Tukey's multiple-comparisons test using Microsoft Excel 2011, and Graphpad Prism 6.

IP_1 Assays

IP_1 assays were performed in AP2σ-WT and AP2σ-mutant HEK293 cells. IP_1 assays were performed in 24-well plates and cells transiently transfected with 200ng pEGFP-CaSR 48-hours prior to performance of assays. At 24-hours prior to experiments, cells were re-plated in a 384-well plate, and 12-hours later, media changed to serum-free media. IP_1 homogenous time-resolved fluorescence (HTRF) assays (Cisbio) were performed according to manufacturer's instructions, and as previously described (Zhang et al., 2014). Cells were incubated for 5 minutes with stimulation buffer containing a single dose of $CaCl_2$ (between 0.1-10mM), followed by lysis in the supplied lysis buffer. Plates were read on a PHERAStar FS microplate reader one hour later (BMG Labtech).

Luciferase Reporter Assays

Luciferase reporter assays were performed in AP2σ-WT and AP2σ-mutant HEK293 cells, CRISPR-Cas generated HEK293 $G\alpha_{q/11}$, $G\alpha_{12/13}$ and $G\alpha_{q/11/12/13}$ cells, βarr-KO cells or HEK-CaSR cells. Cells were plated in 24-well plates and transiently transfected with 100ng/ml luciferase reporter constructs (either pGL4-NFAT, pGL4-SRE, or pGL4-SRF) and 10ng/ml pRL. For studies in AP2σ-WT and mutant cells, and CRISPR-Cas cells, 100ng/ml pEGFP-CaSR-WT was transfected simultaneously with luciferase reporter and pRL constructs. For studies of Rab5, 100ng/ml mCh-Rab5-WT or Rab5-mutant (DN or –CA) were transfected simultaneously with luciferase reporter and pRL constructs. For all studies, cells were treated with SFM 36 hours after transfection. On the day of the experiment, cells were treated with SFM containing 0-10mM $CaCl_2$ for 4 hours (for concentration-response studies), or, for sustained signaling studies, with SFM containing one of four additions: i) 0mM $CaCl_2$; ii) 5mM $CaCl_2$ for the duration of the experiment (constant); iii) 5 minute pulse of 5mM $CaCl_2$ followed by 0mM $CaCl_2$ with vehicle for the duration of the experiment; or iv) 5 minute pulse of 5mM $CaCl_2$ followed by 0mM $CaCl_2$ with 30μM Dyngo-4a for the duration of the experiment. DMSO was used as the vehicle. Cells were lysed at the end of the 4-hour incubation period for concentration-response studies, or one plate each hour for 12 hours for sustained signaling studies, and luciferase assays performed using Dual-Glo Luciferase (Promega), on a Veritas Luminometer (Promega), as previously described (Gorvin et al., 2017a). Cells were pre-incubated with 1μM UBO-QIC or DMSO for 2 hours, to study $G\alpha_{q/11}$, or for $G\alpha_{i/o}$, cells were treated with 10μM forskolin (MP Biomedicals) and 300ng/ml pertussis toxin (PTx) (Sigma) or vehicle (ethanol diluent) for 6 hours (Avlani et al., 2013), prior to SRE measurement where appropriate. Luciferase:renilla ratios were expressed as fold changes relative to responses at 0mM $CaCl_2$ responses. All assay conditions were performed in 4-8 biological replicates. Statistical analyses were performed using 2-way ANOVA (Microsoft Excel 2011 and GraphPad Prism 6).

Membrane Ruffling

Membrane ruffling studies were performed in AP2σ-WT and AP2σ-mutant HEK293 cells and HEK293 native and CRISPR-Cas $G\alpha_{12/13}$ cells. Cells were plated in 6-well plates on coverslips and transiently transfected with pEGFP-CaSR-WT. Following 24 hour incubation, serum-free media was applied and cells incubated overnight. Methods were adapted from protocols previously described (Bouschet et al., 2007; Davey et al., 2012). Cells were treated with serum-free media containing either 0, 5 or 10mM $CaCl_2$ for 5 minutes, followed by fixing in 4% paraformaldehyde/PBS, permeabilization with triton-X100/PBS, and staining with 4U/mL Phalloidin conjugated

to Alexa Fluor 594 (Molecular Probes). Coverslips were imaged on a Nikon Eclipse E400 wide-field microscope. Cells were classified as ruffled if they fulfilled three criteria: colocalization of CaSR and phalloidin-594, which stains actin; presence of at least two ruffles; and cells were singlets or if in clusters had at least two sides exposed. Up to 10 images of each coverslip were taken, and 82-313 cells were imaged over 15-45 independent transfections. Statistical analyses were performed using χ^2 tests (Microsoft Excel 2011 and GraphPad Prism 6).

Total Internal Reflection Fluorescence Microscopy (TIRF-M)

TIRF-M studies were performed in AP2σ-WT or AP2σ-mutant HEK293, and CRISPR-Cas $G\alpha_{q/11}$ knockout cells. Cells were transfected with BSEP-CaSR (Grant et al., 2011) 24-hours prior to recordings. For clathrin studies, DsRed2-Clathrin was transfected simultaneously with pEGFP-CaSR-WT. Methods for TIRF-M were adapted from previous studies (Grant et al., 2011; Hoppa et al., 2009). Images were obtained with a customized Olympus IX-81 TIRF microscope equipped with a 60×/1.45 Apo lens (Olympus). The 488nm line of an argon ion laser (Melles Griot) was used to excite SEP-CaSR, and a 561nm line of a steady-state diode laser was used to excite BTx-594 or DsRed-Clathrin. Two excitation ports with separate focal pathways were used to make independent adjustments of each laser. The emission pathway for both fluorescent reporters was imaged simultaneously with an image splitter (Dual View, Optical Insights) generating side-by-side images of both emission wavelengths on the chip of an electron multiplying charge-coupled device (EMCCD) camera (Cascade II 512B, Roper Scientific). Alignment of the two emission channels was corrected on a daily basis by imaging 200nm tetraspeck beads (Molecular Probes). Extracellular medium for imaging consisted of 140mM NaCl, 5mM KCl, 0.55 $MgCl_2$, 10mM HEPES and 10mM D-glucose, pH 7.4. The appropriate $CaCl_2$ concentration was added and adjusted with NaCl. All solutions were osmotically balanced. Experiments were performed at 37°C. To monitor CaSR internalization, cells were incubated with 5μg/mL BTx-594 for 3 minutes prior to imaging. Experiments were performed with continuous imaging of cells perfused with basal 0.1mM $CaCl_2$ imaging solution for 1 minute, followed by addition of 10mM $CaCl_2$ imaging solution for 10 minutes. The following 9 minutes were conducted in basal solution. Solutions took 45 seconds from switching the perfusion to reaching the chamber. These methods were chosen following initial experiments in cells using 5mM or 10mM Ca^{2+}_e as the stimulant, which demonstrated more robust responses with 10mM Ca^{2+}_e. Images were captured at 10 frames/sec in BSEP studies. Images were acquired using Cell^R software (Olympus) and analyzed with ImageJ. Fluorescence intensity was measured in each frame of captured movies at both emission wavelengths, and green fluorescence intensity normalized to red fluorescence to acquire total surface CaSR.

For CaSR and clathrin studies, one frame of BSEP-CaSR and DsRed-Clathrin was imaged every 3 seconds for 10 minutes. Cells were initially bathed in basal $CaCl_2$ solution followed by 10mM solution for 9 minutes. CaSR and clathrin positive vesicles were assessed for colocalisation by measuring fluorescence intensity at each emission wavelength. CaSR-clathrin puncta were analyzed if they adhered to the following criteria adapted from previous studies (Mattheyses et al., 2011): 1) CaSR and clathrin colocalized for at least 4 frames (12 secs); 2) Clathrin puncta are diffraction limited; 3) spots disappear simultaneously before the end of the imaging period; 4) vesicle does not merge with other regions of fluorescence. Vesicles with no, or limited movement, during the duration of the movie were classified as non-motile, while those that moved from their original position over a span of two or more frames were classified as motile vesicles (Mukhopadhyay et al., 2011). Vesicles were tracked using ImageJ.

Data from individual cells was averaged over 30-44 independent experiments for AP2σ studies, 15-25 experiments for CRISPR-Cas studies and 95-200 vesicles in 14-16 cells for clathrin studies and analyzed in Microsoft Excel 2011. Graphs were generated in GraphPad Prism 6.

Confocal Microscopy

Confocal imaging was performed in native HEK293 cells using methods adapted from previous studies (Bouschet et al., 2007; Hanyaloglu et al., 2005). Cells were transfected with FLAG-CaSR, GFP-Rab5-WT, GFP-Rab5-DN, PH-PLC-GFP or $G\alpha_q$-Venus. Prior to all studies, cells were incubated in low calcium Earle's solution (140mM NaCl, 5mM KCl, 0.5mM $CaCl_2$, 0.8mM $MgCl_2$, 25mM HEPES pH 7.4, 1M glucose) for 30 minutes. To label receptors, cells were 'fed' with mouse anti-FLAG antibody (Sigma) for 15 minutes prior to ligand stimulation. Cells were fixed in 4% paraformaldehyde/PBS (Sigma) for 20 minutes, permeabilised with 0.2% triton-X100/PBS (Thermo Scientific), followed by immunostaining with secondary antibodies Alexa Fluor 488 or Alexa Fluor 555 (both Molecular Probes). For FLAG-CaSR, cells were incubated with and without 5mM Ca^{2+}_e for 10 or 30 minutes. Cells were mounted in ProLong Gold Antifade Mountant (Life Technologies) or Fluoromount-G (Thermo Fisher). Images were captured using a confocal, laser-scanning microscope (Leica SP5) with a Plan-Achromat ×63/1.4 oil DIC objective. Images were analyzed using Leica LAS AF image acquisition software. All subsequent raw image files were analyzed using ImageJ or LAS AF Lite (Leica) to measure level of colocalization.

Pearson's correlation coefficient was calculated for at least 3 regions of interest per cell using the ImageJ plugin JACoP.

Apoptosis and Proliferation Assays

HEK-CaSR cells were plated in 96-well plates and apoptosis and proliferation assessed every 4 hours using the Caspase-Glo 3/7 and CellTiter Blue kits, respectively (Promega). Caspase-Glo 3/7 was measured on a Veritas luminometer, and CellTiter Blue on a CytoFluor microplate reader (PerSeptive Biosystems).

Quantification and Statistical Analyses

Statistical analyses are indicated in the legends of each figure, including the definitions of error bars (e.g. standard error, 95% confidence intervals) and the number of experimental replicates denoted by n. Two-tailed unpaired t test, 2-way ANOVA, Mann-Whitney U-test, χ^2-test, Pearson's correlation coefficient and the F-test were used to calculate statistical significance using Graphpad Prism 6 or ImageJ software. A p value of <0.05 was considered statistically significant. Statistical tests used for each experiment are indicated in the relevant methods section.

Supplemental References

Boucrot, E., Saffarian, S., Zhang, R., and Kirchhausen, T. (2010). Roles of AP-2 in clathrin-mediated endocytosis. PloS one 5, e10597.

Edelstein, A.D., Tsuchida, M.A., Amodaj, N., Pinkard, H., Vale, R.D., and Stuurman, N. (2014). Advanced methods of microscope control using muManager software. Journal of biological methods 1.

Gorvin, C.M., Rogers, A., Stewart, M., Paudyal, A., Hough, T.A., Teboul, L., Wells, S., Brown, S.D.M., Cox, R.D., and Thakker, R.V. (2017b). N-ethyl-N-nitrosourea Induced Adaptor Protein 2 Sigma Subunit 1 (Ap2s1) Mutations Establish Ap2s1 Loss-of-function Mice. JBMR Plus.

Hoppa, M.B., Collins, S., Ramracheya, R., Hodson, L., Amisten, S., Zhang, Q., Johnson, P., Ashcroft, F.M., and Rorsman, P. (2009). Chronic palmitate exposure inhibits insulin secretion by dissociation of Ca(2+) channels from secretory granules. Cell metabolism 10, 455-465.

Lin, K., Wang, D., and Sadee, W. (2002). Serum response factor activation by muscarinic receptors via RhoA. Novel pathway specific to M1 subtype involving calmodulin, calcineurin, and Pyk2. The Journal of biological chemistry 277, 40789-40798.

Luo, D., Broad, L.M., Bird, G.S., and Putney, J.W., Jr. (2001). Signaling pathways underlying muscarinic receptor-induced [Ca2+]i oscillations in HEK293 cells. The Journal of biological chemistry 276, 5613-5621.

Mattheyses, A.L., Atkinson, C.E., and Simon, S.M. (2011). Imaging single endocytic events reveals diversity in clathrin, dynamin and vesicle dynamics. Traffic 12, 1394-1406.

McMurtry, C.T., Schranck, F.W., Walkenhorst, D.A., Murphy, W.A., Kocher, D.B., Teitelbaum, S.L., Rupich, R.C., and Whyte, M.P. (1992). Significant developmental elevation in serum parathyroid hormone levels in a large kindred with familial benign (hypocalciuric) hypercalcemia. The American journal of medicine 93, 247-258.

Mitsunari, T., Nakatsu, F., Shioda, N., Love, P.E., Grinberg, A., Bonifacino, J.S., and Ohno, H. (2005). Clathrin adaptor AP-2 is essential for early embryonal development. Molecular and cellular biology 25, 9318-9323.

Mukhopadhyay, A., Nieves, E., Che, F.Y., Wang, J., Jin, L., Murray, J.W., Gordon, K., Angeletti, R.H., and Wolkoff, A.W. (2011). Proteomic analysis of endocytic vesicles: Rab1a regulates motility of early endocytic vesicles. Journal of cell science 124, 765-775.

Pearce, S.H., Bai, M., Quinn, S.J., Kifor, O., Brown, E.M., and Thakker, R.V. (1996). Functional characterization of calcium-sensing receptor mutations expressed in human embryonic kidney cells. J Clin Invest 98, 1860-1866.

Schneider, C.A., Rasband, W.S., and Eliceiri, K.W. (2012). NIH Image to ImageJ: 25 years of image analysis. Nature methods 9, 671-675.

Zhang, C., Mulpuri, N., Hannan, F.M., Nesbit, M.A., Thakker, R.V., Hamelberg, D., Brown, E.M., and Yang, J.J. (2014). Role of Ca2+ and L-Phe in regulating functional cooperativity of disease-associated "toggle" calcium-sensing receptor mutations. PloS one 9, e113622.

Zhang, W., and Liu, H.T. (2002). MAPK signal pathways in the regulation of cell proliferation in mammalian cells. Cell Res 12, 9-18.
